# Phasevarions Mediate Random Switching of Gene Expression in Pathogenic *Neisseria*

**DOI:** 10.1371/journal.ppat.1000400

**Published:** 2009-04-24

**Authors:** Yogitha N. Srikhanta, Stefanie J. Dowideit, Jennifer L. Edwards, Megan L. Falsetta, Hsing-Ju Wu, Odile B. Harrison, Kate L. Fox, Kate L. Seib, Tina L. Maguire, Andrew H.-J. Wang, Martin C. Maiden, Sean M. Grimmond, Michael A. Apicella, Michael P. Jennings

**Affiliations:** 1 School of Molecular and Microbial Sciences, The University of Queensland, St. Lucia, Brisbane, Queensland, Australia; 2 Center for Microbial Pathogenesis, The Research Institute at Nationwide Children's Hospital and the Department of Pediatrics, Ohio State University, Columbus, Ohio, United States of America; 3 Department of Microbiology and Immunology, University of Iowa, Iowa City, Iowa, United States of America; 4 Core Facilities for Proteomics Research, Institute of Biological Chemistry, Academia Sinica, Taipei, Taiwan; 5 The Peter Medawar Building for Pathogen Research and Department of Zoology, University of Oxford, Oxford, United Kingdom; 6 Institute of Molecular Bioscience, The University of Queensland, St. Lucia, Brisbane, Queensland, Australia; Northwestern University Feinberg School of Medicine, United States of America

## Abstract

Many host-adapted bacterial pathogens contain DNA methyltransferases (*mod* genes) that are subject to phase-variable expression (high-frequency reversible ON/OFF switching of gene expression). In *Haemophilus influenzae*, the random switching of the *modA* gene controls expression of a phase-variable regulon of genes (a “phasevarion”), via differential methylation of the genome in the *modA* ON and OFF states. Phase-variable *mod* genes are also present in *Neisseria meningitidis* and *Neisseria gonorrhoeae*, suggesting that phasevarions may occur in these important human pathogens. Phylogenetic studies on phase-variable *mod* genes associated with type III restriction modification (R-M) systems revealed that these organisms have two distinct *mod* genes—*modA* and *modB*. There are also distinct alleles of *modA* (abundant: *modA11*, *12*, *13*; minor: *modA4*, *15*, *18*) and *modB* (*modB1*, *2*). These alleles differ only in their DNA recognition domain. *ModA11* was only found in *N. meningitidis* and *modA13* only in *N. gonorrhoeae*. The recognition site for the *modA13* methyltransferase in *N. gonorrhoeae* strain FA1090 was identified as 5′-AGAAA-3′. Mutant strains lacking the *modA11*, *12* or *13* genes were made in *N. meningitidis* and *N. gonorrhoeae* and their phenotype analyzed in comparison to a corresponding *mod* ON wild-type strain. Microarray analysis revealed that in all three *modA* alleles multiple genes were either upregulated or downregulated, some of which were virulence-associated. For example, in *N. meningitidis* MC58 (*modA11*), differentially expressed genes included those encoding the candidate vaccine antigens lactoferrin binding proteins A and B. Functional studies using *N. gonorrhoeae* FA1090 and the clinical isolate O1G1370 confirmed that *modA13* ON and OFF strains have distinct phenotypes in antimicrobial resistance, in a primary human cervical epithelial cell model of infection, and in biofilm formation. This study, in conjunction with our previous work in *H. influenzae*, indicates that phasevarions may be a common strategy used by host-adapted bacterial pathogens to randomly switch between “differentiated” cell types.

## Author Summary

The pathogenic *Neisseria* are bacterial pathogens that cause meningitis and gonorrhoea. They have adapted to life exclusively in humans and have developed unique strategies to colonize the host and to evade the immune response. Central among these strategies are genetic switches that randomly turn genes on and off. In most cases, the genes controlled by these switches, contingency genes, are required for making bacterial surface structures. Recently we described a new class of contingency gene that methylates DNA. Rather than affecting the synthesis of a single surface structure, on/off switching of this DNA-methyltransferase gene leads to random switching of multiple genes. In this study, we have shown that this mechanism exists in all pathogenic *Neisseria*, and alters expression of multiple genes in all cases we examined. The two distinct populations of bacteria generated by this process had different behavior in model systems of colonization and infection. Understanding this process is key to understanding these human pathogens, and to developing strategies for treatment and prevention of the diseases they cause.

## Introduction

The pathogenic *Neisseria* are host-adapted human pathogens that pose a significant health problem worldwide. *Neisseria meningitidis* colonizes the upper respiratory tract and causes meningitis and septicemia. *Neisseria gonorrhoeae* colonizes the genitourinary tract and can cause a spectrum of disease ranging from uncomplicated mucosal infection to disseminated gonococcal infection. There is no *N. gonorrhoeae* vaccine, and no fully protective vaccine for *N. meningitidis*. Vaccine development has been hampered due to the high frequency of antigenic and phase variation of surface structures typical of these organisms.

Phase variation is the high frequency reversible on/off switching of gene expression and is commonly mediated by mutations in simple tandem DNA repeats in the open reading frame or promoter region of genes encoding surface expressed virulence determinants [1]. The independent, random switching of these genes results in phenotypically diverse populations that enables rapid adaptation to host environments and evasion of immune responses [2]. While phase variation is typically associated with genes encoding surface structures, several host-adapted bacterial pathogens have methyltransferases (*mod* genes) associated with type III restriction modification (R-M) systems that contain simple tandem DNA repeats that have been proven to phase vary (*Pasteurella haemolytica* [3], *Haemophilus influenzae* [4] and *Helicobacter pylori* [5]) or predicted to phase vary (*N. meningitidis*, *N. gonorrhoeae* [6],[7], and *Moraxella catarrhalis* [7]), as reviewed in Fox *et al* [8].

R-M systems are ubiquitous in bacteria and confer protection to the bacterial host against invasion by foreign DNA [9]. R-M systems are classified into three groups; Types I, II or III on the basis of subunit composition, DNA cleavage position, sequence-specificity and co-factor requirements [10]. Type III systems are composed of a methyltransferase (modification, *mod*) gene and an endonuclease (restriction, *res*) gene, whose products form a two-subunit enzyme – Mod and Res [11]. Type III systems are unusual in that Res must form a complex with Mod to be functional [12], however, Mod can function independently of Res [13]. The Mod subunit contains several conserved motifs in the N- and C-terminal regions and the central region contains the DNA-recognition domain that dictates sequence specificity [14].

In *H. influenzae*, the random switching of the *modA* gene controls expression of a phase variable regulon of genes (a “phasevarion”), via differential methylation of the genome in the *modA* ON and OFF states [15]. This was the first report of the coordinated random switching of a “regulon” of genes and, considering the wide distribution of phase variable type III R-M systems, may represent a widely used mechanism in bacterial pathogens [8]. In this study we investigate the phase variable type III R-M systems of pathogenic *Neisseria* to determine whether they play a role in gene regulation and virulence.

## Results

### Multiple phase-variable type III R-M systems in pathogenic *Neisseria*

**Figure 1:**
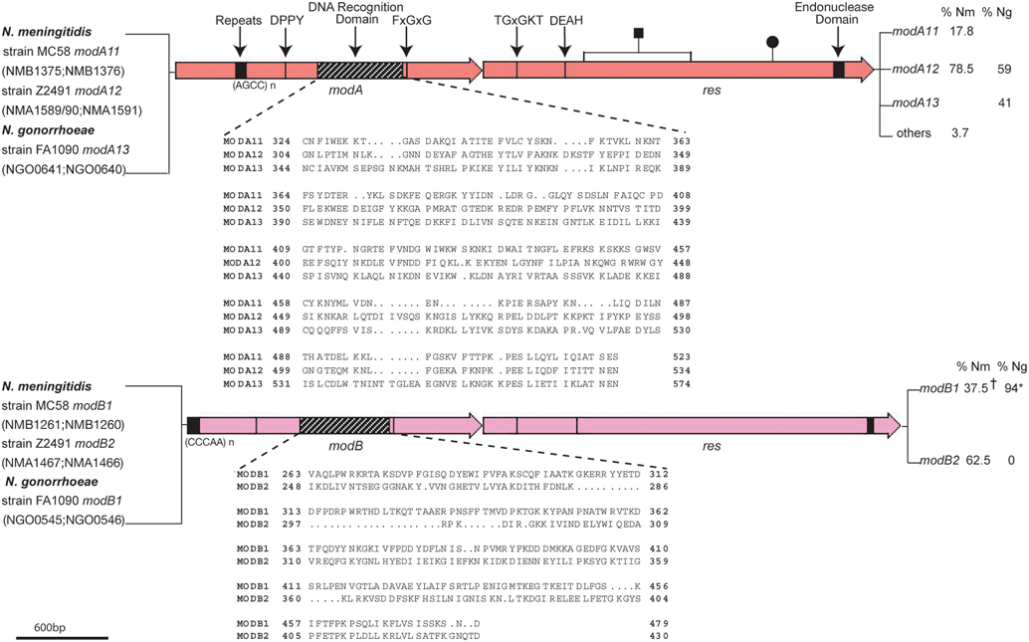
Diagrammatical representation of the *mod* genes of *N. meningitidis* and *N. gonorrhoeae*. The methylase (*mod*) genes, restriction endonuclease (*res*) genes, and repeat regions that mediate phase variation are indicated. Also shown are the conserved, characteristic motifs found within type III R-M systems, which include in *mod*: the catalytic region (DPPY) and the AdoMet (methyl donor) binding pocket (FXGXG) [66],[67], and in *res*: the ATP binding motif (TGxGKT) the motif linked to ATP hydrolysis (DEAH), and the endonuclease domain [68]–[70]. The *mod* and *res* genes are colored to indicate differences in homology between both *mod* genes and both *res* genes, respectively. A variable region within *mod* (highlighted in stripes) contains the DNA recognition domain [14]. The percent distribution of the *mod* alleles in a *N. meningitidis* serogroup B collection and a *N. gonorrhoeae* clinical isolate collection is shown to the right of each gene. Strains and accession numbers that define the *mod* alleles are shown to the left. n indicates the number of repeats (refer to Table S1 and Table S2 for exact repeat numbers). A black circle on a line and black square on a line indicate the position of a frame shift mutation and large deletion, respectively (Table S1 and Table S2). ^†^, some *modB1* strains contain a premature stop codon after the DPP motif (Table S1). *, one *N. gonorrhoeae* strain does not have the *modB* gene. Others, minor/infrequent alleles.

To investigate whether the type III R-M systems of the pathogenic *Neisseria* behave as a phasevarion [15], we first carried out a phylogenetic analysis of *mod* genes associated with type III R-M systems of *N. meningitidis* and *N. gonorrhoeae*. A comparison of the available genome sequences revealed that each strain contains two distinct phase variable *mod* genes, which we define as *modA* and *modB*, that share only 37% similarity to each other along the full length of the Mod deduced amino acid sequence. Both genes contain tracts of simple tandem repeats, 5′-AGCC-3′ (*modA*) and 5′-CCCAA-3′ (*modB*), that mediate phase variation of *mod* gene expression (Figure 1). *ModA* is highly homologous (>90% identity along the length of the Mod deduced amino acid sequence excluding the variable DNA recognition domain) to the *mod* gene of *H. influenzae* strain Rd (HI1058/56) [4],[15]. Differences in the *modA* DNA recognition domain [14] (Figure 1) have previously been observed in *H. influenzae* [16] with 17 distinct *mod* alleles defined in this organism (*modA1–17*; [17]). The *Neisseria modA* alleles present in the genome strains surveyed have the designations *modA11*, *12* and *13* and share >94% similarity to each other along the length of the Mod deduced amino acid sequence, excluding the variable DNA recognition domain. Our recent work also shows that the *modA* gene of *H. influenzae* and *Neisseria* are essentially the same gene with evidence of horizontal transfer of this gene in both directions between these organisms [17]. Unlike *modA*, the *modB* gene appears to be specific to *Neisseria* species. Two distinct *modB* alleles, *modB1* and *2*, distinguished by differences in their DNA recognition domain, were also observed (Figure 1). *ModB1* and *2* share >95% similarity to each other along the length of the Mod deduced amino acid sequence, excluding the variable DNA recognition domain (which shares <33% identity).

To investigate whether additional alleles of *modA* and *modB* are present in these organisms, and to look at the distribution of *mod* alleles and their repeat sequence type and number, sequence analysis of a large, genetically diverse set of *N. meningitidis* and *N. gonorrhoeae* isolates was performed. This analysis revealed that all strains examined contained both *modA* and *modB* genes. Sequencing of the repeat region of the *mod* alleles revealed that the repeat numbers vary in length between different strains, resulting in the *mod* genes being in-frame (ON) or out-of-frame (OFF) for expression, consistent with phase variation of the *mod* genes (Table S1, Table S2). The *N. gonorrhoeae* strains contained either the *modA12* or *modA13* allele, and only the *modB1* allele. One strain was found not to have a *modB* gene (Figure 1, Table S2).

**Figure 2:**
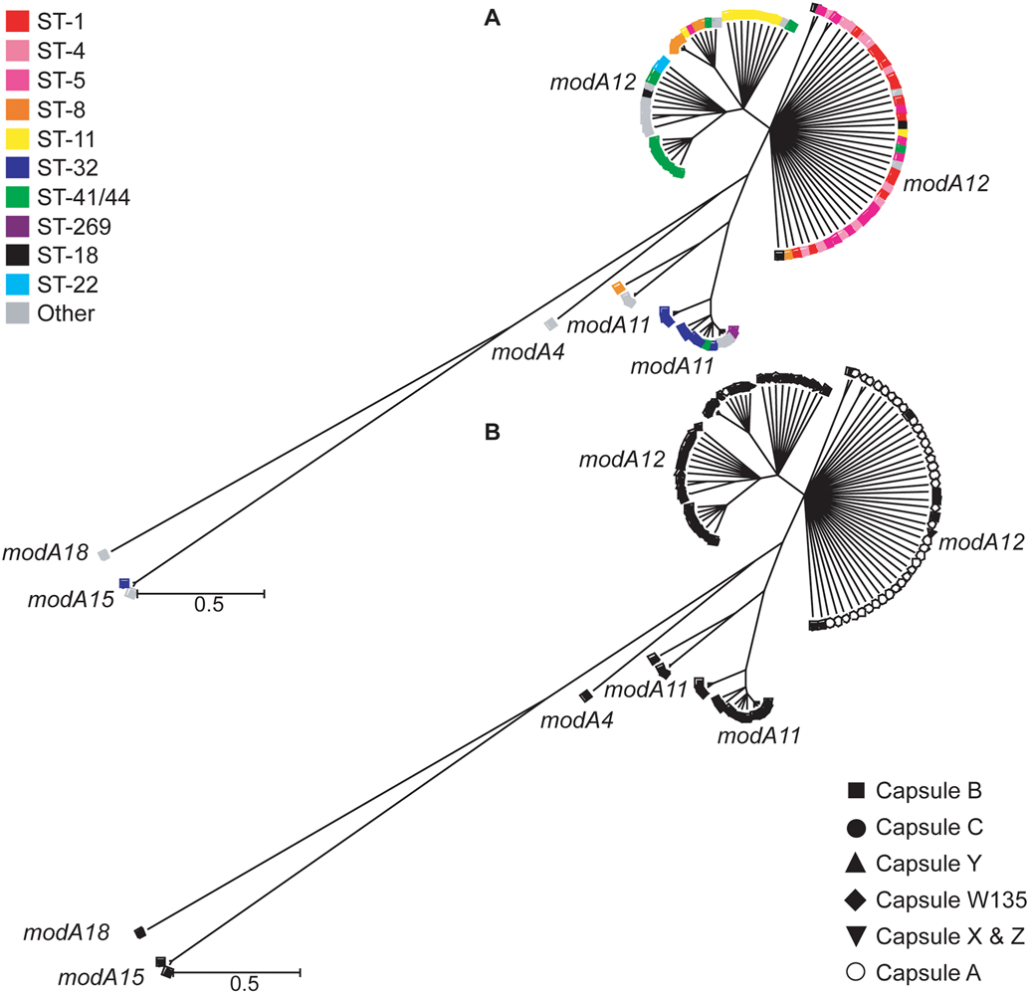
Phylogenetic tree inferred from aligned *modA* genes belonging to a collection of 107 *N. meningitidis* isolates. More than 500 trees were generated using Clonalframe from which a 95% majority-rule consensus tree was derived and imported into MEGA version 4.0 for further interpretation. (A) Each *modA* gene was annotated according to clonal complex. (B) Each *modA* gene was annotated according to the serogroup of the corresponding isolate distinguishing *modA12* genes belonging to capsule A meningococci.

A complete analysis of *modA* allele distribution was conducted in *N. meningitidis*, which has a well characterized population structure defined by multi locus sequence typing (MLST; [18]). The complete 107 strain MLST *modA* survey revealed that the majority of *N. meningitidis* strains had either the *modA11* or *modA12* allele, with *modA15* found in two strains and *modA4* and *modA18* found in one isolate each (Figure 1, Figure 2A and 2B, Table S1). The most notable associations were in capsule type, where 100% of serogroup A strains and 92% of serogroup C strains contained the *modA12* allele (Table 1, Figure 2B). Some association with clonal complex was also observed, with meningococci belonging to the ST-32 clonal complex predominantly harbouring the *modA11* allele. Further clustering could be seen among ST-41/44 and ST-8 clonal complexes. Unlike *N. gonorrhoeae*, which contained only one type of *modB* allele, *modB1*, *N. meningitidis* strains contained either *modB1 or modB2*. There were seven strains, all from the ST-32 group, which contained point mutations in *modB1* suggesting the gene is inactive in these strains (Figure 1, Table S1).

**Table 1:** Distribution of *modA* alleles in the *N. meningitidis* MLST strain collection by serogroup.

	Serogroup					
	A	B	C	Y	W	Other
***modA4***	0	1 (2%)	0	0	0	0
***modA11***	0	16 (33%)	1 (8%)	0	0	0
***modA12***	37 (100%)	29 (59%)	11 (92%)	2 (100%)	1 (100%)	4 (100%)
***modA15***	0	2 (4%)	0	0	0	0
***modA18***	0	1 (2%)	0	0	0	0

### *ModA* expression and phase variation

**Figure 3:**
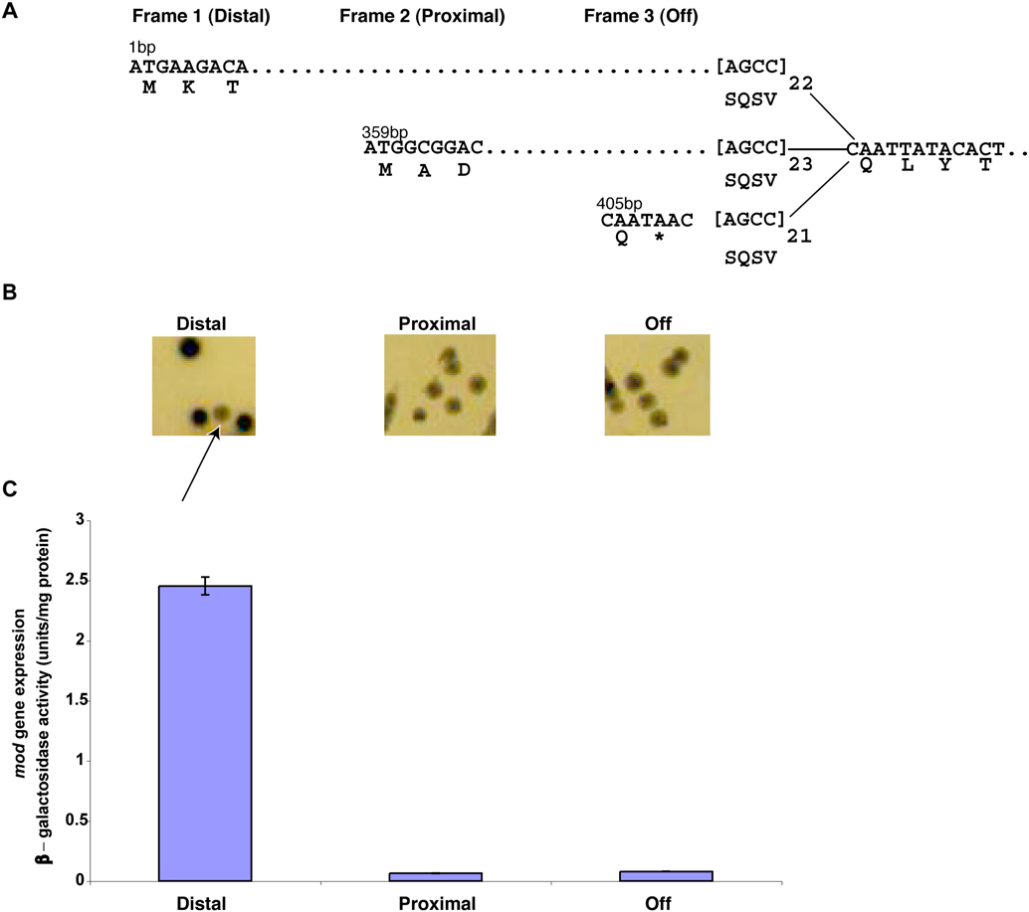
Differences in Mod expression from alternate initiation codons. A chromosomally located *modA::lacZ* reporter fusion in *N. meningitidis* strain MC58 was used to determine expression from all three possible reading frames generated by different repeat numbers. (A) Schematic diagram showing that translation of the *mod* gene could be initiated from one of three frames (Distal, Proximal, or Off) depending on the number of 5′-AGCC-3′ repeats. (B) Phenotypic differences of colonies from each reading frame as observed on brain–heart infusion (BHI) S-gal plates. The arrow shows a phase variant colony that has switched from Distal to OFF. (C) β-galactosidase assay showing quantitative differences in the level of *mod* gene expression between Distal, Proximal, and Off. A unit is defined as mg of O-nitrophenyl hydrolyzed per min–1. A Student's *t*-test confirmed a significant difference between expression of Distal and Proximal (p = 0.0024). A small difference was observed between Proximal and OFF (p = 0.0146), but this can be accounted for by phase variation in the population of the *modA11* gene from OFF to ON.

The *modA* genes of *N. meningitidis* and *N. gonorrhoeae* have two alternate initiation codons (Distal ATG and Proximal ATG) that are predicted to code for proteins of either 589 aa/640 aa or 707 aa/758 aa, depending on the number of tetranucleotide repeats that are present (Figure 3A). As this study is focused on *modA* phase variation and expression, a clear understanding of the relationship between tetranucleotide repeat number and *modA* expression was established. *ModA* expression was examined in the three frames; Distal, Proximal and OFF (which has no candidate ATG and a stop codon immediately after the 5′-AGCC-3′ repeats), by constructing a *modA::lacZ* reporter fusion in the *N. meningitidis* strain MC58 chromosome (Figure 3A and Figure S1). Maximal expression was found to be from the Distal ATG only and unlike *H. influenzae* strain Rd [15], minimal expression was observed from the Proximal ATG and the OFF frame (Figure 3B and 3C).

Natural *modA* ON and OFF colonies of *N. meningitidis* strain MC58 and *N. gonorrhoeae* strain FA1090 were required for microarray analysis and biological characterization experiments. *N. meningitidis* strain MC58 has 21 5′-AGCC-3′ repeats resulting in the *modA11* gene being out-of frame (OFF) from the Distal ATG. Single colonies of MC58 were picked and screened by PCR and sequencing to find *modA11* in-frame (ON) with the Distal ATG (see Figure 3). Similarly, single colonies of *N. gonorrhoeae* strain FA1090 were picked and screened by PCR and sequencing to find *modA13* in-frame and also out-of-frame with the Distal ATG. During this process, *ModA13* repeat tracts ranging from 13 (ON) to 26 (OFF) and also 37 (ON) were observed, demonstrating phase variation of *N. gonorrhoeae* strain FA1090 *modA13* (results not shown).

### Analysis of differentially expressed genes in *N. meningitidis modA11* and *modA12* phasevarions

**Figure 4:**
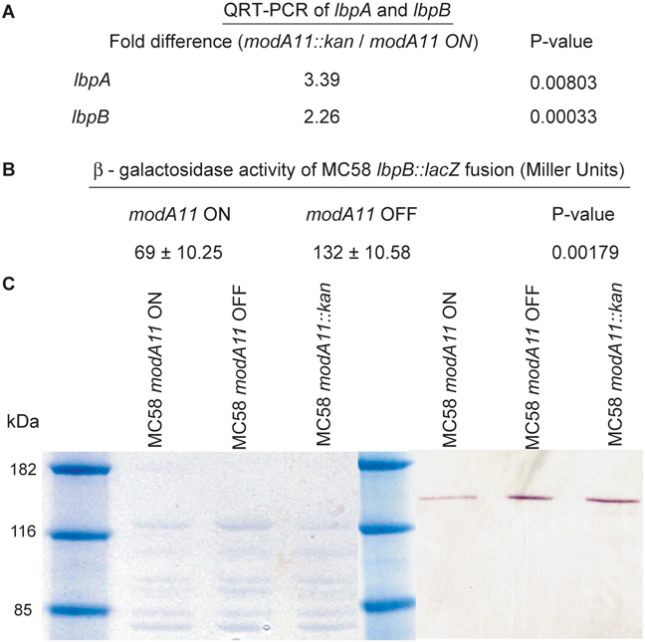
Analysis of wild-type MC58 *modA11* ON, MC58 *modA11* OFF, and MC58 *modA11::kan* for LbpA and LbpB expression. (A) Quantitative RT-PCR of *lbpA* and *lpbB* expression. Relative gene expression of *lbpA* and *lbpB* is higher in the MC58 *modA11::kan* mutant compared to wild-type MC58 *modA11* ON. (B) Effect of *modA11* phase variation on expression of the *lbpB* gene. β-galactosidase assays showed a statistically significant difference in the level of *lbpB::lacZ* gene expression resulting from *modA11* repeat tract changes. A 1.9-fold difference in expression was observed between *modA11* ON and *modA11* OFF. P-values were calculated using a Student's *t*-test (C). The LbpA specific monoclonal antibody 296-H1 was used to assess expression of LbpA. The positions of molecular weight standard proteins are shown on the right in kilo Daltons (kDa). The left panel shows coomasie-stained wild-type MC58 *modA11* ON, phase-variant MC58 *modA11* OFF, and MC58 *modA11::kan* whole cells to show equal loadings of cell extracts. The right panel shows the Western blot of wild-type MC58 *modA11* ON, phase-variant MC58 *modA11* OFF, and MC58 *modA11::kan* whole cells probed with a monoclonal LbpA specific antibody.

Having established the relationship between *modA* repeats and *modA* expression (see Figure 3), we were in a position to conduct studies to determine whether phase variation of the various *modA* alleles in pathogenic *Neisseria* resulted in changes in gene expression. These studies were initiated with *N. meningitidis* strain MC58 *modA11* gene, where the *modA11* gene was inactivated by insertion of a *kan^r^* cassette to make the mutant strain MC58 *modA11::kan* (Figure S2). Wild-type MC58 *modA11* ON and MC58 *modA11::kan* were compared by microarray analysis using *N. gonorrhoeae/meningitidis* genome arrays (Materials and Methods). Initially, microarray analysis was performed using RNA isolated from wild-type MC58 *modA11* ON and MC58 *modA11::kan* strains grown under standard culture conditions. However, these studies revealed no statistically significant difference in gene expression. Experiments were then performed in which *N. meningitidis* were cultured under iron-limiting conditions to more closely reflect *in vivo* conditions. Using this more physiologically relevant culture condition, many genes were found to have an expression ratio of 1.5-fold and over, with 162 genes up-regulated in MC58 *modA11::kan* relative to wild-type and 123 genes down-regulated, confirming *modA11* phase variation has an influence on gene expression (Table 2, Table S3). Five of these genes encode surface exposed proteins, including NMB1540 (*lbpA*) and NMB1541 (*lbpB*), encoding LbpA and LbpB respectively, which are part of the lactoferrin receptor that allows acquisition and binding of iron from lactoferrin containing compounds. LbpA is the TonB-dependent integral outer membrane lactoferrin receptor and iron transport channel. LbpB is an accessory lipoprotein anchored to the outer membrane that contributes to lactoferrin binding/use [19],[20]. The lactoferrin receptor is a potential vaccine candidate in *N. meningitidis* [21]. Quantitative real time PCR (RT-PCR) was performed to confirm that the lbpA and *lbpB* genes were expressed at a higher level in MC58 *modA11::kan* compared to the MC58 *modA11* ON parent strain (see Figure 4A). Altered expression was further confirmed by an lbpB::lacZ fusion (Figure S3) located on the chromosome of each strain, which showed ∼2-fold higher expression in the *modA11::kan* mutant strain compared to the *modA11* ON parent strain (see Figure 4B). Consistent with this, western blot analysis confirmed the effect of *modA11* phase variation on expression of LbpA, with an apparent reduction in expression when *modA11* is ON (Figure 4C). The same effect is seen when comparing a wild-type *modA11* ON strain to either a *modA11::kan* mutant or a natural phase variant in which the *modA11* gene had switched OFF due to an alteration in the 5′-AGCC-3′ repeat tract (from 22 to 21 AGCC repeats; see Figure 4C), confirming the regulation of LbpA by ModA11 is not related to the use of a *kan^r^* inactivated *modA11* gene.

**Table 2:** A selection of differentially expressed genes from microarray expression studies.

	Gene ID	Description	Ratio	QRT-PCR	B-Stat
**Reduced expression in ** ***N. meningitidis modA11*** ** mutant**	NMB0144	50S ribosomal protein L23	0.40	0.58±0.16	4.99
	NMB0144	30S ribosomal protein S3	0.40	0.40±0.06	5.44
	NMB0148	30S ribosomal protein S14	0.46	0.57±0.08	3.41
**Increased expression in ** ***N. meningitidis modA11*** ** mutant**	NMB0014	3-deoxy-D-manno-octulosonic-acid transferase	2.97	2.46±0.50	5.89
	NMB1540	Lactoferrin-binding protein A	2.33	3.39±0.47	5.51*
	NMB1541	Lactoferrin-binding protein B	2.22	2.26±0.63	4.10†
	NMB1898	Lipoprotein	2.48	2.24±0.58	4.32
**Reduced expression in ** ***N. meningitidis modA12*** ** mutant**	NMA1581	Membrane lipoprotein	0.72	0.65±0.14	4.35
**Increased expression in ** ***N. meningitidis modA12*** ** mutant**	NMB0950	Succinate dehydrogenase, flavoprotein subunit	1.67		5.21
	NMB0951	Succinate dehydrogenase, iron-sulfur protein	1.83	2.61±0.46	2.90
	NMB1206	Bacterioferritin B	1.42	2.14±0.35	2.48
	NMB1403	FrpA-C-related protein	1.69		5.11
	NMB1405	FrpA-C-related protein	1.73	2.21±0.49	4.25
**Reduced expression in ** ***N. gonorrhoeae modA12*** ** mutant**	NGO2090	Putative ABC transporter, permease protein, enterobactin	0.44		1.72
	NGO2092	Ferric enterobactin periplasmic binding protein	0.62		0.61
	NGO2093	FetA	0.24	2.50±0.056	2.51
**Increased expression in ** ***N. gonorrhoeae modA12*** ** mutant**	NGO0365	Site-specific DNA-methyltransferase M.NgoVII	1.78		0.74
	NGO0364	Restriction endonuclease R.NgoVII	1.60		0.33
	NGO0861	Hypothetical protein	2.43		2.01
	NGO0860	Hypothetical protein	2.03		1.64
**Reduced expression in ** ***N. gonorrhoeae modA13*** ** mutant**	NGO1581	Phosphate permease, putative	0.29	0.208±0.05§	8.53
	NGO1931	Glyceraldehyde-3-phosphate dehydrogenase, typeI	0.37	0.415±0.06	5.10
	NGO2066	Pilin silent gene cassette	0.42		5.11
	NGO0554	Hypothetical protein	0.49	0.228±0.04	2.56
**Increased expression in ** ***N. gonorrhoeae modA13*** ** mutant**	NGO0318	DNA repair protein	1.98		2.02
	NGO1368	Antibiotic resistance efflux pump component	2.20	13.43±0.763§	5.51
	NGO0340	Cysteine synthase	2.23		4.20
	NGO0372	Amino acid ABC transporter, periplasmic binding protein	2.27	5.30±1.18	3.28
	NGO0373	Amino acid ABC transporter, permease protein	3.01	7.24±0.588	5.47
	NGO0374	Amino acid ABC transporter, ATP-binding protein	2.81	3.83±0.616	7.14
	NGO0656	oxalate/formate antiporter	2.39	3.04±0.708	6.04
	NGO0655	Exodeoxyribonuclease VII, large subunit	3.16		5.91
	NGO0650	ATP-dependent RNA helicase,	3.14		6.62
	NGO0198	Ammonium transporter	3.18	5.44±0.630	6.45
	NGO0927	Neisseria specific protein conserved hypothetical protein	2.93	6.19±0.629	2.46
	NGO0928	5-methyltetrahydropteroyltriglutamate	3.01	10.89±0.493	6.59
	NGO0929	5,10-methylenetetrahydrofolate reductase	4.92	20.16±0.410§	8.32

The genes listed are either downregulated or upregulated in the *N. meningitidis* MC58 *modA11::kan* mutant, *N. meningitidis* B6616/77 *modA12::kan* mutant, *N. gonorrhoeae* 96D551 *modA12::kan* mutant, or *N. gonorrhoeae* FA1090 *modA13::kan* mutant (refer to Table S3, Table S4, Table S5, and Table S6 for a complete list of downregulated or upregulated genes in the *N. meningitidis modA11* and *modA12* mutants and *N. gonorrhoeae modA12* and *modA13* mutants). The identity of the gene is indicated with the gene ID in the annotation of the *N. meningitidis* strain MC58 genome, *N. meningitidis* strain Z2491 genome, or *N. gonorrhoeae* strain FA1090 genome (TIGR). The average ratio presented is the mean of *N. meningitidis* MC58 *modA11::kan* mutant: wild-type MC58 *modA11* ON and *N. meningitidis* B6116/77 *modA12::kan* mutant: wild-type B6116/77 *modA12* ON, or the mean of *N. gonorrhoeae* 96D551 *modA12::kan* mutant: wild-type 96D551 *modA12* ON and *N. gonorrhoeae* FA1090 *modA13::kan* mutant: wild-type FA1090 *modA13* ON, from six replicate spots on three independent microarrays, incorporating a dye swap. Only those genes with an expression ratio above 1.5-fold were included in this study.

*: Confirmed by Western blot (Figure 4C).

**†:** Confirmed by β-galactosidase assay (see Figure S3 and Figure 4B).

**§:** Quantitative RT-PCR (qRT-PCR) was also done with *N. gonorrhoeae* strain O1G1370 wild-type *modA13* ON and O1G1370 *modA13::kan* mutant. Results are as follows: NGO1581; 0.524±0.101, *mtrF*; 4.59±0.264, *metF*; 3.51±0.805. Gene expression confirmed by qRT-PCR in a natural FA1090 *modA13* ON and FA1090 *modA13* OFF strain. Results are as follows: NGO1581; 0.090±0.101, *mtrF*; 17.11±0.956, *metF*; 3.51±0.477.

A similar microarray study was conducted using a *N. meningitidis modA12* clinical isolate, B6116/77. To determine whether phase variable expression of the *N. meningitidis* strain B6116/77 *modA12* gene also led to alteration in global gene expression, the *modA12* gene was inactivated by insertion of a *kan^r^* cassette to make the mutant strain B6116/77 *modA12::kan* (Figure S2). Wild-type B6116/77 *modA12* ON and B6116/77 *modA12::kan* were compared by microarray analysis using *N. gonorrhoeae/meningitidis* genome arrays. Experiments were performed under the same conditions as described above. Twenty six genes were found to have an expression ratio of 1.5-fold and over, with 14 genes up-regulated in B6116/77*modA12::kan* relative to wild-type and 12 genes down-regulated, confirming *modA12* phase variation has an influence on gene expression (Table 2, Table S4). The set of genes differentially expressed in the *modA12* mutant were different to the *modA11* set of genes. This is consistent with the differences in the DNA recognition domain between *modA11* and *modA12*, and confirms that these distinct alleles control different phasevarions in *N. meningitidis*.

### Analysis of differentially expressed genes in *N. gonorrhoeae modA13*

An additional phasevarion study was conducted in *N. gonorrhoeae*. In this case, a *modA13* knockout mutant was constructed by interrupting the *modA13* gene with a *kan^r^* cassette (Figure S2). Comparison of the phenotype of the FA1090 *modA13::kan* mutant strain with wild-type FA1090 *modA13* ON formed the basis of expression and phenotypic studies.

Global gene expression was compared between wild-type FA1090 *modA13* ON and FA1090 *modA13::kan* under iron-limiting conditions. 34 genes were up-regulated in FA1090 *modA13::kan* relative to wild-type, and 20 genes were down-regulated (Table 2, Table S5). Five of the differentially regulated genes have obvious roles in virulence; four in oxidative stress and one in antimicrobial resistance. NGO0929 (*metF*) and NGO0928 (*metE*) are part of the MetFE operon, which plays a role in the methylation of homocysteine, the final step of methionine biosynthesis, and is involved in defence against oxidative stress [22]. NGO0554 encodes a gonococcal-specific hypothetical protein that is shown to protect against damage caused by high levels of H_2_O_2_ [23]. NGO0650 (*recN*) encodes the DNA repair protein RecN. The gonococcal RecN protein is demonstrated to be involved in DNA repair and DNA transformation [24] and plays an important role in H_2_O_2_ damage protection as well as resistance to killing by polymorphonuclear leukocytes [25]. NGO1368 (*mtrF*) encodes the inner membrane protein, MtrF, and has been shown to have a role in antimicrobial resistance [26].

Our phylogenetic analysis revealed that *N. gonorrhoeae* strains have one of two distinct *modA* alleles (*modA12* or *13*; see Figure 1), indicating that different phasevarions may exist within *N. gonorrhoeae* and that strains with the same *mod* allele may regulate similar sets of genes. To determine if a strain with the same DNA recognition domain as FA1090 (*modA13* allele) would result in the same set of genes being regulated, we chose a *N. gonorrhoeae* clinical isolate, strain O1G1370, from a representative set of *N. gonorrhoeae* strains (Table S2) that also contains a *modA13* allele. A *modA13::kan* knockout mutant was made using the same approach as described for FA1090 (Materials and Methods). Quantitative RT-PCR on the *metF*, *mtrF* and NGO1581 genes confirmed that *metF* and *mtrF*, which are up-regulated in expression in the FA1090 *modA13::kan* mutant, are also up-regulated in the O1G1370 *modA13::kan* mutant (Table 2). NGO1581, which was down-regulated in the FA1090 *modA13::kan* mutant, is also down-regulated in the O1G1370 *modA13::kan* mutant (Table 2). Furthermore, similar results were seen with quantitative RT-PCR on the same set of genes when comparing a wild-type FA1090 *modA13* ON strain to a natural phase variant of FA1090 in which the FA1090 *modA13* gene had switched OFF due to an alteration in the 5′-AGCC-3′ repeat tract (from 37 to 26 AGCC repeats) (Table 2), confirming the regulation of this set of genes by ModA13 is not related to the use of a *kan^r^* inactivated *modA13* allele.

When we conducted microarray analysis of a *N. gonorrhoeae modA12* strain 96D551, comparison of 96D551 *modA12* ON and 96D551 *modA12::kan* (OFF) strains revealed a distinct set of genes being regulated compared to the *modA13* data above (Table 1, Table S6). These results are consistent with the differences in the DNA recognition domain between *modA13* and *modA12* (see below), and supports the idea that these distinct alleles control different phasevarions in *N. gonorrhoeae*. However, unlike the *modA11*, *modA13* and *modA12* (*N. meningitidis*) expression studies described above, the 96D551 *modA12* ON and 96D551 *modA12::kan* (OFF) strains showed a significant difference in growth rate for the cultures used to make RNA (Figure S4D). We cannot rule out the possibility that these differential growth rates may have influenced the gene expression data in Table 1 and Table S6.

### *N. gonorrhoeae* strain FA1090 ModA13 recognition site is 5′-AGAAA-3′

**Figure 5:**
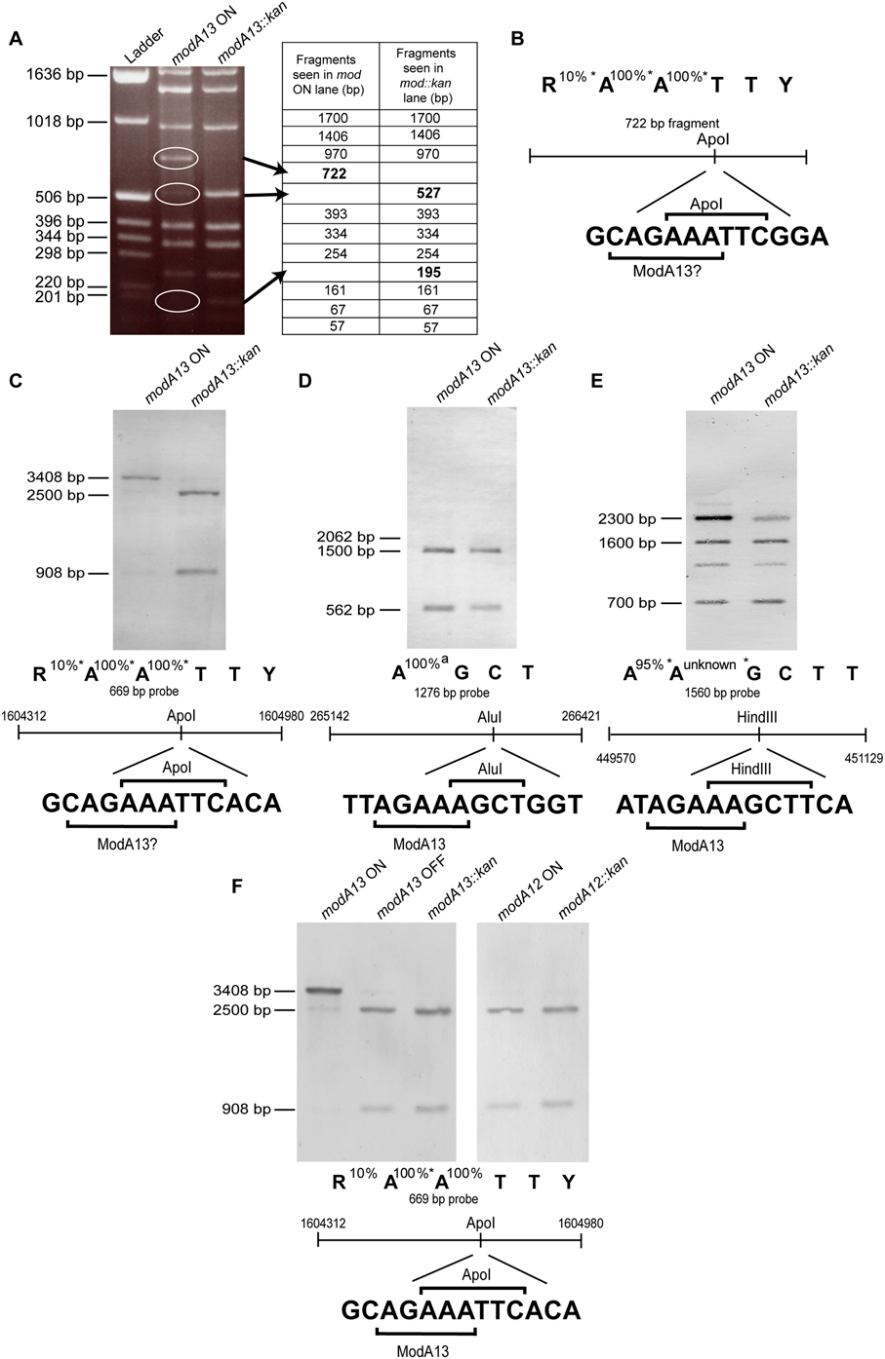
Identification of the ModA13 recognition methylation target sequence. (A) *Apo*I restriction digest of plasmid pCmGFP isolated from FA1090 *modA13* ON and FA1090 *modA13::kan* cells. The *modA13* ON lane shows the presence of a 722-bp fragment that results from lack of restriction at a single *Apo*I restriction site. In the *modA13::kan* lane, this fragment is cut into fragments of 527 and 195 bp. (B) The *Apo*I recognition sequence showing percentage inhibition of restriction by methylation of each adenosine as indicated by REBASE [27], and a schematic diagram of the 722 bp pair fragment showing the *Apo*I recognition site, overlapping with a putative ModA13 recognition site. The central panels show Southern blots of chromosomal DNA extracted from *modA13* ON and *modA13::kan* FA1090 cells. (C) DNA digested with *Apo*I and probed with a PCR product containing an *ApoI*/AGAAA overlap showed inhibition of digestion in the *modA13* ON lane compared to the *modA13::kan* lane. (D) DNA digested with *Alu*I and probed with a PCR product containing an *Alu*I/AGAAA overlap showing no difference in restriction between the *modA13* methylated and unmethylated chromosomes. (E) DNA digested with *Rsa*I and *Hind*III, and probed with a PCR product containing a *Hind*III/AGAAA overlap, showed restriction is inhibited in the *modA13* ON lane as compared to the *modA13::kan* lane. Below each blot is the recognition site for each of the restriction enzymes used, and their known sensitivities to adenosine methylation as supplied by REBASE in the case of *Apo*I and *Hind*III [27]. Schematics of the probes used in each blot include the coordinates of the FA1090 genome to which the primers bind (see Table S7) and the overlap of the restriction enzyme recognition sequence with that of ModA13. (F) Chromosomal DNA extracted from *N. gonorrhoeae* strains FA1090 *modA13* ON, *modA13* OFF, *modA13::kan*, and 96D551 *modA12* ON and *modA12::kan* cells, digested with *Apo*I and probed as in (C).

In all cases, phase variation or mutagenesis of *modA* of pathogenic *Neisseria* results in altered gene expression, defining these systems as functional phasevarions. In order to determine whether the observed changes in *modA* expression correspond to global changes in DNA methylation, thereby indicating this as the likely mechanism of gene control, it was necessary to identify one or more of the *modA* target sites. In addition to confirming global changes in methylation, target site identification would also facilitate future studies on the molecular mechanisms operating at individual promoters within the phasevarion. In order to identify methylation target sites, a strategy based on inhibition of DNA restriction was used. In initial studies, plasmid pCmGFP was isolated from *N. gonorrhoeae* or *N. meningitidis modA11*, *modA12 or modA13* ON strains and their corresponding *modA::kan* mutants, and digested with a range of restriction enzymes known to be inhibited by methylation of an adenine within their recognition sequence (see Materials and Methods). Differences in digestion patterns between plasmid extracted from *modA* ON cells (ModA methylated DNA) and *modA::kan* cells (DNA not methylated by ModA) would indicate an overlap of the respective ModA methylated target and the restriction enzyme used. No such fortuitous inhibition pairs were seen with *modA11* or *modA12* strains, but were with *modA13*. Figure 5A shows an obvious difference in the restriction pattern of plasmid extracted from *modA13* ON and *modA13::kan* cells, indicating overlap between the ModA13 site and *Apo*I. The recognition sequence of *Apo*I is 5′-RAATTY-3′. The specific *Apo*I site displaying inhibition (5′-AAATTC-3′) was not unique on the plasmid. Comparison between the inhibited *Apo*I site and other *Apo*I sites whose sequence was also 5′-AAATTC-3′ revealed that the overlap with ModA13 must be 5′ to the *Apo*I site. As seen in Figure 5B, methylation of the different adenines in the *Apo*I recognition site inhibits digestion to varying degree. Since over-digestion did not result in the 722 bp band being cleaved into the two smaller fragments, it can be assumed that the first adenine of the *Apo*I sequence was not the methylation target, as methylation of this adenine would result in only 10% inhibition of restriction [27], an effect which could potentially be overcome by over-digestion. Therefore, depending on which of the other two adenines was methylated, the ModA13 recognition sequence must be found within 5′-CAGAAA-3′.

To confirm which adenine is the ModA13 methylation target, and to further specify the ModA13 recognition sequence, overlaps of the putative ModA13 recognition sequence and *Apo*I restriction sites were identified on the FA1090 chromosome. Chromosomal DNA was extracted from FA1090 *modA1*3 ON and FA1090 *modA13::kan* cells, digested with *Apo*I and examined by Southern blot. Inhibition of *Apo*I restriction at the internal *Apo*I/ModA13 overlap results in the presence of one large band at 3408 bp, while the unmethylated DNA (*modA13::kan*) is cleaved at this site by *Apo*I into two smaller bands of 2500 and 908 bp (Figure 5C). This confirms that *Apo*I restriction can be inhibited in DNA methylated by ModA13, as shown by the plasmid digest (Figure 5A). Similar studies were done with three other *Apo*I restriction sites in the genome that overlap ModA13 with 5′-TAGAAA-3′, 5′-GAGAAA-3′ or 5′-AAGAAA-3′. In each of these cases, *Apo*I restriction of ModA13 methylated DNA was inhibited compared with DNA extracted from *modA13::kan* cells (data not shown). This confirms the ModA13 recognition sequence to be 5′-AGAAA-3′.

To identify which of the two potential adenines in 5′-AGAAA-3′ is methylated, ModA13 recognition sequences in the FA1090 chromosome were identified which overlapped with restriction enzymes other than *Apo*I and were known to be inhibited by methylation of adenines. Two of these enzymes were *Hin*dIII (5′-AAGCTT-3′) and *Alu*I (5′-AGCT-3′). Although the recognition sequence of *Hin*dIII contains two adenines (Figure 5E), both these adenines are part of the ModA13 recognition sequence. Methylation of the adenine in the *Alu*I recognition sequence is known to result in complete inhibition [28]. When chromosomal DNA digested with *Alu*I is probed using a PCR product containing an *Alu*I/ModA13 overlap (Figure 5D) no difference in restriction is seen between the *modA13* ON and *modA13::kan* lanes, indicating that the common adenine in this overlap is not methylated by ModA13. This suggests that ModA13 methylates AGAAA on the second most 3′ adenine of recognition site 5′-AGAAA-3. Information on the sensitivity of *Hin*dIII to hemimethylation is only known for the 5′ adenine of the *Hin*dIII recognition site. Hemimethylation of this adenine results in a 95% inhibition of restriction [27]. Using a random site in the FA1090 genome where the overlap between the ModA13 target site and *Hin*dIII resulted in the 5′ adenine of *Hin*dIII site corresponding to the second last adenine of the AGAAA (see Figure 5E) we were able to determine whether this was the residue methylated by ModA13. The results shown in Figure 5E confirm the expected restriction inhibition phenotype [27] allowing us to conclude that the ModA13 methylation site is AGAA^m^A, with the methyl group being added to the third adenine in the sequence.

Having established the ModA13 target sequence, we tested DNA derived from a *modA12* strain and confirmed that the ModA12 target site was distinct as there is no difference between *modA12* ON and *modA12::kan* (OFF) DNA in a ModA13/*Apo*I inhibition assay (Figure 5F). Analysis of the FA1090 genome has revealed a total of 5135 ModA13 target sites.

### The role of the *N. gonorrhoeae modA13* phasevarion in model systems

To determine whether the phasevarion mediated changes in gene expression correspond to altered phenotypes in model systems, we chose to focus on the *modA13* allele of *N. gonorrhoeae*. Several model systems were available from our previous studies on oxidative stress, biofilm formation and bacterial - host cell interactions [29]–[32]. Furthermore, strains FA1090 and O1G1370 provided an opportunity to test the reproducibility of key phenotypes in two independent *modA13* strains.

### Phasevarion switching alters resistance to an antimicrobial agent

**Figure 6:**
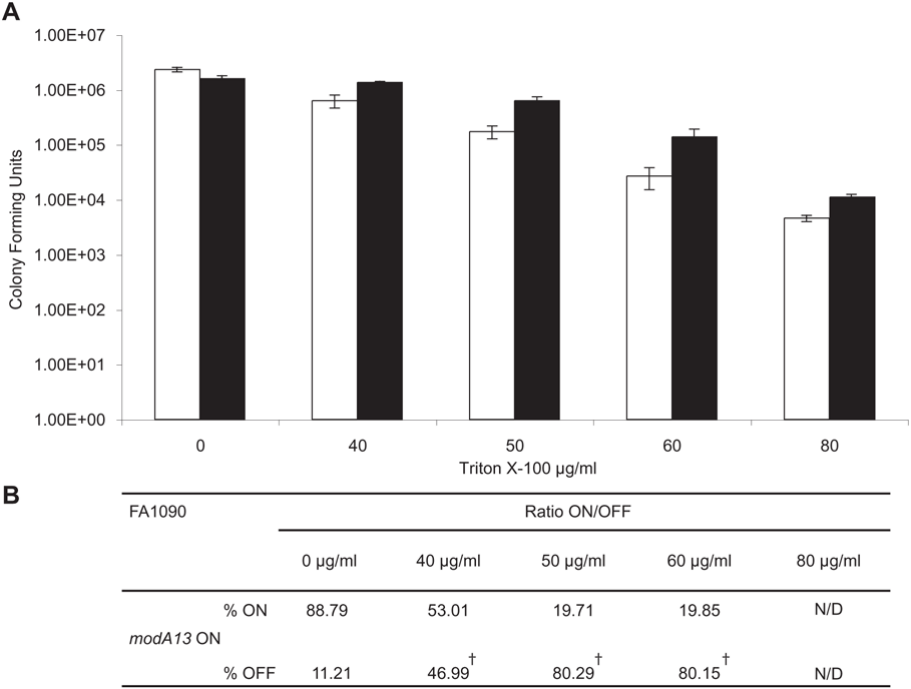
Comparison of wild-type FA1090 *modA13* ON and FA1090 *modA13::kan* mutant in an antimicrobial resistance assay. (A) Wild-type FA1090 *modA13* ON and FA1090 *modA13::kan* mutant cells were serially diluted and spotted onto GC plates containing increasing concentrations of Triton X-100 (x-axis) for determination of viable colony-forming units (y-axis). The white bars correspond to wild-type FA1090 *modA13* ON, and the black bars correspond to FA1090 *modA13::kan*. A Student's *t*-test showed a significant difference between the two samples (P≤0.021) at each of the following concentrations of Triton X-100; 40 µg/ml, 50 µg/ml, 60 µg/ml, and 80 µg/ml. (B) Shows the ratio of FA1090 *modA13* ON to FA1090 *modA13* OFF at each of the following concentrations of Triton X-100: 0 µg/ml, 40 µg/ml, 50 µg/ml, 60 µg/ml, and 80 µg/ml for FA1090 *modA13* ON. ^†^, a statistically significant difference was seen in the ON/OFF ratio between FA1090 *modA13* ON 0 µg/ml Triton X-100 and the following FA1090 *modA13* ON Triton X-100 concentrations: 40 µg/ml, 50 µg/ml, 60 µg/ml, indicating a selection to OFF organisms at these concentrations. N/D indicates not done. Calculations are shown in Table S8.

Previous studies using *N. gonorrhoeae* strain FA19 demonstrate that *mtrF* is required for induction of high-level antimicrobial resistance to Triton X-100 by gonococci [33]. Our data show that MtrF expression is up-regulated in the *modA13* mutant relative to the wild-type under iron-limiting conditions. To test whether differences in antimicrobial-resistance could be observed between wild-type FA1090 *modA13* ON and the FA1090 *modA13::kan* mutant, an antimicrobial-resistance assay was performed using increasing Triton X-100 concentrations (Figure 6A). The FA1090 *modA13::kan* mutant was found to be more resistant than wild-type FA1090 *modA13* ON, consistent with the higher level of expression of MtrF in this *modA13* OFF strain. As *modA13* ON is free to phase vary to OFF, and OFF cells appear to be fitter in this assay, the status of *modA13* expression was monitored by PCR with fluorescent primers across the repeat region to determine whether ON to OFF phase variants had been selected in the survivor colonies at various Triton X-100 concentrations. This analysis revealed that the FA1090 *modA13* ON culture plated on zero Triton X-100 remained ON, with only 11.21% OFF cells. However, cells plated on increasing Triton X-100 concentrations changed to 46.99% OFF, 80.29% OFF and 80.15% OFF over the course of the assay for 40, 50 and 60 µg/ml Triton X-100, respectively (Figure 6B).

### Phasevarion switching alters efficiency of biofilm formation in *modA13* strains

A number of studies have shown that *N. gonorrhoeae* can form a biofilm in a continuous-flow chamber and over primary human genital tract epithelial cells in culture [30],[31]. Biofilms provide a number of advantages in survival of the bacteria. It is suggested that biofilm formation by *N. gonorrhoeae* may contribute to its ability to persist in an asymptomatic state in the female genital tract [34]. In addition, bacteria within biofilms show increased resistance to antimicrobial agents [35],[36] and links between biofilm formation and oxidative stress defenses have been observed in *N. gonorrhoeae* [30].

**Figure 7:**
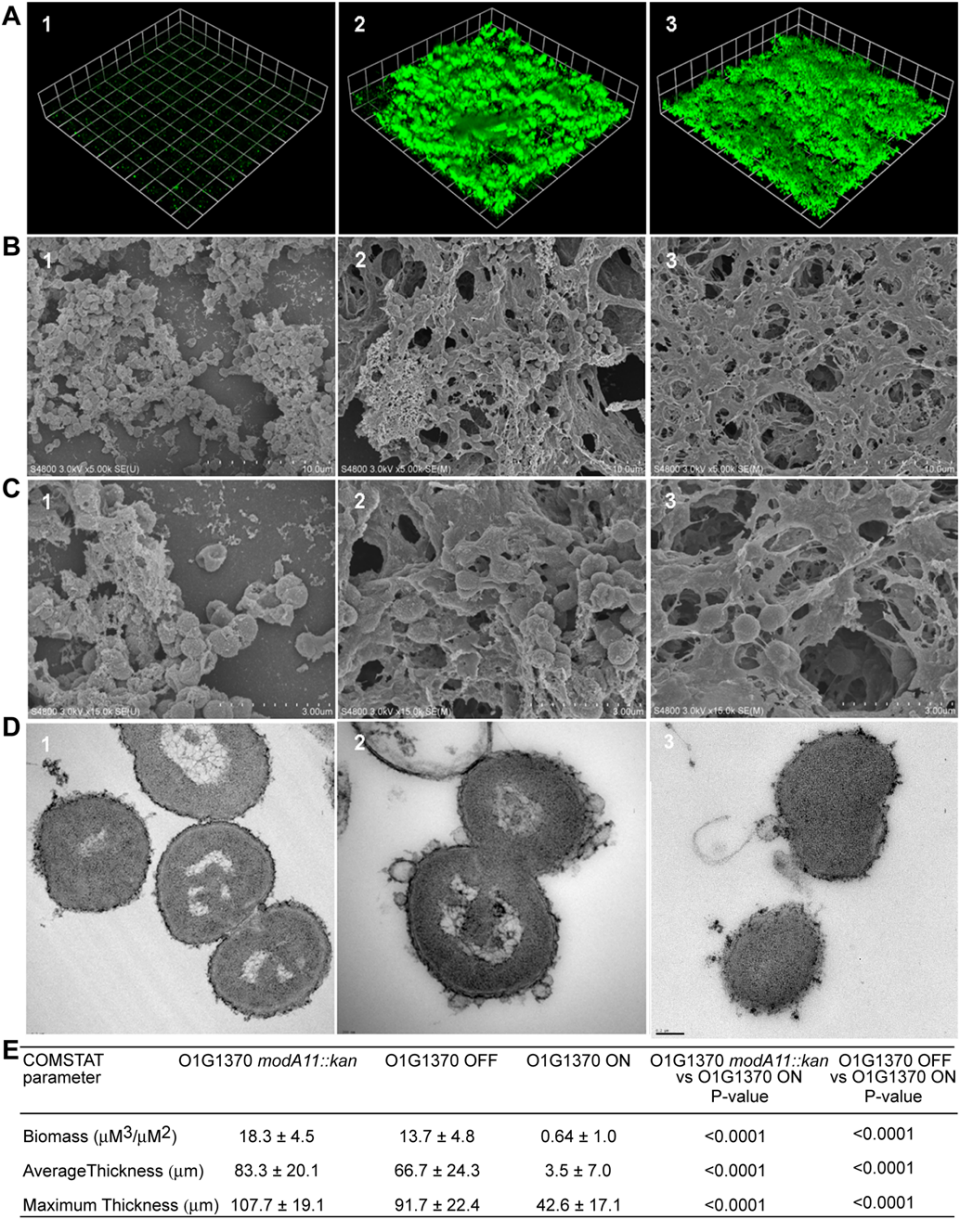
Biofilm formation by *N. gonorrhoeae* strain O1G1370 *modA13::kan*, *modA13* OFF, and O1G1370 *modA13* ON. (A) Confocal microscopy of the biofilm mass over 2 days of growth for (1) *N. gonorrhoeae* O1G1370 *modA13 ON*, (2) O1G1370 *modA13::kan*, and (3) O1G1370 *modA13* OFF. These images are three-dimensional reconstructions of stacked z-series taken at 200× magnification, which were rendered by Volocity. These experiments were performed in quadruplicate on three different occasions, and representative images are shown. (B) Scanning electron microscopy of the surface of the biofilm mass over 2 days of growth on glass taken at 5,000× magnification. It can be noted that there are fewer cells in the O1G1370 *modA13* ON biofilm than either the O1G1370 *modA13::kan* or O1G1370 *modA13* OFF biofilms. (C) Scanning electron microscopy of the surface of the biofilm mass over 2 days growth on glass taken at 15,000× magnification. (D) Transmission electron microscopy of 70 nm thin-sections of the biofilm mass over 2 days of growth on glass taken at 10,000× magnification. (E) COMSTAT analysis of biomass, average, and maximum thickness of confocal z-series images of the O1G1370 *modA13::kan*, O1G1370 *modA13* OFF, and O1G1370 *modA13* ON biofilms grown for 2 days over glass, which are depicted in (A). COMSTAT was performed for all replicates, and results are as shown. Statistical significance was determined using a Student's *t*-test. There was no statistically significant difference between the biomass, average, or maximum thickness of the O1G1370 *modA13::kan* and O1G1370 *modA13* OFF strains.

The ability of *N. gonorrhoeae* O1G1370 *modA13* ON, O1G1370 *modA13* OFF and O1G1370 *modA13::kan* (OFF) to form a biofilm was evaluated after two days of growth under continuous flow conditions. Three-dimensional images of these biofilms were created in Volocity (Materials and Methods). These images show that O1G1370 *modA13::kan* and *modA13* OFF form a thick and dense biofilm, while O1G1370 *modA13* ON forms an extremely weak biofilm with a few sparse patches of cells scattered across the surface of attachment (Figure 7A). The O1G1370 *modA13* ON strain also formed biofilms with lower maximum thicknesses than the O1G1370 *modA13::kan* and O1G1370 *modA13* OFF strains. (Figure 7D). Scanning electron microscopy of the surface of the biofilm taken at 5,000× magnification shows that there are gaps between clusters of biofilm in the O1G1370 *modA13* ON strain, unlike the O1G1370 *modA13* OFF and O1G1370 *modA13::kan* strain biofilms, where there are no areas where the glass surface of attachment is visible. There are also large areas where no biofilm is present in the O1G1370 *modA13* ON samples (Figure 7B). Scanning electron microscopy taken at 15,000× magnification shows that O1G1370 *modA13::kan* and O1G1370 *modA13* OFF form biofilms that are tightly enmeshed in an extracellular material that obscures the structure of individual cells, while cells in the *modA13* ON biofilm are clearly distinguishable (Figure 7C). Transmission electron microscopy shows that O1G1370 *modA13::kan* forms a biofilm where individual cells are shedding copious amounts of membrane, as seen in the numerous enclosed membrane blebs on the surface of the cells, while there is no evidence of blebbing in the O1G1370 *modA13* ON biofilm. Cells in the O1G1370 *modA13* OFF biofilm also appear to be blebbing, like those in O1G1370 *modA13::kan* biofilm, as numerous blebs can be seen forming on the surface of the O1G1370 *modA13* OFF strain. These electron micrographs suggest that the extracellular matrices of the O1G1370 *modA13::kan* and O1G1370 *modA13* OFF biofilms may be at least partially composed of fused membrane blebs (Figure 7C). COMSTAT [37] was used to quantitatively assess the biomass, and average and maximum thickness of confocal z-series photomicrographs taken for each flow chamber. COMSTAT analysis showed that the O1G1370 *modA13::kan* and O1G1370 *modA13* OFF strains form significantly thicker biofilms with significantly more biomass than the O1G1370 *modA13* ON strain. Specifically, O1G1370 *modA13* ON had 3.5% of the biomass and 4.2% of the thickness of the O1G1370 *modA13::kan* mutant on average and 4.7% of the biomass and 5.2% of the thickness of the O1G1370 *modA13* OFF (Figure 7E). Similar results were observed using *N. gonorrhoeae* strains FA1090 *modA13* ON and FA1090 *modA13::kan* (Figure S5).

### *ModA13* phase variation results in the altered fitness of *N. gonorrhoeae* strain O1G1370 to survive within primary human cervical epithelial cells

**Figure 8:**
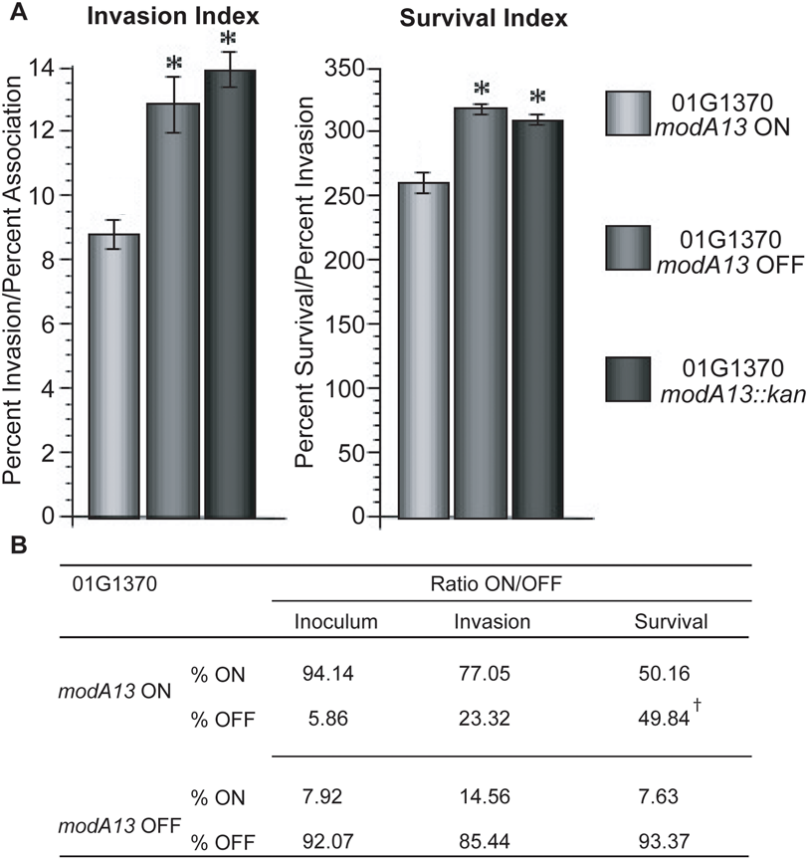
*N. gonorrhoeae* O1G1370 association with, and intracellular survival within, primary human cervical epithelial (pex) cells. Pex cells were challenged with *N. gonorrhoeae* strain O1G1370 as outlined in the text. Data shown represent the invasion index (left panel) or the survival index (right panel) following challenge of pex cells as outlined in the text. The invasion index represents the percentage of pex cell–associated gonococci that survive gentamicin treatment; whereas the survival index is the percentage of invasive gonococci that survive, intracellularly, within pex cells at 3 h post-invasion. There was no significant difference between the naturally occurring O1G1370 *modA13* OFF isolate and the O1G1370 *modA13::kan* “knockout” strain in either the invasion (P = 0.091) or survival (P = 0.23) indices observed. A statistically significant difference (*) was obtained in the invasion (P = 0.046) and survival (P = 0.021) indices when comparing O1G1370 *modA13* OFF to O1G1370 *modA13* ON, and in the invasion (P = 0.019) and survival (P = 0.004) indices when comparing O1G1370 *modA13::kan* to O1G1370 *modA13* ON. P-values were determined using a Student's *t*-test. (B) Shows the ratio of O1G1370 *modA13* ON to O1G1370 *modA13* OFF of the inoculum, and at the invasion and survival time points for O1G1370 *modA13* ON and O1G1370 *modA13* OFF. ^†^, a statistically significant difference was seen in the ON/OFF ratio between the O1G1370 *modA13* OFF inoculum and the O1G1370 *modA13* OFF survival sample (*P* = 0.0234), indicating a selection for OFF organisms over the course of the 3-h assay (for full data, see Table S8).

The use of primary human cervical epithelial (pex) cells as a model system of gonococcal cervicitis is well established and has been used in a number of studies, such as the examination of the role of oxidative stress regulators in host-pathogen interactions [29],[32]. To determine the biological significance of O1G1370 *modA13* expression using this pex cell culture model, we performed quantitative association, invasion, and survival assays using O1G1370 *modA13* ON, O1G1370 *modA13* OFF, and O1G1370 *modA13::kan* mutant gonococci (Figure 8). These data revealed that there was no significant (P≥0.2338) difference in the ability of the O1G1370 *modA13* OFF and O1G1370 *modA13::kan* strains to adhere to, invade, or survive within pex cells. This confirmed that the *modA13::kan* knockout allele behaves in the same way as a natural phase variant *modA13* OFF strain. In contrast, behavior of the O1G1370 *modA13* ON strain was significantly (P≤0.001) different from that obtained with the use of either the O1G1370 *modA13* OFF or O1G1370 *modA13::kan* strains in parallel assays. In this regard, a *modA13* ON phenotype resulted in the increased ability of gonococci to associate with pex cells, whereas a *modA13* OFF configuration augmented the ability of gonococci to invade (Figure 8A, invasion index) and survive within pex cells following invasion (Figure 8A, survival index). These data suggest a possible role for Mod-dependent phase variation in promoting the adaptive changes required for gonococci to switch from an extracellular to an intracellular existence. This idea is supported by our observation of selection for a switch from ON to OFF in the O1G1370 *modA13* ON strain. Fragment analysis confirmed that the O1G1370 *modA13* ON inoculum, which contains only 5.86% OFF, changes to ∼49.84% OFF by the time the 3 hour intracellular survival sample was taken (Figure 8B, Table S9). An independent *N. gonorrhoeae modA13* strain, 1291, displayed the same intracellular survival and *modA13* switching phenotype (Figure S6).

## Discussion

The pathogenic *Neisseria* are the archetypal organisms for the study of phase variation. Simple tandem repeats are typically associated with individual genes involved in biosynthesis of a surface component, such as an outer membrane protein, or a poly- or oligosaccharide. The consequence of hyper-mutation of these simple tandem repeats is phase variable expression of these genes, i.e., the presence or absence of a single component on the surface of the cell. Independent, random switching of many different phase variable genes encoding these surface structures leads to a combinatorial effect generating a huge number of alternate combinations of surface components. Phase variation, in conjunction with antigenic variation of the major antigen pili [38], leads to evasion of host immune responses. The distinction between the phasevarion and typical phase variation of genes encoding surface factors is that the ON/OFF switching of the phasevarion methyltransferase mediates expression changes in multiple genes in a coordinated manner [15].

Our phylogenetic studies on the *mod* genes of a collection of pathogenic *Neisseria* strains reveal that differences in the DNA recognition domain within the *mod* gene results in distinct *mod* alleles. R-M systems show extreme diversity in their DNA sequence recognition specificities. DNA sequence specificity in type III R-M systems is conferred by the Mod subunit [14]. Based on differences in the DNA recognition domain, three major *modA* alleles were found - *modA11*, *modA12*, *modA13*, and two distinct *modB* alleles were found - *modB1 and modB2*. This suggests the possibility that multiple phasevarions exist within the pathogenic *Neisseria*, each regulating a different set of genes. Furthermore, as each strain has both *modA* and *modB*, and these genes switch independently, there are four potential combinations of *mod* gene expression (ON/ON;ON/OFF;OFF/ON;OFF/OFF). We confirmed that two strains with the same DNA recognition domain (*modA13* allele) regulated the same set of genes, while, *N. meningitidis modA11* and *modA12* were found to regulate the expression of different sets of genes, consistent with differences in their DNA recognition domain. In this study we also identified the recognition sequence for ModA13 as 5′-AGAA^m^A-3′. In all, five randomly selected sites were tested for ModA13 inhibition of digestion in genomic DNA from *modA13* ON and OFF strains. All five sites tested displayed the expected inhibition of digestion phenotype (with either *Apo*I or *Hin*dIII), supporting the hypothesis that when expressed, ModA13 methylates all AGAAA sites in the genome, and thereby indicating this as the likely mechanism of gene control. Identification of the ModA13 target site is facilitating current studies on the molecular mechanism of regulation operating in the promoters of genes controlled by the *modA13* phasevarion (see Figure S7).

Gene regulation through the methylation of specific DNA sequences by methyltransferases has been reviewed [39],[40], but has focused on the role of Dam methylation. Dam methylation has been reported to be essential for bacterial virulence. In *Salmonella* species, *dam* mutants are highly attenuated for virulence and have been proposed as live vaccine candidates [41]–[43]. In addition, mutations in Dam attenuate the virulence of several other pathogens [44]–[46]. In these studies the mechanisms of attenuation (genes regulated) are unknown. In contrast, there are a few well-established examples of Dam mediated phase variation of genes encoding individual virulence factors[47]–[49], for example the pyelonephritis-associated pilus (*pap*) operon in uropathogenic *Escherichia coli* [47],[50],[51]. The fundamental characteristic of these DNA-methylation-dependent phase variable systems is that the target site's methylation state affects the DNA binding of a regulatory protein, which directly regulates transcription. The key point to note is that the Dam methyltransferase itself does not phase vary, nor are there any examples of Dam itself being regulated by an environmental signal. These systems are not analogous to phasevarions, but do provide examples of how DNA methylation may alter gene expression at a bacterial promoter. In the case of *N. meningitidis*, most strains have been found to be *dam* negative [52], as are all of the *N. meningitidis* and *N. gonorrhoeae* strains used in expression profile analysis and functional assay in this study (result not shown).

The question of whether the phase variable *mod* genes are associated with a functional type III restriction system remains to be fully resolved. In *H. influenzae* we have reported inactivating mutations in the *res* gene that is required for restriction function in strains containing phase variable *mod* genes [17]. We propose that in these cases the R-M system function has been lost and that the *modA* gene is dedicated to a gene regulation function. We have observed a similar inactivating mutation in the *res* gene associated with *modA11* of *N. meningitidis* (Table S2), and in *N. gonorrhoeae*, a 250 aa in-frame deletion has been observed in the *res* gene associated with *modA11* and *modA12* (see Table S3), supporting a dedicated gene regulation function for *mod* genes in pathogenic *Neisseria*.

Several of the genes regulated by the *modA11* phasevarion of *N. meningitidis* strain MC58 are outer membrane proteins, including the vaccine candidates LbpA and LbpB. These are typical of the class of gene that have evolved phase variation mechanisms under immune selection. It is clear that phasevarion mediated phase variation of candidate vaccine antigens has the potential to mediate escape from a vaccine primed immune response. In contrast, none of the typical genes encoding outer membrane structures were influenced by the *modA13* phasevarion in strain FA1090. Instead, the genes under phasevarion control were involved in functions such as oxidative stress, antibiotic resistance, and transport of nutrients. For example, the MetFE operon, which plays a role in the methylation of homocysteine, the final step of methionine biosynthesis. MetE catalyses the methylation of homocysteine using a methyl group that is donated by the *metF* gene product, 5-methyltetrahydrofolate [53]. In *E. coli*, a correlation is shown between oxidative stress, methionine availability, and MetE, where MetE is inactivated under conditions of oxidative stress [22]. In addition, MtrF, which is required for high-level, hydrophobic agent-resistance that is mediated by the MtrC-MtrD-MtrE efflux pump [26],[33], is controlled by the *modA13* phasevarion. The active efflux of antimicrobial agents from the cell by this systems is recognized as a major contributor to bacterial resistance to antibiotics [33],[54]. Altered expression of this group of genes is more consistent with a switch between cell types that are more suited to alternate physical environments, rather than switching to enable evasion of a particular primed immune response.

Phenotypic analyses of *modA13* ON, *modA13::kan* mutant or *modA13* OFF revealed distinct behavior in the model systems tested: *modA13::kan* and *modA13* OFF mutant cells were superior at formation of a biofilm. Bacteria within biofilms display increased resistance to antimicrobial agents [35],[36]. In addition, links between biofilm formation and oxidative stress defenses have been observed in *N. gonorrhoeae* [29]. Consistent with this observation, genes involved in oxidative stress and antimicrobial susceptibility were found to be up-regulated in expression in a *modA13::kan* mutant. Furthermore, a *modA13::kan* mutant was also found to be more resistant to antimicrobial agents in a Triton X-100 killing assay. Finally, the *modA13::kan* mutant and *modA13* OFF strains were more fit in an intracellular survival assay in the pex model system, and this assay selected for a switch from ON to OFF during the course of the 3 hour assay. These observations are consistent with random generation of two populations containing different cell types with distinct niche specialization. The observation of common biofilm and intracellular survival phenotype in independent *modA13* strains suggests that these can be attributed directly to *modA13* phase variation, rather than an independent phase variation event in an unrelated gene, and that these may be key aspects of gonococcal - host interactions. This study shows that all *modA* alleles regulate gene expression of many genes, and that we observe distinct behavior of, and switching between, *modA* ON and OFF states in model systems. It is clear that any future study of pathogenic *Neisseria* that investigates gene expression or behavior of strains in model systems should take *modA* phase variation into account.

Our initial microarray studies resulted in data with no statistically significant difference in the regulation of any gene. Changing the culture conditions to iron limitation resulted in the differential expression of 54 genes (Table S5). This change in expression was not due to differential growth rates (Figure S4) or a direct effect of iron on expression of the Mod methyltransferase (Figure S8). Clearly, a difference in gene expression can only be detected if the genes in question are being expressed. It is well established that many genes are expressed under iron-limiting conditions in pathogenic *Neisseria*, via the Fur regulon, either directly, or due to cascade effects resulting from activation of the Fur regulon [55],[56]. One limitation of the data presented in this study is that the gene expression profile analysis of the phasevarions was only done under one culture condition. Using different physiologically relevant conditions may enable other virulence-associated genes under the control of the phasevarion to be discovered. For example, it is established that the interaction of *N. meningitidis* with epithelial cells induces changes in the expression of 347 genes [57].

The results presented in this paper, in conjunction with our recent studies in *H. influenzae* [15],[17], provide confirmation of a role for phase variable *mod* genes associated with type III R-M systems in gene regulation. The widespread distribution of phase variable R-M systems in host-adapted pathogenic bacteria suggests that this novel mechanism of coordinated random switching of multiple genes may be a commonly used strategy for generation of distinct, “differentiated”, cell types with distinct niche specialization in host adapted bacterial pathogens.

## Materials and Methods

### Bacterial strains and growth conditions

*N. meningitidis* and *N. gonorrhoeae* strains were grown at 37°C with 5% CO_2_ in either GC broth or GC agar with IsoVitaleX (Becton Dickinson). *E. coli* strains DH5α and JM109 (Promega) were used to propagate plasmids and were grown at 37°C in Luria-Bertani (LB) broth supplemented with either ampicillin (100 µg/ml) or kanamycin (100 µg/ml).

### DNA manipulation and analysis

All enzymes were sourced from New England Biolabs. Sequencing was performed on PCR products using QiaQuick PCR purification kit (Qiagen) and Big-Dye (Perkin Elmer) sequencing kits. Data was analysed using MacVector v9.0 (Accelrys).

### *Mod* allele specific PCR

PCR products specific for the DNA recognition domain and repeat regions of *modA* and *modB* were generated using the primers listed in Table S7. *N. meningitidis* isolates [58] and *N. gonorrhoeae* DGI and MI clinical isolates [59] were used as templates. The reaction was performed in 50 µl using 1× *Taq* buffer, 1.5 mM MgCl_2_, and 1 unit of *Taq* DNA polymerase (Promega) with the following cycling conditions for the DNA recognition domain: 30 cycles of 94°C for 30 sec, 57°C for 30 sec, 72°C for 2 min and 1 cycle of 72°C for 7 min with 5 µM of the primer pair ModADRDF and ModADRDR for *modA* or ModBDRDF and ModBDRDR for *modB*. For *modA*, a 597 bp region containing the DNA recognition domain (393 bp downstream of ModADRDF and 101 bp upstream from ModADRDR) was compared to the genome strains to determine the *modA* allele group. For *modB* a 537 bp region containing the DNA recognition domain (461 bp downstream from ModBDRDF and 285 bp upstream from ModBDRDR) was compared to the genome strains to determine the *modB* allele group. The following cycling conditions were used for the repeat region: 30 cycles of 94°C for 30 sec, 57°C for 30 sec, 72°C for 30 sec and 1 cycle of 72°C for 7 min with 5 µM of the primer pair ModAF and ModAREPEATR or ModBREPEATF and ModBREPEATR. PCR products were cleaned using the QIAquick PCR Purification Kit (Qiagen).

### *Res* specific PCR

PCR products specific for the *res* gene were generated using the primers listed in Table S7. *N. meningitidis* isolates [58] and *N. gonorrhoeae* DGI and MI clinical isolates [59] were used as templates. The reaction was performed in 50 µl using 1× Taq buffer, 1.5 mM MgCl_2_, and 1 unit of Taq DNA polymerase (Promega) with the following cycling conditions for the DRD: 30 cycles of 94°C for 30 sec, 55°C for 30 sec, 72°C for 3 min and 1 cycle of 72°C for 7 min with 5 µM of the primer pair ResF and ResEDR2. PCR products were sequenced using ResF, ResR, ResEDF2 and ResEDR2.

### Nucleotide sequence manipulation and analysis

The *modA* nucleotide sequences were assembled using the Staden sequence analysis package [60] and all sequences aligned manually in the Seqlab alignment program (Genetics Computer Group, Madison, Wis.). Phylogenetic analysis was undertaken using the software package ClonalFrame version 1.1, which is a statistical model-based method initially described for inferring bacterial clonal relationships using multilocus sequence data [61]. Inference is performed in a Bayesian framework and a neutral coalescent model is assumed based on the hypothesis that the bacteria in the sample come from a constant-sized population in which each bacterium is equally likely to reproduce, irrespective of its previous history. The key assumption of ClonalFrame is that recombination events introduce a constant rate of substitutions to a contiguous region of sequence with the end result that a clonal frame can be inferred. In the present study, over 50,000 iterations were performed with every hundredth tree sampled after which, a 95% majority-rule consensus tree was derived. ClonalFrame is available at available at http://bacteria.stats.ox.ac.uk. The *modA* gene is composed of relatively conserved N and C-terminal regions with the DNA recognition domain in between. Consequently, sequence input into ClonalFrame was undertaken by firstly adding the N-terminal region starting at bp 359 in the *modA* gene belonging to the reference *N. meningitidis* isolate MC58, followed by the DNA recognition domain occurring from 416 to 795 bp and ending with the C-terminal region 796 to 1242 bp. Annotation was then undertaken by importing the tree into the Molecular Evolutionary Genetics Analysis software package (MEGA ver 4.0) [62].

Plasmid pCmGFP (Source M A Apicella) was extracted from *N. meningitidis* strain C311. Primers used to sequence this plasmid are listed in Table S7. Sequencing reactions were prepared using the plasmid as template and Big-Dye sequencing kit (Perkin-Elmer). Samples were analysed using a 3130×l Capillary Electrophoresis Genetic Analyser (Applied Biosystems International). Data were analysed and plasmid map constructed using MacVector (version 9.0). The plasmid sequence is deposited in GeneBank under accession number FJ172221).

### Identification of ModA13 modification site by inhibition of restriction assays

#### Plasmid restriction method

Plasmid pCmGFP was extracted from *N. gonorrhoeae* strain Fa1090 *modA13* ON and FA1090 *modA13::kan*, *N. gonorrhoeae* strain 96D551 *modA12* ON and *modA12::kan*, and *N. meningitidis* strain MC58 *modA11* ON and *modA11::kan* cells using the Qiagen Plasmid Midi Kit (Qiagen, Doncaster, Vic., Au). The *modA13* expression status of the ON cultures was verified by fragment analysis [17]. 2 micrograms of each plasmid was digested overnight with a range of restriction enzymes (*Acu*I, *Alu*I, *Apo*I, *Bsg*I, *Bsm*I, *Dde*I, *Dpn*II, *Dra*I, *Hinf*I, *Hpy*I88I, *Hpy*I88III, *Mbo*I, *Mbo*II, *Mse*I, *Msl*I, *Nla*III, *Taq*I, *Tsp*509I) according to manufacturer's instructions and the resulting fragments were separated on a 1.5% agarose gel with TBE at 120 V for 1 hour and visualised under UV illumination.

#### Chromosomal DNA restriction with Southern blot detection method

Chromosomal DNA was extracted from FA1090 *modA13* ON, *modA13* OFF and FA1090 *modA13::kan*. Mod expression status of the *modA13* ON and *modA13* OFF cultures were verified by fragment analysis. 1.6 micrograms of DNA was digested overnight with *Apo*I, *Alu*I and or mix of *Hind*III and *Rsa*I according to manufacturer's instructions. Fragments were separated on 0.8% agarose gels in TBE at 100 V for 1–2 hours and visualised under UV illumination. Southern transfer and hybridisation analysis was carried out as described by Sambrook et al [63] using DIG-labelled (Roche) PCR products as probes. Probes were amplified using the primers listed in Table S7 to investigate restriction sensitivity at the respective ModA13/restriction site overlaps.

### Construction of a translation fusion between the *modA* gene and *lacZ* gene and insertion into *N. meningitidis* strain MC58

A *modA::lacZ* fusion was constructed in *N. meningitidis* MC58. The gene fusion was initially constructed in *E. coli* with subsequent transformation into the *N. meningitidis* chromosome. In the fusion construct, the codons for LacZ are in the same translational frame as ModA resulting in an in-frame Mod-LacZ fusion protein. A 4 kb fragment of a promoterless *lacZ::kan* fragment was amplified by PCR using the primer pair LacZ*Sty*I1 and Kan*Sty*I. The plasmid pBluescript*lacZ::kan* was used as template. Following digestion with *Sty*I, the 4.0 kb *lacZ::kan* fragment was then ligated into the *Xba*I site of pGEM*modA*. The ligation mixture was transformed into *E. coli* JM109 and transformants were selected on LB agar plates supplemented with kanamycin (50 µg/ml; Sigma). The orientation and sequence of the insert were checked and found to be correct. The resulting construct was named pGEM*modA::lacZ::kan*. This plasmid was linearized with *Sac*II and used to transform competent *N. meningitidis*. The MC58*modA*::*lacZ::kan* transformants were streaked on BHI plates containing Levinthal supplement and X-gal (40 µg/ml).

### Construction of knockout mutants of the *modA11*, *modA12*, and *modA13* gene and insertion into *N. meningitidis* strain MC58, *N. meningitidis* strain B6116/77, *N. gonorrhoeae* strain FA1090, clinically isolated *N. gonorrhoeae* strains O1G1370 and 96D551

The *modA* open reading frame (ORF) was amplified using PCR with primers ModAF and ModAR (see Figure S1). *N. meningitidis* strain MC58 was used as template. The PCR product was cloned into vector pGEM-Teasy (Promega) and named pGEM*modA*. The pGEM*modA* construct was digested with *Xba*I and blunt ended using Klenow Polymerase (New England Biolabs). The Tn*903 kan* resistance gene from the pUC4K vector (Pharmacia) was excised using *Hinc*II and inserted into the blunt *Xba*I site. Previous work has demonstrated that the pUC4Kan kanamycin cassette has no promoter or terminator that is active in *Neisseria* and will neither affect transcription nor have a polar effect on expression of adjacent genes [29]. The resulting plasmid, pGEM*modA*::*kan* was linearized by digestion with *Sph*I and used to transform competent *N. meningitidis* strains MC58 and B6116/77 or *N. gonorrhoeae* strains FA1090, O1G1370 or 96D551. MC58 *modA11::kan*, B6116/77 *modA12::kan*, FA1090 *modA13::kan* O1G1370 *modA13::kan* and 96D551 *modA12::kan* transformants were selected on BHI plates containing Levinthal supplement and 100 µg/ml kanamycin. Transformants were confirmed by PCR and sequence analysis using primers ModAF2 and kanamycin specific primers. RNA midi-preps of both the wild-type (MC58 *modA11* ON, B6116/77 *modA12* ON, FA1090 *modA13* ON and O1G1370 *modA13* ON) and mutant (MC58 *modA11::kan*, B6116/77 *modA12::kan*, FA1090 *modA13::kan* and O1G1370 *modA13*::*kan*) were made using the RNeasy Midiprep kit (Qiagen). Wild-type colonies, from which RNA was isolated for microarray analysis, were sequenced using primers ModAF and ModAREPEATR to check that the *mod* repeat region was in-frame.

### RNA extraction

Triplicate cultures of *N. meningitidis* strain MC58 *modA11* ON and the MC58 *modA11::kan* mutant, *N. meningitidis* strain B6116/77 *modA12* ON and the B6116/77 *modA12::kan* mutant or *N. gonorrhoeae* strain FA1090 *modA13* ON and the FA1090 *modA13::kan* mutant, O1G1370 *modA13* ON and the O1G1370 *modA13::kan* and 96D551 *modA12* ON and 96D551 *modA12::kan* mutant were grown to exponential phase (optical density at 600 nm = 0.5 to 0.6) with 30 µM desferal (Sigma) in GC broth prior to RNA extraction. Growth rates of strain pairs used to make RNA for microarray comparison were determined (Figure S4) and were found to be equivalent ensuring that the samples taken were in the same growth phase. Only 96D551 *modA12* ON and 96D551 *modA12::kan* (OFF) strains showed a significant difference in growth rate (see Figure S4). Culture media for RNA preps was free of antibiotics as once the *modA::kan* mutation is transferred to the chromosome by double crossover we observed that it is stable without selection. Approximately 100 µg of total RNA was prepared from each sample using the RNeasy Maxi Kit according to the manufacturer's instructions (Qiagen). The triplicate samples were pooled and the integrity and concentration of RNA was determined via micro-fluidic analysis on a bio-analyser (Agilent Technologies).

### Microarray analysis

All microarray analysis was performed on *N. gonorrhoeae/meningitidis* genome arrays (TIGR; http://pfgrc.tigr.org/). Each microarray consists of 6,389 70mer oligonucleotides representing open reading frames (ORFs) from *N. gonorrhoeae* strains FA1090 and ATCC 700825 (reference strain), as well as *N. meningitidis* strains Z2491 (serogroup A) and MC58 (serogroup B). Methods and analysis were as previously described [29]. All primary data was imported into an in-house installation of the comprehensive microarray relational database, BASE (accessible at: http://kidney.scgap.org/base login: Nmmod, password: Nmmod, login: NmmodA12, password: NmmodA12, login: NgmodA12, password: NgmodA12 or login: Ngmod, password: Ngmod).

### Quantitative real-time PCR

Oligonucleotides (Table S7) were designed using Primer Express 1.0 software (ABI Prism; PE Biosystems) and are named according to the ORF being amplified. All real-time PCR reactions were performed in a 25 µl mixture containing 1/5 volume of cDNA preparation (5 µl), 10XSYBR Green buffer (PE Applied Biosystems) and 2 µM of each primer. We used 16S RNA as the standard control in each quantitative PCR. Amplification and detection of specific products were performed with the ABI Prism 7700 sequence-detection system (PE Applied Biosystems) with the following cycle profile: 95°C for 10 min, followed by 45 cycles of 95°C for 15 sec and 60°C for 1 min. Data was analysed with ABI prism 7700 (version 1.7) analysis software. Relative gene expression between the MC58 *modA11::kan* mutant and wild-type MC58 *modA11* ON, *N. meningitidis* strain B6116/77 *modA12* ON and the B6116/77*modA12::kan* mutant or the FA1090 *modA13::kan* mutant and wild-type FA1090 *modA13* ON was determined using the 2^ΔΔCT^ relative quantification method.

### Semi-quantitative RT-PCR

Total RNA was isolated using the RNeasy kit (Qiagen). The equivalent of 1 µg of the total RNA preparation was treated with RQ1 RNase-free DNase (Promega). RT-PCR was performed using the TaqMan RT-PCR kit (PE Applied Biosystems) as recommended by the manufacturer. PCR was carried out in 50 µl using 1× Taq buffer, 1.5 mM MgCl_2_, and 1 unit of Taq DNA polymerase (Promega) and cDNA amplified using gene specific primers designed for Real-time PCR (Table S7) with the following cycling conditions: 30 cycles of 94°C for 30 s, 50°C for 30 s, 72°C for 30 s and 1 cycle of 72°C for 7 min. 16S rRNA internal standards for comparison were used with amplification resulting in a 200 bp RT-PCR product. PCR products (20 µl) were run on a 3% agarose gel.

### Growth studies comparing wild-type *mod* ON to the *modA::kan* mutants

Growth experiments were carried out in GC medium supplemented with IsoVitaleX, at 37°C with 5% CO2, under iron-limiting conditions (30 µM desferal). Triplicate cultures of the strain pairs being compared were adjusted to an identical initial OD_600_. One milliliter of culture was removed at fixed times to measure the OD_600_.

### Analysis of LbpA expression

Wild-type MC58 *modA11* ON, wild-type MC58 *modA11* OFF and MC58 *modA11::kan* mutant bacterial cells were grown under iron-limiting conditions to an optical density at 600 nm = 0.55–0.6. Cells were spun down at 5000 rpm for 5 min and then washed once in PBS, pH 7.2. Cells were then re-suspended in PBS to an optical density at 600 nm = 2.5 and separation was carried out on a 4–12% Nu-PAGE Novex Bis-Tris gel (Invitrogen) according to the manufacturer's instructions. The Nu-PAGE semi-dry system was used to transfer protein from gel to nitrocellulose membrane (0.22 µM pore, Bio-Rad), as recommended by Invitrogen. Immunoblotting of membranes was carried out in a 1∶2000 dilution of LbpA specific monoclonal 269-H1 [19] in 5% skimmed milk powder in TBS. Bands were visualized following incubation in 1∶5000 dilution of alkaline phosphatase-conjugated anti-mouse IgG secondary antibody (Sigma).

### Construction of a translation fusion between the *lbpB* gene and *lacZ* gene and insertion into *N. meningitidis* strain MC58

An *lbpB::lacZ* fusion was constructed in *N. meningitidis* strain MC58. The gene fusion was initially constructed in *E. coli* with subsequent transformation into the *N. meningitidis* chromosome. In the fusion construct, the codons for LacZ are in the same translational frame as *lbpB* resulting in an in-frame LbpB-LacZ fusion protein. A 1.7 kb DNA fragment was amplified by PCR using the primer pair LbpBF and LbpBR. MC58 was used as the template. The reaction was performed in 50 µl using 1× *Taq* buffer, 1.5 mM MgCl_2_, and 1 unit of *Taq* DNA polymerase (Promega) with the following cycling conditions: 30 cycles of 94°C for 30 sec, 57°C for 30 sec, 72°C for 1 min and 1 cycle of 72°C for 7 min. The fragment was then cloned into vector pGEM-Teasy (Promega). A 4 kb fragment of a promoterless *lacZ::kan* fragment was amplified by PCR using the primer pair LacZ*Sty*I+1 and Kan*Sty*I. The plasmid pBluescript*lacZ::kan* (M. Dieckelman, personal communication) was used as template. Following digestion with *Sty*I, the 4.0 kb *lacZ::kan* fragment was blunted using Klenow Polymerase and then inserted into the *EcoR*V site of the *lbpB* construct. The ligation mixture was transformed into *E. coli* JM109 and transformants were selected on LB agar plates supplemented with kanamycin (50 µg/ml). The orientation and sequence of the insert were checked and found to be correct. The resulting construct was named pGEM*lbpB::lacZ::kan*. This plasmid was linearized with *Nco*I and used to transform competent *N. meningitidis* strain MC58 with a naturally derived number of *mod* ON and OFF repeats. The MC58*lbpB*::*lacZ::kan mod* ON and MC58*lbpB*::*lacZ::kan mod* OFF transformants were streaked on BHI plates containing Levinthal supplement and X-gal (5-bromo-4-chloro-3-indolyl-D-galactopyranoside; 40 µg/ml).

### β-galactosidase assay

MC58*lbpB*::*lacZ::kan modA11* OFF and MC58*lbpB*::*lacZ::kan modA11* ON strains were grown on GC plates with 15 µM desferal at 37°C over night. The next day triplicate cultures of iron-starved strains were grown to exponential phase (optical density at 600 nm = 0.55 to 0.6) with 30 µM desferal in GC broth. Cells were spun down at 15,000×g for 10 min, resuspended in PBS and lysed by repeated freeze-thaw cycles. The cells debris was spun down at 15,000×g for 5 min. The amount of protein was calculated by using the BCA protein assay reagent kit (Pierce). The amount of β-galactosidase in the cell extracts was measured in Miller units, in triplicate, as described [64]. Miller units were calculated as follows: Units (1000×A_420_)/(*t*×*v*×*C*), where *t* is the time of the assay (in mins), *v* is the volume of cell extract used in the assay, and *C* is the total protein concentration (in µg/ml).

### Antimicrobial resistance assay

The antimicrobial resistance assay was adapted from a method described by Dougherty *et al.* [65]. In brief, *N. gonorrhoeae* FA1090 wild-type *modA13* ON and FA1090 *modA13::kan* mutant colonies were re-suspended in PBS to a density of 10^6^ colony forming units (CFUs), and 5 µl of serial ten-fold dilutions were spotted in triplicate onto GC agar plates containing 15 µM desferal, supplemented with IsoVitaleX and increasing concentrations of Triton X-100 (40, 50, 60, and 80 µg/ml). The plates were then incubated at 37°C under 5% CO_2_ for 24 h. Colony counts were used to compare wild-type FA1090 *modA13* ON to the FA1090 *modA13::kan* mutant by plating each dilution in triplicate. The experiment was repeated on three separate occasions. The ratio of FA1090 *modA13* ON to FA1090 *modA13* OFF at the following concentrations of Triton X-100 (40, 50 and 60 ug/ml) was calculated as follows. Colonies were taken from the triplicate samples of the original inoculum and each of the increasing concentrations of Triton X-100 from FA1090 *modA13* ON and used as PCR template. The percentage of *modA13* ON and *modA13* OFF from the starting inoculum and the three different Triton X-100 concentrations was verified via fragment analysis [17] using primers ModAF6Fam and ModAREPEATR (Table S7). A Student's *t*-test was used to determine the statistical significance between the percentage of *modA13* ON and *modA13* OFF from the original inoculum of *modA13* ON and the percentage of *modA13* ON and *modA13* OFF from the three different Triton X-100 of *modA13* ON.

### Biofilm formation by *N. gonorrhoeae*

For examination of biofilm formation via confocal microscopy, the *N. gonorrhoeae* FA1090 *modA13::kan* and wild-type FA1090 *modA13* ON strains and *N. gonorrhoeae* strains O1G1370 *modA13::kan*, *modA13* OFF and *modA13* ON were transformed with a plasmid encoding a green fluorescent protein, pCmGFP. Formation and analysis of biofilms was as described previously, except the cells were grown under the same iron-limiting conditions as for the microarray analysis [29]. Colonies used to inoculate cultures for biofilm assays were assessed for morphology to ensure equivalent level of piliation. Biofilms images are three-dimensional reconstructions of stacked z-series taken at 200× magnification, which were rendered by Volocity.

### Electron microscopy

Biofilms of *N. gonorrhoeae* strain FA1090 *modA13::kan* and *modA13* ON and *N. gonorrhoeae* strains O1G1370 *modA13::kan*, *modA13* OFF and *modA13* ON were grown at in glass flow chambers at 37°C and a flow rate of 180 µl/min in 1∶10 GC broth diluted in PBS with 10 ml/L IsoVitaleX, 3 µM desferal, and 100 µM sodium nitrite. The *modA13* status of the starting inoculum was verified via fragment analysis [17] using primers ModAF6Fam and ModAREPEATR. After 48 hours of growth, biofilms were prepared for scanning electron microscopy (SEM) and transmission electron microscopy (TEM) as follows. Glass coverslips, which served as the surface of attachment for biofilm, were removed from the chambers and fixed in 1% osmium perfluorocarbon for 1 h. The coverslips were then gently rinsed for 15 min with pure perfluorocarbon three times. To avoid destruction of the biofilm, rinse solution was gently added to coverslips in a 100 mm Petri dish, allowed to incubate at room temperature for 15 min, then the rinse was aspirated and another rinse was applied. The samples were then dehydrated with 100% ethanol by performing another three 15 min rinses. At this point, the coverslips were cut in half and one half was processed for SEM, while the other half was processed for TEM. SEM samples were transitioned into HMDS for two 15 min washes and then allowed to air dry. SEM samples were then sputter-coated and viewed with the Hitachi S-4800 SEM. TEM samples were infiltrated with a 50% Eponate-12 resin (epon) in ethanol for 1 h. The coverslips were then inverted and imbedded in 100% epon at 42°C overnight. Thin-sections (70 nm) were prepared on an ultramicrotome, mounted on a grid, and then stained with uranyl acetate and lead citrate. TEM samples were viewed with the JEOL 1230 TEM.

### Primary, human, cervical epithelial cell culture and infection studies

Surgical cervical biopsies were used to seed primary cervical epithelial (pex) cell cultures and were procured and maintained as described previously [32]. Quantitative association, invasion, and survival assays were performed as previously described using a multiplicity of infection of 100 [32] with modification as follows. Our previous studies demonstrate that pex cells produce a full alternative pathway of complement, and that iC3b serves as a critical opsonin for CR3-mediated gonococcus adherence to and invasion of these cells. Thereby, antibiotic-free medium was harvested from uninfected pex cell monolayers and treated overnight with 30 µM desferal (Sigma). Our previous (unpublished) studies have revealed that *N. gonorrhoeae* strain FA1090 uniquely becomes cytotoxic to human, primary cervical and male urethral epithelial cells within 2 to 3 hours post-challenge, which prohibits their confident use in gentamicin survival assays for time periods totaling greater than 90 min. Therefore, *N. gonorrhoeae* strains O1G1370 *modA13* ON, O1G1370 *modA13* OFF, and the O1G1370 *modA13::kan* mutant, and 1291 *modA13* ON, 1291 *modA13* OFF, and 1291*modA13::kan* were selected to elucidate the role of *mod*-dependent phase variation during pex cell challenge. Complement-containing, iron-depleted, primed medium was inoculated with 5×10^6^ gonococci per ml. Colonies used to inoculate cultures for these assays were assessed for morphology to ensure equivalent level of piliation. Bacterial cultures were incubated (37°C, with shaking) for 2 h, after which the optical density of the gonococcal cultures was adjusted to 10^7^ gonococci per ml and directly used to challenge (new) pex cell monolayers. Pex cell infections were then allowed to progress at 37°C, 5% CO_2_. Association (90 min infection), invasion (90 min infection plus a 30 min incubation in 100 µg/ml gentamicin), and survival (90 min infection, 30 min gentamicin treatment, plus a 3 h incubation in antibiotic-free medium) assays were performed using a modified gentamicin-resistance assay as described previously [32].Serial dilutions of the cervical cell lysates were plated to determine CFUs. The percent association, invasion, and survival were determined as functions of the original inoculum. From these data the invasion and survival indices were determine as follows: Invasion index, percent invasion/percent association; Survival index, percent survival/percent invasion. P-values were determined for the actual data points using a Kruskal-Wallis non-parametric analysis of variance. A Student's *t*-test was used to determine the statistical significance of the invasion and survival indices. The ratio of *modA13* ON and *modA13* OFF within the O1G1370 *modA13* ON and O1G1370 *modA13* OFF original inoculum, association, invasion, and survival time points were determined as follows. Samples were taken from the original inoculum, association, invasion, and survival time points from three independent assays and chromosomal DNA extracted. The percentage of *modA13* ON and *modA13* OFF from the starting inoculum was verified via fragment analysis [17] using primers ModAF6Fam and ModAREPEATR (Table S7). A Student's *t*-test was used to determine the statistical significance between the percentage of *modA13* ON and *modA13* OFF from the original inoculum of O1G1370 *modA13* ON and the percentage of *modA13* ON and *modA13* OFF from the invasion time point and survival time point of O1G1370 *modA13* ON. Similarly, a Student's *t*-test was used to determine statistical significance between the percentage of *modA13* ON and *modA13* OFF from the original inoculum of O1G1370 *modA13* OFF and the percentage of *modA13* ON and *modA13* OFF from the invasion time point and survival time point of O1G1370 *modA13* OFF.

### Production of anti-Mod antisera

The *mod* gene was amplified from *H. influenzae* strain Rd chromosomal DNA using primers listed in Table S7. The *Nco*I restriction site at the 5′ end and the *Bam*HI site at the 3′ end of the *mod* gene were introduced. The resulting PCR fragment was subsequently digested with *Nco*I and *Bam*HI and cloned into the digested pET16b expression vector (Novagen & EMD, San Diego, CA, USA) carrying the same enzyme cutting sites, leading to the construct, pET16b::*mod*. The sequence of the insert was confirmed and then used for generating the recombinant Mod protein with the (His)_10_-tag (MGHHHHHHHHHH) attached at the N-terminal end. For generating the recombinant Mod protein, the construct, pET16b::*mod*, was transformed into *E. coli* strain BL21(DE3) and the cells were grown in LB broth at 20°C. Induction of the expression was initiated by adding IPTG to the final concentration of 0.1 mM and then incubated at 10°C for 3 days. After harvesting the bacteria by centrifuging at 6,000 rpm for 30 min at 4°C, the bacterial pellet was lysed with the lysis buffer (25 mM Tris-HCl, 300 mM KCl, 5 mM imidazole, pH 7.5) plus protease inhibitor, Complete cocktail EDTA-free (Roche, Switzerland). Soluble proteins were obtained from the supernatant by centrifuging at 20,000 rpm for 20 min at 4°C to remove the cell debris and precipitates. The Mod protein was purified using the Ni^2+^-nitilotriacetic acid (Ni-NTA) column (Amersham Biosciences, Piscataway, NJ, USA) with an elution gradient from 25–500 mM imidazole in the buffer solution (25 mM Tris-HCl, 300 mM KCl, pH 7.5). The purity of the eluted protein was examined by SDS–PAGE analysis and the concentration determined by Bio-Rad Protein Assay (Bio-Rad, Hercules, CA, USA). The pure fractions were collected and transferred to 25 mM Tris, pH 7.5 by the HiPrep 26/10 Desalting column (Amersham Biosciences, USA) and store at −80°C.

Rabbits (New Zealand White strain, weighing 3–3.5 kg, were immunized by intrasplenic injection with the soluble recombinant Mod protein at 300 µg per immunization. The antigen was administered together with an equal amount of Gold TiterMax adjuvant (CytRx, Norcross, GA, USA). The rabbit antisera were collected from weeks 4∼9 and the titers of rabbit sera from weeks 4∼6 were analyzed using Western blot assays. Antiserum of week 6 had a high titer of 5,000,000 against 1 µg of the Mod protein. The antisera recognized a single band in wild type *modA* ON *H. influenzae* strain RD and *N. meningitidis* strain MC58, but not in their corresponding *modA::kan* mutants (not shown). For the subsequent Western blot experiments, 1/1,000 dilution of the antiserum of week 6 was used.

## Supporting Information

Figure S1 Schematic of the construction of pGEM*modA::lacZ::kan* and subsequent transformation into *N. meningitidis*. (A) Insertion of the *lacZ::kan* cassette into the *modA* ORF. (B) Transformation into *N. meningitidis* strain MC58. (C) Double crossover event results in insertion of the plasmid into the MC58 chromosome resulting in strain MC58*modA::lacZ::kan*.
(0.13 MB PDF)

Figure S2 Schematic of the construction of pGEM*modA::kan* and subsequent transformation into *N. meningitidis* or *N. gonorrhoeae*. (A) Insertion of the kanamycin (kan) cassette into the *mod* ORF. (B) Transformation into *N. meningitidis* strain MC58, *N. meningitidis* strain B6116/77, *N. gonorrhoeae* strain FA1090, or *N. gonorrhoeae* strain 96D551. (C) A double crossover event results in insertion of the plasmid; into the MC58 chromosome resulting in MC58 *modA11::kan* mutants, into the B6116/77 chromosome resulting in B6116/77 *modA12::kan* mutants, into the FA1090 chromosome resulting in FA1090 *modA13::kan* mutants, and into the 96D551 chromosome resulting in 96D551 *modA12::kan* mutants.
(0.18 MB PDF)

Figure S3 Schematic representation of the construction of pGEM*lbpB::lacZ::kan* and subsequent transformation into *N. meningitidis*. (A) Insertion of the *lacZ::kan* cassette into the *lbpB* ORF. (B) Transformation into *N. meningitidis* strain MC58 with a naturally derived number of *modA11* OFF repeats and *N. meningitidis* strain MC58 with a naturally derived number of *modA11* ON repeats. (C) Double crossover event results in insertion of the plasmid into the MC58 *modA11* OFF and MC58 *modA11* ON chromosome resulting in strains MC58*lbpB::lacZ::kan modA11* OFF and MC58*lbpB::lacZ::kan modA11* ON.
(0.14 MB PDF)

Figure S4 Growth rate comparisons of MC58 *modA11* ON and MC58*modA11::kan*, FA1090 *modA13* ON and FA10908 *modA13::kan*, B6116/77 *modA12* ON and B6116/77 *modA12::kan*, and 96D551 *modA12* ON and 96D551 *modA12::kan*. The optical density of wild-type and mutant cells, grown under the same iron-limiting conditions as used for expression and functional studies (see Materials and Methods), was measured and the differences in growth rate compared. The generation time was calculated from the slope of the line obtained in the logarithmic plot of exponential growth for each set of wild-type and mutant triplicates. The growth rate (minutes) was determined by 1/generation time. No significant difference in growth rate was observed between (A) MC58 *modA11* ON and the MC58 *modA11::kan* mutant (P = 0.393), (B) FA1090 *modA13* ON and FA10908 *modA13::kan* (P = 0.068), (C) *B6116/77modA12* ON and *B6116/77modA12::kan* (P = 0.363). However, a significant difference in growth rate was observed between 96D551 *modA12* ON and 96D551 *modA12::kan* (P = 0.047). P-values were calculated using a Student's *t*-test.
(0.14 MB PDF)

Figure S5 Biofilm formation by *N. gonorrhoeae* strain FA1090 *modA13::kan* and wild-type FA1090 *modA13* ON. The ability of wild-type FA1090 *modA13* ON and *N. gonorrhoeae* FA1090 *modA13::kan* to form a biofilm was evaluated after two days of growth under continuous flow conditions. These experiments were performed in duplicate on three different occasions and representative images are shown. (A) Confocal microscopy of the biofilm mass over 2 days of growth for the *N. gonorrhoeae* wild-type FA1090 *modA1*3 ON (1) and FA1090 *modA13::kan* mutant (2). These images are three-dimensional reconstructions of stacked z-series taken at 200× magnification, which were rendered by Volocity (see Materials and Methods). These images show that, overall, wild-type FA1090 *modA13* ON formed a thinner and more diffuse biofilm with large gaps between biofilm clusters, while the FA1090 *modA13::kan* mutant formed a thicker and more densely packed biofilm with very few gaps occurring between biofilm clusters. (B) Scanning electron microscopy of the surface of the biofilm mass over 2 days of growth on glass taken at 5,000× magnification. The images show that FA1090 *modA13::kan* forms a biofilm that is tightly enmeshed in extracellular material that obscures the structure of individual cells. Cells in the FA1090 *modA13* ON biofilm are clearly distinguishable and exhibit a normal blebbing phenotype. (C) Transmission electron microscopy of 70 nm thin-sections of the biofilm mass over 2 days of growth on glass taken at 10,000× magnification. The electron micrographs depicted are representative of images taken for *modA13* ON and *modA13::kan* in two independent experiments. The images show that FA1090 *modA13::kan* forms a biofilm with a hyper-blebbing phenotype, as seen in the numerous enclosed membranes on the surface of the cells, while the FA1090 *modA13* ON biofilm exhibit a wild-type blebbing phenotype with fewer blebs on the surface of the cells. The electron micrographs suggest that the extracellular matrix of the FA1090 *modA13::kan* biofilm may be at least partially composed of fused membrane blebs. (D) COMSTAT analysis of biomass and the average thickness of confocal z-series images of the *modA13* ON and FA1090 *modA13::kan* mutant biofilms grown for 2 days over glass, which are depicted in (A). COMSTAT analysis showed that FA1090 *modA13::kan* exhibited enhanced biofilm formation as compared to wild-type FA1090 *modA13* ON gonococci. Specifically, wild-type FA1090 *modA13* ON had 21.8% of the biomass and 49.7% of the thickness of the FA1090 *modA13::kan* mutant on average. The FA1090 *modA13::kan* mutant also formed biofilms with a slightly lower maximum thicknesses than wild-type FA1090 *modA13* ON, but this result was not statistically significant as determined by a Student's *t*-test. COMSTAT was performed for all replicates, and results are as shown. Statistical significance was determined using a Student's *t*-test.
(3.08 MB PDF)

Figure S6 *N. gonorrhoeae* 1291 association with, and intracellular survival within, primary human cervical epithelial (pex) cells. Pex cells were challenged with *N. gonorrhoeae* strain 1291 as outlined in the main text. Data shown represent the invasion index (left panel) or the survival index (right panel) following challenge of pex cells as outlined in the main text. The invasion index represents the percentage of pex cell-associated gonococci that survive gentamicin treatment; whereas the survival index is the percentage of invasive gonococci that survive, intracellularly, within pex cells at 3 h post-invasion. There was no significant difference between the naturally occurring 1291 *modA13* OFF isolate and the 1291 *modA13::kan* “knockout” strain in either the invasion (P = 0.254) or survival (P = 0.806) indices observed. A statistically significant difference (*) was obtained in the invasion (P = 0.008) and survival (P = 0.001) indices when comparing 1291 *modA13* OFF to 1291 *modA13* ON, and in the invasion (P = 0.037) and survival (P = 0.001) indices when comparing 1291 *modA13::kan* to 1291 *modA13* ON. P values were determined using a Student's *t*-test. (B) Shows the ratio of 1291 *modA13* ON to 1291 *modA13* OFF of the inoculum, and at the invasion and survival time points for 1291 *modA13* ON and 1291 *modA13* OFF. †A statistically significant difference was seen in the ON/OFF ratio between the 1291 *modA13* OFF inoculum sample and the 1291 *modA13* OFF invasion sample (P = 0.0082) and the 1291 *modA13* OFF inoculum sample and the 1291 *modA13* OFF survival sample (P = 0.0333), indicating a selection for OFF organisms over the course of the 3-h assay.
(0.34 MB PDF)

Figure S7 Genes regulated by ModA13 in FA1090 containing ModA13 methylation sites within their upstream regions. Of the 15 genes regulated by ModA13 listed in Table 2, six (represented by the black arrows) were found to have a ModA13 methylation site in the intergenic region upstream of the gene or operon. All methylation sites in these genomic regions are indicated with their FA1090 genome coordinates based on the genome sequence AE004969.1. Orientation of these non-palindromic sites is indicated by label position: sites in the sense orientation are labelled above the sequence, while those in the antisense orientation are labelled below.
(0.26 MB PDF)

Figure S8 Comparison of *modA11* and *modA13* expression in iron-replete and -deplete media. (A) Quantitative RT-PCR of *modA13* and *modA11* expression. No difference in *modA13* expression was observed for *modA13* ON cells grown in iron replete compared to *modA13* ON cells grown in iron- deplete media (P = 0.241), confirming that Mod is not regulated by iron. *modA11* expression was observed to be 2.4-fold higher in *modA11* ON cells grown in iron replete compared to *modA13* ON cells grown in iron-deplete media (P = 0.007). P-values were calculated using a Student's *t*-test. (B) Chromosomal DNA extracted from *N. gonorrhoeae* strains FA1090 *modA13* ON, *modA13* OFF, *modA13::kan* cells, grown in iron-replete and iron-deplete media, digested with ApoI and probed with a PCR product containing an ApoI/AGAAA overlap. The same pattern of digestion inhibition was observed for *modA13* ON cells grown in iron-replete and iron-deplete media. No differences in the digestion patterns were observed when comparing the *modA13* OFF and *modA13::kan* cells grown in iron-replete media to *modA13* OFF and *modA13::kan* cells grown in iron-deplete media, confirming that *mod* is not regulated by iron. (C) Analysis of Mod expression for MC58 *modA11* ON iron replete and MC58 *modA11* ON iron deplete. A Mod specific antibody was used to assess expression of Mod, as the *modA11* site is unknown, an analysis similar to (B), cannot be conducted. The positions of molecular weight standard proteins are shown on the right in kilo Daltons (kDa). The left panel shows coomasie stained MC58 *modA11* ON iron-replete and -deplete whole cells to show equal loadings of cell extracts. The right panel shows the Western blot of MC58 *modA11* ON iron-replete and -deplete whole cells whole cells probed with a Mod specific antibody. No difference in expression was observed between the *modA11* ON iron-replete and *modA11* ON -deplete cell extracts.
(0.94 MB PDF)

Table S1 *Mod* alleles and repeat numbers for *N. meningitidis* isolate strains. ^a^Genome strains. ^b^Number and expression state of repeats within the *ModA11* or *ModB1* gene; in-frame (ON) or out-of-frame (OFF). ^c^Repeats can be either CCCAA, GCCAA, or TCCAA. ^d^A strain was defined as having the *modA11* allele if the DNA recognition region was ≥95% identical at the nucleotide level to *modA11* gene of *N. meningitidis* strain MC58 (NMB1375; see Figure 1). A strain was defined as having the *modB2* allele if the DNA recognition region was ≥95% identical at the nucleotide level to *modB2* gene of *N. meningitidis* strain Z2491 (NMA1467; see Figure 1). ^e^Strain defined as having *modA4* or *modA15* allele as defined in Fox et al. 2007 [17]. ^f^Strain has a new allele henceforth defined as *modA18* in this paper. Shares similarity to *H. influenzae* strain 2019. ^g^ *modB1* strains that contain a premature stop codon. ^h^Frame shift mutation in *res* at nucleotide 2093. ND, not determined. Refer to Figure 1 and to the text.
(0.11 MB PDF)

Table S2 Mod alleles and repeat numbers for *N. gonorrhoeae* clinical isolate strains. ^a^DGI, disseminated gonococcal infection clinical isolates; MI, asymptomatic carriage or mucosal gonorrhoeae infection clinical isolates; UG, uncomplicated gonorrhoeae. ^b^Number and expression state of repeats within the mod gene; in-frame (ON) or out-of-frame (OFF). ^c^A strain was defined as having the *modA13* allele if the DNA recognition region was ≥95% identical at the nucleotide level to the *modA13* gene of *N. gonorrhoeae* strain FA1090 (NGO0641), and as *modA12* allele if the DNA recognition region was ≥95% identical at the nucleotide level to the *modA12* gene of *N. meningitidis* strain Z2491 (NMA1589/90). A strain was defined as having the *modB1* allele if the DNA recognition region was ≥95% identical at the nucleotide level to the *modB1* gene of *N. gonorrhoeae* strain FA1090 (NGO0545), see Figure 1. NA- *modB* gene not present. ^d^ 750 bp in-frame deletion in res. ND, not determined. Refer to Figure 1 and to the text.
(0.07 MB PDF)

Table S3 Differentially expressed genes in *N. meningitidis* wild-type MC58 *modA11* ON versus the MC58 *modA11::kan* mutant. The genes listed are either downregulated or upregulated in the *N. meningitidis* MC58 *modA11::kan* mutant strain. The identity of the gene is indicated with the gene ID in the annotation of the *N. meningitidis* strain MC58 genome (TIGR). The average ratio presented is the mean of MC58 *modA11::kan* mutant: wild-type MC58 *modA11* ON from six replicate spots on three independent microarrays, incorporating a dye swap. Only those genes with an expression value above 1.5-fold were included in this study except for NMB0205 and NMB2091, which are shown in italics. *Genes have been shown to be Fur regulated [55],[56].
(0.16 MB PDF)

Table S4 Differentially expressed genes in *N. meningitidis* wild-type B6116/77 *modA12* ON versus the mutant strain B6616/77 *modA12::kan*. The genes listed are either downregulated or upregulated in the *N. meningitidis* B6116/77 *modA12::kan* mutant strain. The identity of the gene is indicated with the gene ID in the annotation of the *N. meningitidis* strain MC58 and Z2491 genome (TIGR). The average ratio presented is the mean of B6116/77 *modA12::kan* mutant:wild-type B6116/77 *modA12* ON from six replicate spots on seven independent microarrays, incorporating a dye swap. Only those genes with an expression value above 1.5-fold were included in this study except for NMA1581and NMB1206, which are shown in italics.
(0.11 MB PDF)

Table S5 Differentially expressed genes in *N. gonorrhoeae* wild-type FA1090 *modA13* ON versus the mutant strain FA1090 *modA13::kan*. The genes listed are either downregulated or upregulated in the *N. gonorrhoeae* FA1090 *modA13::kan* mutant strain. The identity of the gene is indicated with the gene ID in the annotation of the *N. gonorrhoeae* genome (TIGR). The average ratio presented is the mean of FA1090 *modA13::kan* mutant:wild-type FA1090 *modA13* ON from six replicate spots on three independent microarrays, incorporating a dye swap. Only those genes with an expression value above 1.5-fold were included in this study. *Genes have been shown to be Fur regulated [56].
(0.12 MB PDF)

Table S6 Differentially expressed genes in *N. gonorrhoeae* wild-type 96D551 *modA12* ON versus the mutant strain 96D551 *modA12::kan*. The genes listed are either downregulated or upregulated in the *N. gonorrhoeae* 96D551 *modA12::kan* mutant strain. The identity of the gene is indicated with the gene ID in the annotation of the *N. gonorrhoeae* genome (TIGR). The average ratio presented is the mean of 96D551 *modA12::kan* mutant:wild-type 96D551 *modA12* ON from six replicate spots on three independent microarrays, incorporating a dye swap. Only those genes with an expression value above 1.5-fold were included in this study.
(0.08 MB PDF)

Table S7 Primers used to synthesize probes for Southern analysis, *mod* allele study, and sequencing plasmid pCmGFP. ^a^QRT-PCR primers used for *N. meningitidismodA11* study. ^b^QRT-PCR Primers used for *N. meningitidismodA12* and *N. gonorrhoeaemodA12* study. ^c^QRT-PCR primers used for *N. gonorrhoeaemodA13* study. QRT-PCR primers are named after their TIGR gene ID.
(0.06 MB PDF)

Table S8 Fragment analysis on FA1090 *modA13* ON/OFF original inoculum and 40, 50, and 60 ug/ml Triton X-100 concentrations (A) and FA1090 *modA13* ON/OFF ratio Student's *t*-test results (B). Data represents genescan analysis results where the size of the repeat tract was determined using fluorescent primers (see Materials and Methods) and contains values determined from three independent samples[17].
(0.05 MB PDF)

Table S9 Fragment analysis of O1G1370 *modA13* ON/OFF original inoculum and survival +3 h (A) and O1G1370 *modA13* ON/OFF ratio student's *t*-test results (B). Data represents genescan analysis results where the size of the repeat tract was determined using fluorescent primers (see Materials and Methods) and contains values determined from three independent samples[17].
(0.05 MB PDF)
