# Supplementary figures and images for: Phasevarions Mediate Random Switching of Gene Expression in Pathogenic Neisseria
Source: PLoS Pathog. 2009 Apr 24;5(4):e1000400. doi: 10.1371/journal.ppat.1000400 (PMC2667262; doi:10.1371/journal.ppat.1000400)

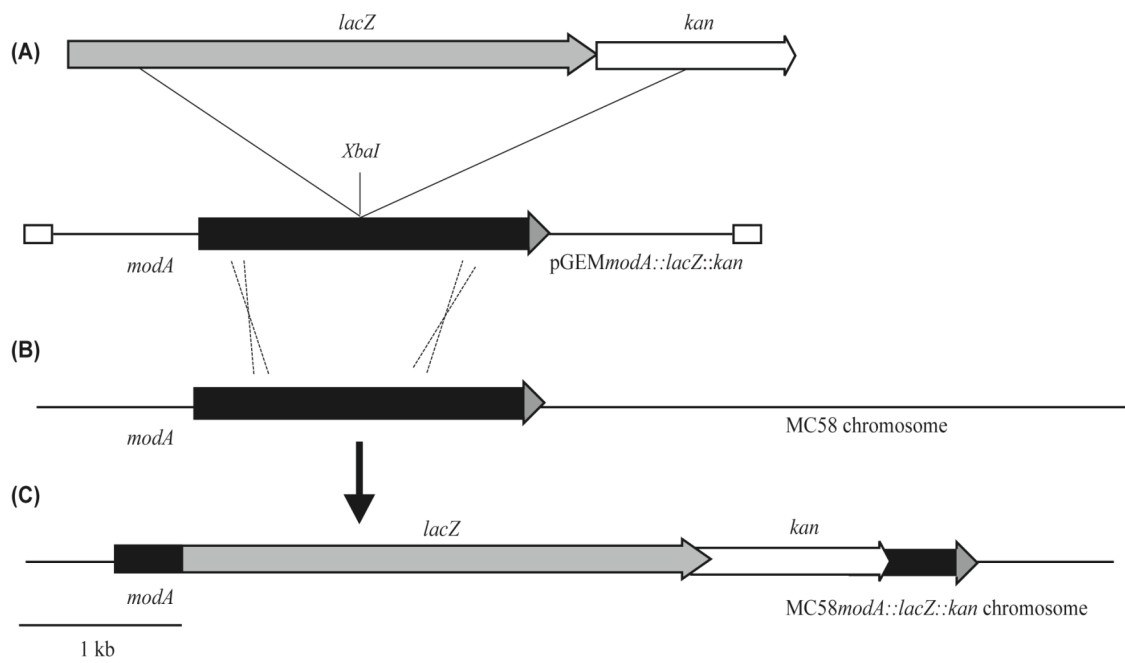

Supplement: Figure S1 — Schematic of the construction of pGEMmodA::lacZ::kan and subsequent transformation into N. meningitidis. (A) Insertion of the lacZ::kan cassette into the modA ORF. (B) Transformation into N. meningitidis strain MC58. (C) Double crossover event results in insertion of the plasmid into the MC58 chromosome resulting in strain MC58modA::lacZ::kan. (0.13 MB PDF) [file ppat.1000400.s001.pdf]

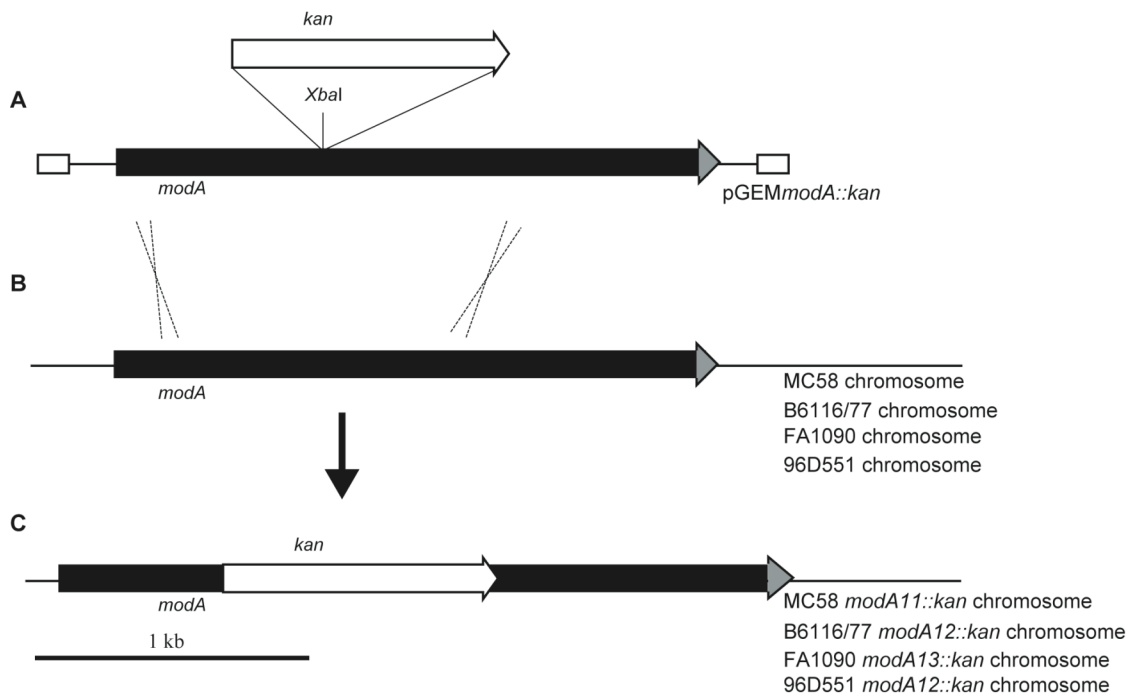

Supplement: Figure S2 — Schematic of the construction of pGEMmodA::kan and subsequent transformation into N. meningitidis or N. gonorrhoeae. (A) Insertion of the kanamycin (kan) cassette into the mod ORF. (B) Transformation into N. meningitidis strain MC58, N. meningitidis strain B6116/77, N. gonorrhoeae strain FA1090, or N. gonorrhoeae strain 96D551. (C) A double crossover event results in insertion of the plasmid; into the MC58 chromosome resulting in MC58 modA11::kan mutants, into the B6116/77 chromosome resulting in B6116/77 modA12::kan mutants, into the FA1090 chromosome resulting in FA1090 modA13::kan mutants, and into the 96D551 chromosome resulting in 96D551 modA12::kan mutants. (0.18 MB PDF) [file ppat.1000400.s002.pdf]

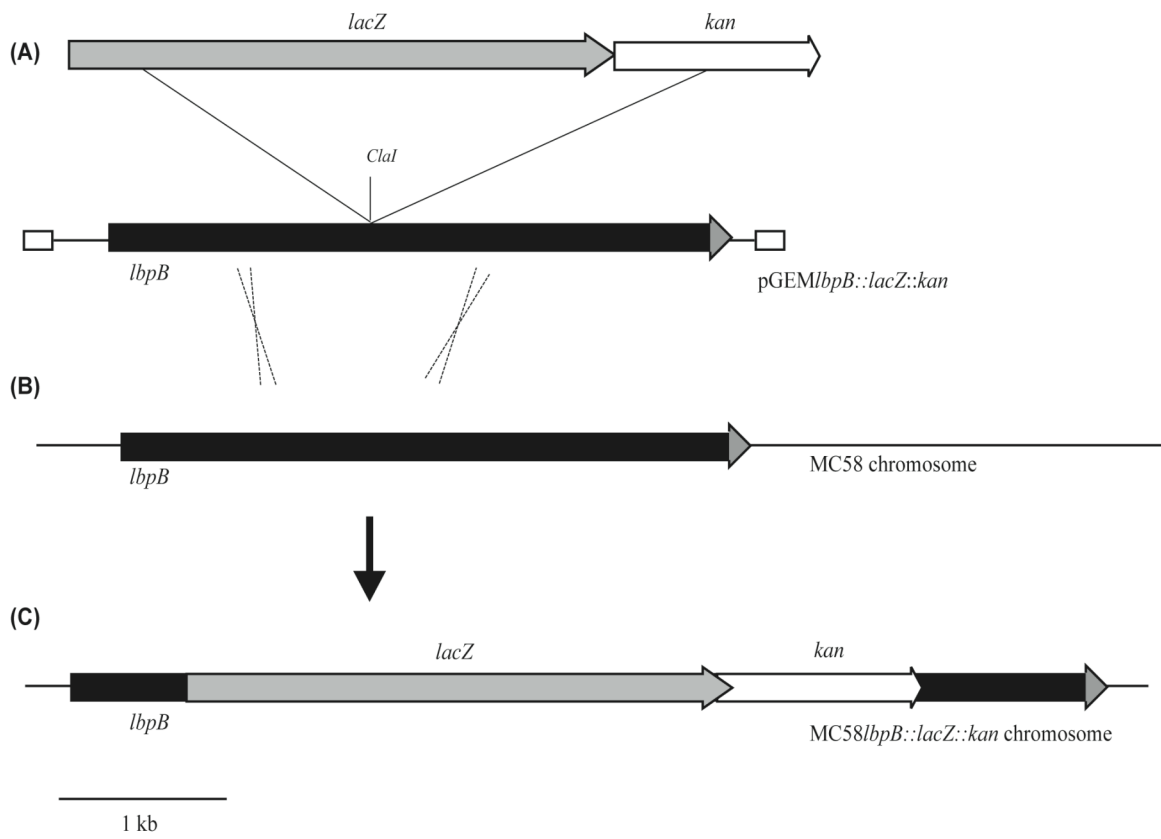

Supplement: Figure S3 — Schematic representation of the construction of pGEMlbpB::lacZ::kan and subsequent transformation into N. meningitidis. (A) Insertion of the lacZ::kan cassette into the lbpB ORF. (B) Transformation into N. meningitidis strain MC58 with a naturally derived number of modA11 OFF repeats and N. meningitidis strain MC58 with a naturally derived number of modA11 ON repeats. (C) Double crossover event results in insertion of the plasmid into the MC58 modA11 OFF and MC58 modA11 ON chromosome resulting in strains MC58lbpB::lacZ::kan modA11 OFF and MC58lbpB::lacZ::kan modA11 ON. (0.14 MB PDF) [file ppat.1000400.s003.pdf]

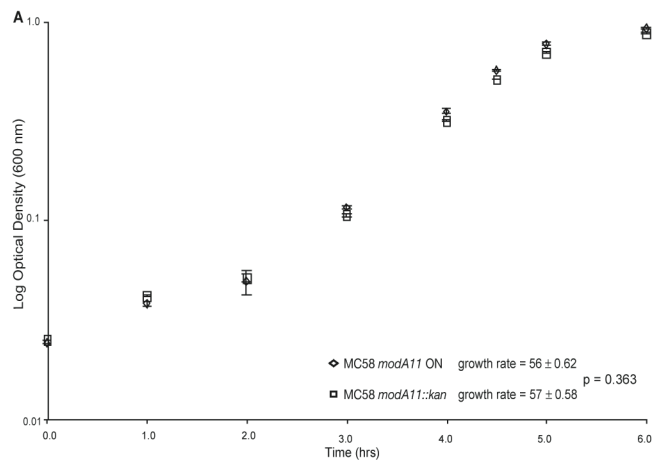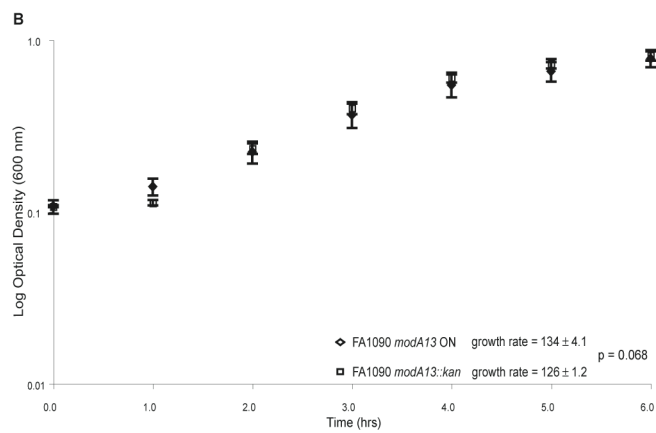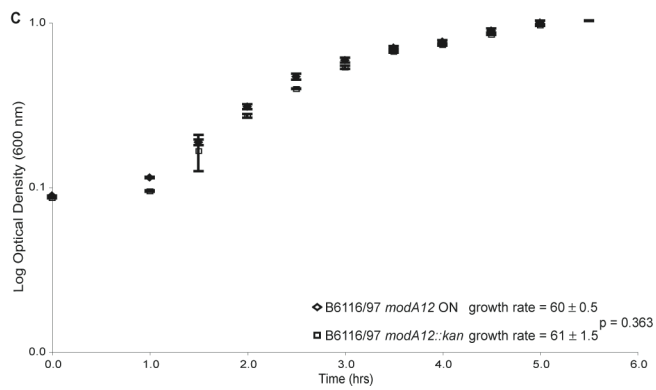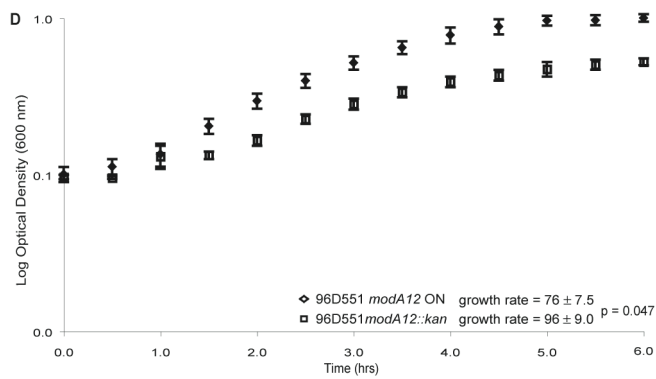

Supplement: Figure S4 — Growth rate comparisons of MC58 modA11 ON and MC58modA11::kan, FA1090 modA13 ON and FA10908 modA13::kan, B6116/77 modA12 ON and B6116/77 modA12::kan, and 96D551 modA12 ON and 96D551 modA12::kan. The optical density of wild-type and mutant cells, grown under the same iron-limiting conditions as used for expression and functional studies (see Materials and Methods), was measured and the differences in growth rate compared. The generation time was calculated from the slope of the line obtained in the logarithmic plot of exponential growth for each set of wild-type and mutant triplicates. The growth rate (minutes) was determined by 1/generation time. No significant difference in growth rate was observed between (A) MC58 modA11 ON and the MC58 modA11::kan mutant (P = 0.393), (B) FA1090 modA13 ON and FA10908 modA13::kan (P = 0.068), (C) B6116/77modA12 ON and B6116/77modA12::kan (P = 0.363). However, a significant difference in growth rate was observed between 96D551 modA12 ON and 96D551 modA12::kan (P = 0.047). P-values were calculated using a Student's t-test. (0.14 MB PDF) [file ppat.1000400.s004.pdf]

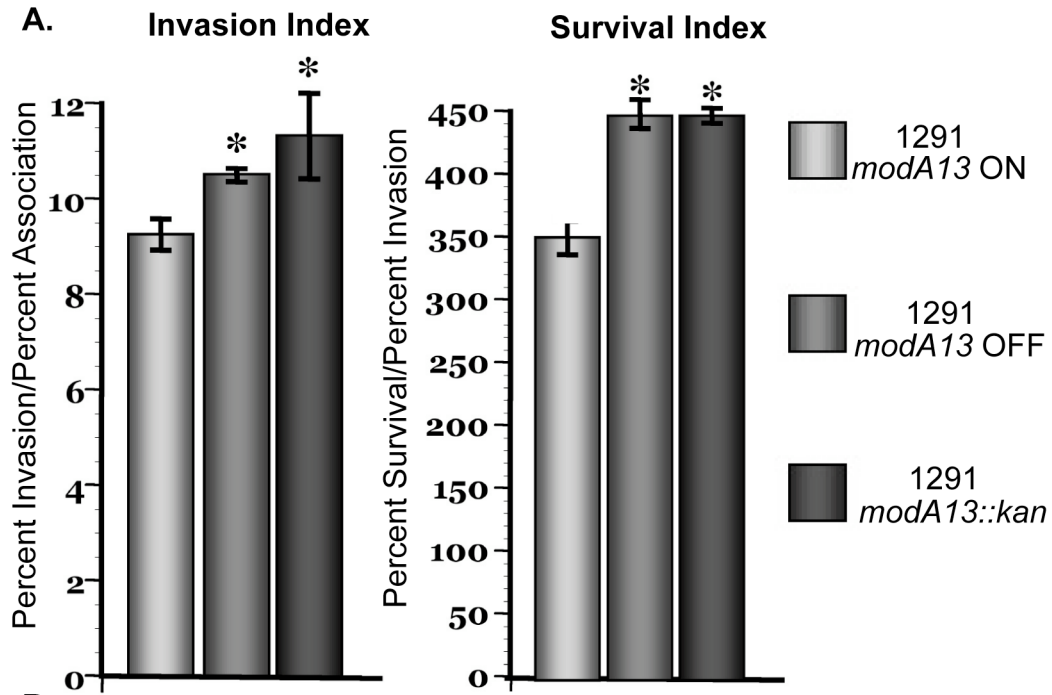

**B.**

| 1291              |       | Ratio ON/OFF |                    |                    |
|-------------------|-------|--------------|--------------------|--------------------|
|                   |       | Inoculum     | Invasion           | Survival           |
| <i>modA13</i> ON  | % ON  | 93.01        | 65.18              | 75.82              |
|                   | % OFF | 6.99         | 34.82 <sup>†</sup> | 24.18 <sup>†</sup> |
| <i>modA13</i> OFF | % ON  | 5.03         | 5.82               | 5.78               |
|                   | % OFF | 94.97        | 94.18              | 94.22              |

Supplement: Figure S6 — N. gonorrhoeae 1291 association with, and intracellular survival within, primary human cervical epithelial (pex) cells. Pex cells were challenged with N. gonorrhoeae strain 1291 as outlined in the main text. Data shown represent the invasion index (left panel) or the survival index (right panel) following challenge of pex cells as outlined in the main text. The invasion index represents the percentage of pex cell-associated gonococci that survive gentamicin treatment; whereas the survival index is the percentage of invasive gonococci that survive, intracellularly, within pex cells at 3 h post-invasion. There was no significant difference between the naturally occurring 1291 modA13 OFF isolate and the 1291 modA13::kan “knockout” strain in either the invasion (P = 0.254) or survival (P = 0.806) indices observed. A statistically significant difference (*) was obtained in the invasion (P = 0.008) and survival (P = 0.001) indices when comparing 1291 modA13 OFF to 1291 modA13 ON, and in the invasion (P = 0.037) and survival (P = 0.001) indices when comparing 1291 modA13::kan to 1291 modA13 ON. P values were determined using a Student's t-test. (B) Shows the ratio of 1291 modA13 ON to 1291 modA13 OFF of the inoculum, and at the invasion and survival time points for 1291 modA13 ON and 1291 modA13 OFF. †A statistically significant difference was seen in the ON/OFF ratio between the 1291 modA13 OFF inoculum sample and the 1291 modA13 OFF invasion sample (P = 0.0082) and the 1291 modA13 OFF inoculum sample and the 1291 modA13 OFF survival sample (P = 0.0333), indicating a selection for OFF organisms over the course of the 3-h assay. (0.34 MB PDF) [file ppat.1000400.s006.pdf]

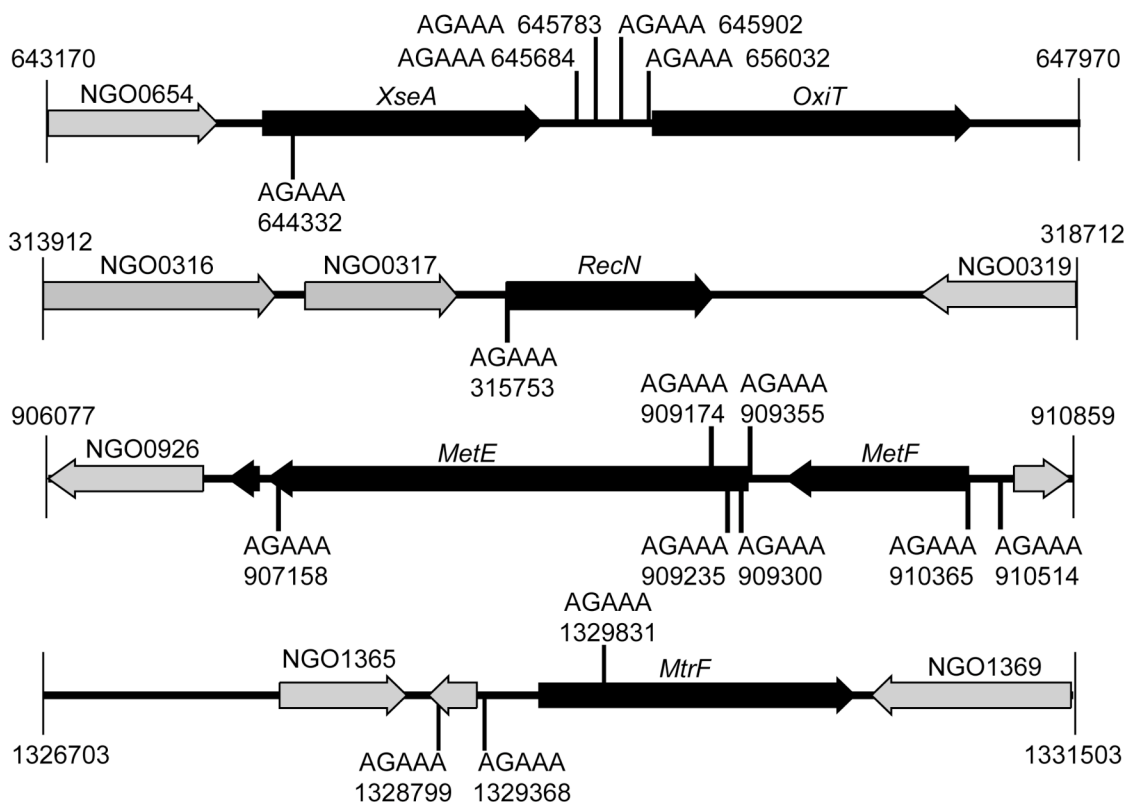

Supplement: Figure S7 — Genes regulated by ModA13 in FA1090 containing ModA13 methylation sites within their upstream regions. Of the 15 genes regulated by ModA13 listed in Table 2, six (represented by the black arrows) were found to have a ModA13 methylation site in the intergenic region upstream of the gene or operon. All methylation sites in these genomic regions are indicated with their FA1090 genome coordinates based on the genome sequence AE004969.1. Orientation of these non-palindromic sites is indicated by label position: sites in the sense orientation are labelled above the sequence, while those in the antisense orientation are labelled below. (0.26 MB PDF) [file ppat.1000400.s007.pdf]

**A** QRT-PCR of *modA13* and *modA11*

|                  | Fold difference (Iron Replete / Iron Deplete) | P-value |
|------------------|-----------------------------------------------|---------|
| <i>modA13</i> ON | 1.0                                           | 0.241   |
| <i>modA11</i> ON | 2.4                                           | 0.007   |

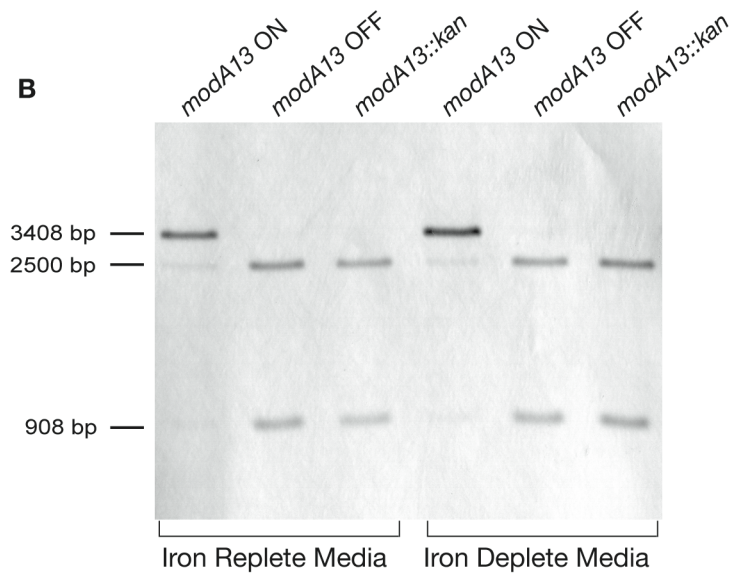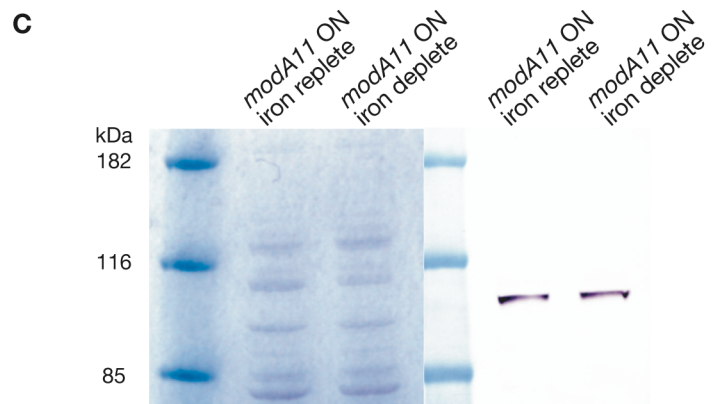

Supplement: Figure S8 — Comparison of modA11 and modA13 expression in iron-replete and -deplete media. (A) Quantitative RT-PCR of modA13 and modA11 expression. No difference in modA13 expression was observed for modA13 ON cells grown in iron replete compared to modA13 ON cells grown in iron- deplete media (P = 0.241), confirming that Mod is not regulated by iron. modA11 expression was observed to be 2.4-fold higher in modA11 ON cells grown in iron replete compared to modA13 ON cells grown in iron-deplete media (P = 0.007). P-values were calculated using a Student's t-test. (B) Chromosomal DNA extracted from N. gonorrhoeae strains FA1090 modA13 ON, modA13 OFF, modA13::kan cells, grown in iron-replete and iron-deplete media, digested with ApoI and probed with a PCR product containing an ApoI/AGAAA overlap. The same pattern of digestion inhibition was observed for modA13 ON cells grown in iron-replete and iron-deplete media. No differences in the digestion patterns were observed when comparing the modA13 OFF and modA13::kan cells grown in iron-replete media to modA13 OFF and modA13::kan cells grown in iron-deplete media, confirming that mod is not regulated by iron. (C) Analysis of Mod expression for MC58 modA11 ON iron replete and MC58 modA11 ON iron deplete. A Mod specific antibody was used to assess expression of Mod, as the modA11 site is unknown, an analysis similar to (B), cannot be conducted. The positions of molecular weight standard proteins are shown on the right in kilo Daltons (kDa). The left panel shows coomasie stained MC58 modA11 ON iron-replete and -deplete whole cells to show equal loadings of cell extracts. The right panel shows the Western blot of MC58 modA11 ON iron-replete and -deplete whole cells whole cells probed with a Mod specific antibody. No difference in expression was observed between the modA11 ON iron-replete and modA11 ON -deplete cell extracts. (0.94 MB PDF) [file ppat.1000400.s008.pdf]
