# Supplementary material for: Phasevarions Mediate Random Switching of Gene Expression in Pathogenic Neisseria
Source: PLoS Pathog. 2009 Apr 24;5(4):e1000400. doi: 10.1371/journal.ppat.1000400 (PMC2667262; doi:10.1371/journal.ppat.1000400)
Supplement: Figure S5 — Biofilm formation by N. gonorrhoeae strain FA1090 modA13::kan and wild-type FA1090 modA13 ON. The ability of wild-type FA1090 modA13 ON and N. gonorrhoeae FA1090 modA13::kan to form a biofilm was evaluated after two days of growth under continuous flow conditions. These experiments were performed in duplicate on three different occasions and representative images are shown. (A) Confocal microscopy of the biofilm mass over 2 days of growth for the N. gonorrhoeae wild-type FA1090 modA13 ON (1) and FA1090 modA13::kan mutant (2). These images are three-dimensional reconstructions of stacked z-series taken at 200× magnification, which were rendered by Volocity (see Materials and Methods). These images show that, overall, wild-type FA1090 modA13 ON formed a thinner and more diffuse biofilm with large gaps between biofilm clusters, while the FA1090 modA13::kan mutant formed a thicker and more densely packed biofilm with very few gaps occurring between biofilm clusters. (B) Scanning electron microscopy of the surface of the biofilm mass over 2 days of growth on glass taken at 5,000× magnification. The images show that FA1090 modA13::kan forms a biofilm that is tightly enmeshed in extracellular material that obscures the structure of individual cells. Cells in the FA1090 modA13 ON biofilm are clearly distinguishable and exhibit a normal blebbing phenotype. (C) Transmission electron microscopy of 70 nm thin-sections of the biofilm mass over 2 days of growth on glass taken at 10,000× magnification. The electron micrographs depicted are representative of images taken for modA13 ON and modA13::kan in two independent experiments. The images show that FA1090 modA13::kan forms a biofilm with a hyper-blebbing phenotype, as seen in the numerous enclosed membranes on the surface of the cells, while the FA1090 modA13 ON biofilm exhibit a wild-type blebbing phenotype with fewer blebs on the surface of the cells. The electron micrographs suggest that the extracellular matrix of the FA109 [file ppat.1000400.s005.pdf]

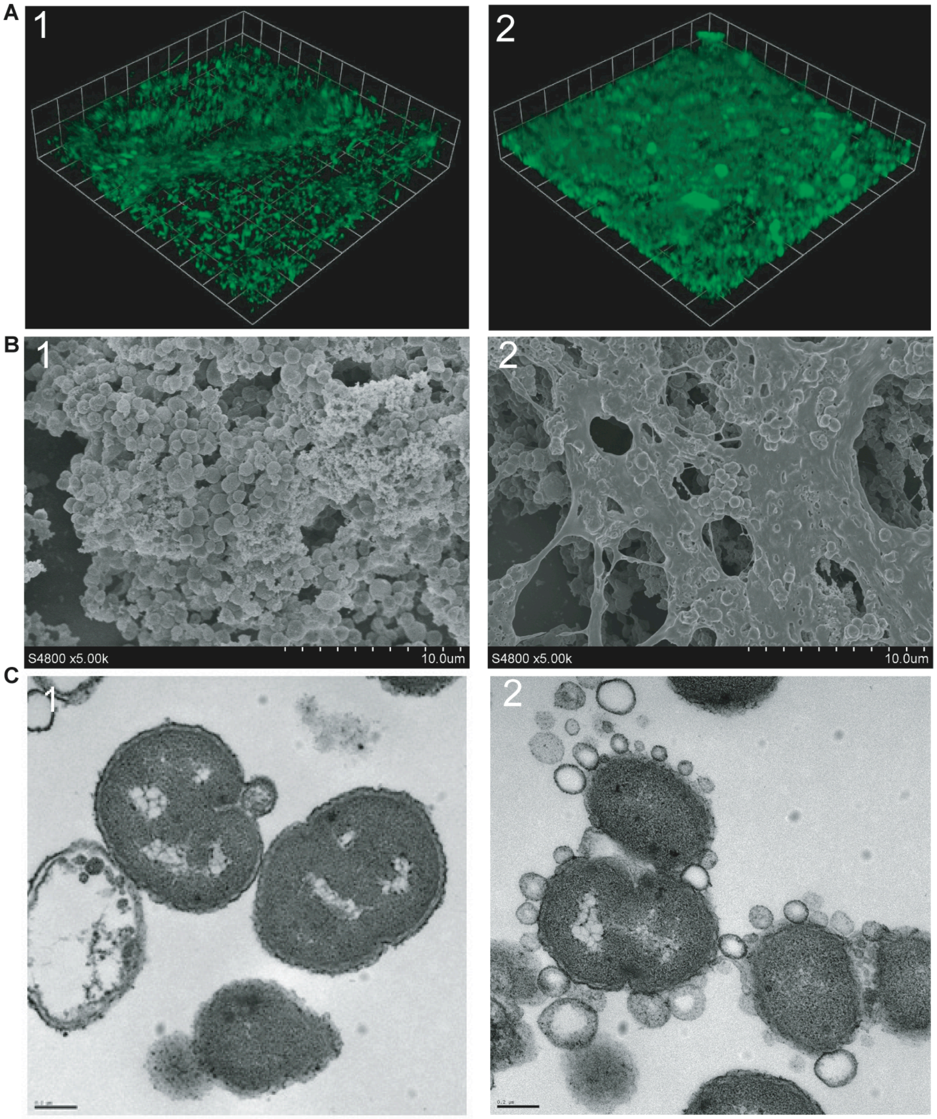

D

COMSTAT parameter	FA1090 *modA13* ON	FA1090 *modA13::kan*	P-value
Biomass ($\mu M^3/\mu M^2$)	5.21±2.2	23.9±7.0	0.0002
Average Thickness (μM)	31.7±8.9	63.8±8.2	0.0003
Maximum Thickness (μM)	89.0±22.4	74.7±10.9	0.1564
