# Supplementary material for: Phasevarions Mediate Random Switching of Gene Expression in Pathogenic Neisseria
Source: PLoS Pathog. 2009 Apr 24;5(4):e1000400. doi: 10.1371/journal.ppat.1000400 (PMC2667262; doi:10.1371/journal.ppat.1000400)
Supplement: Table S1 — Mod alleles and repeat numbers for N. meningitidis isolate strains. aGenome strains. bNumber and expression state of repeats within the ModA11 or ModB1 gene; in-frame (ON) or out-of-frame (OFF). cRepeats can be either CCCAA, GCCAA, or TCCAA. dA strain was defined as having the modA11 allele if the DNA recognition region was ≥95% identical at the nucleotide level to modA11 gene of N. meningitidis strain MC58 (NMB1375; see Figure 1). A strain was defined as having the modB2 allele if the DNA recognition region was ≥95% identical at the nucleotide level to modB2 gene of N. meningitidis strain Z2491 (NMA1467; see Figure 1). eStrain defined as having modA4 or modA15 allele as defined in Fox et al. 2007 [17]. fStrain has a new allele henceforth defined as modA18 in this paper. Shares similarity to H. influenzae strain 2019. g modB1 strains that contain a premature stop codon. hFrame shift mutation in res at nucleotide 2093. ND, not determined. Refer to Figure 1 and to the text. (0.11 MB PDF) [file ppat.1000400.s009.pdf]

Table S1. *Mod* alleles and repeat numbers for *N. meningitidis* isolate strains

Strain	ST	*modA* allele [d]	Repeats AGCC [b]	ON/OFF [b]	*modB* allele [d]	Repeats CCCAA [b,c]	ON/OFF [b]
MC58 [a]	32	*modA11* [h]	20	OFF	*modB1* [g]	16	OFF
Z2491 [a]	4	*modA12*	3	OFF	*modB2*	8	OFF
153	5	*modA12*	4	ON	ND		
F6124	5	*modA12*	23	OFF	ND		
44/76	32	*modA11*	18	OFF	*modB1* [g]	ND	
14/1455	5	*modA12*	4	ON	*modB2*	11	OFF
F4698	5	*modA12*	3	OFF	*modB2*	13	OFF
3906	17	*ModA12*	3	OFF	ND		
NG PB24	32	*modA11* [h]	29	OFF	*modB1*	10	OFF
BZ 169	32	*modA11* [h]	23	OFF	*modB1* [g]	10	OFF
500	11	*modA12*	3	OFF	ND		
BZ 10	8	*modA11*	8 AGTC	-	ND		
BZ198	41	*modA12*	3	OFF	*modB2*	5	OFF
NG P20	11	*modA12*	3	OFF	*modB2*	7	
297-0	49	*modA15* [e]	27	OFF	*modB2*	9 TCCAA	ON
1000	20	*modA12*	3	OFF	ND		
8680	32	*modA15* [e]	27	OFF	ND		
AK50	41	*modA12*	10	ON	ND		
860060	24	*modA12*	3	OFF	ND		
B6116/77	10	*modA12*	24	OFF	ND		
M40/94	41	*modA12*	10	ON	*modB2*	17 GCCAA	OFF
M-101/93	41	*modA12*	13	ON	*modB2*	6 GCCAA/CCCAA	ON
312 901	8	*modA12*	10	ON	ND		
890326	28	*modA12*	7	ON	ND		
88/03415	46	*modA12*	16	ON	*modB2*	9	ON
G2136	8	*modA12*	19	ON	ND		
SB25	8	*modA12*	15	OFF	*modB2*	8	OFF
2059001	4	*modA12*	3	OFF	ND		
A22	22	*modA12*	3	OFF	*modB2*	13	OFF
E26	39	*modA12*	11	OFF	ND		
N45/96	41	*modA11*	32	OFF	ND		
E32	29	*modA12*	24	OFF	ND		
204/92	31	*modA11*	27	OFF	ND		
94/155	23	*modA12*	10	ON	ND		
50/94	33	*modA12*	16	ON	ND		
400	66	*modA12*	15	OFF	*modB2*	6 GCCAA	ON
931905	45	*modA12*	23	OFF	ND		
91/40	40	*modA12*	22	ON	ND		
AK22	41	*modA12*	10	ON	*modB2*	16 GCCAA	OFF
L93/4286	42	*modA12*	15	OFF	ND		
7891	8	*modA12*	3	OFF	*modB2*	10	OFF
D8	11	*modA12*	3	OFF	*modB2*	8	OFF
C751	5	*modA12*	3	OFF	ND		

26	12	*modA12*	3	OFF	ND		
S5611	4	*modA12*	3	OFF	ND		
154	4	*modA12*	3	OFF	*modB2*	9	ON
20	4	*modA12*	3	OFF	*modB2*	9	ON
CN100	1	*modA12*	3	OFF	*modB2*	3	ON
371	6	*modA12*	3	OFF	ND		
DK 353	1	*modA11*	16	ON	*modB1*	10	OFF
NG 6/88	21	*modA11*	29	OFF	ND		
EG 328	1	*modA12*	29	OFF	ND		
NG G40	37	*modA11*	16	ON	*modB1*	9 GCCAA	
BZ 147	13	*modA12*	7	ON	*modB1*	4	OFF
528	18	*modA12*	9	OFF	ND		
EG 011	25	*modA11*	11	OFF	ND		
79126	48	*modA12*	3	OFF	ND		
NG 080	18	*modA11* [h]	24	OFF	*modB1* [g]	ND	
243	36	*modA12*	3	OFF	ND		
MA-5756	3	*modA12*	11	OFF	ND		
M597	32	*modA12*	19	ON	ND		
BRAZ10	4	*modA12*	3	OFF	*modB2*	7	OFF
92001	11	*modA12*	10	ON	*modB2*	8	
6748	11	*modA12*	2	OFF	*modB2*	3	
D1	11	*modA12*	19	ON	ND		
90/18311	7	*modA12*	11	OFF	ND		
BZ 163	1	*modA12*	22	ON	*modB2*	ND	
NG H41	11	*modA12*	12	OFF	*modB2*	16	OFF
NG F26	11	*modA11*	17	OFF	*modB1*	9	ON
NG E28	9	*modA18* [f]	18	OFF	ND		
80049	27	*modA12*	24	OFF	*modB2*	7	OFF
NG H36	14	*modA12*	23	OFF	*modB1*	ND	
139M	26	*modA12*	3	OFF	*modB2*	4	OFF
H1964	5	*modA12*	3	OFF	ND		
SWZ107	47	*modA11*	11	OFF	*modB1*	11	OFF
EG 327	1	*modA12*	12	OFF	*modB1*	38 GCCAA	OFF
NG H15	5	*modA12*	26	OFF	*modB2*	8	OFF
79128	35	*modA12*	3	OFF	ND		
BZ 232	19	*ModA4* [e]	15	OFF	*modB1*	6 GCCAA	ON
DK 24	43	*modA12*	11	OFF	ND		
A4/M1027	3	*modA12*	3	OFF	ND		
NG E30	38	*modA12*	20	OFF	*modB1*	7 GCCAA	OFF
690	16	*modA12*	4	ON	ND		
S4355	4	*modA12*	3	OFF	*modB2*	10	OFF
38VI	44	*modA12*	3	OFF	ND		
106	4	*modA12*	3	OFF	*modB2*	3	OFF
196/87	5	*modA11*	24	OFF	ND		
322/85	11	*modA12*	3	OFF	ND		
255	1	*modA12*	3	OFF	ND		

254	32	*modA12*	3	OFF	*modB2*	8	OFF
S3131	2	*modA12*	3	OFF	ND		
NG E31	4	*modA12*	3	OFF	ND		
NG 4/88	1	*modA12*	24	OFF	*modB2*	6	ON
IAL2229	4	*modA12*	3	OFF	*ND*		
NG H38	15	*modA11*	11	OFF	*modB2*	17 GCCAA	OFF
EG 329	30	*modA11* [h]	26	OFF	*modB1* [g]	ND	
NG144/82	5	*modA11* [h]	18	OFF	*modB1* [g]	ND	
BZ 83	36	*modA11* [h]	18	OFF	*modB1*	4	OFF
1014	32	*modA12*	3	OFF	ND		
120M	32	*modA12*	3	OFF	ND		
393	34	*modA12*	3	OFF	ND		
BZ 133	4	*modA12*	3	OFF	*modB1*	3	OFF
10	1	*modA12*	3	OFF	ND		
11-004	1	*modA12*	3	OFF	ND		
F1576	1	*modA12*	3	OFF	ND		
129E	4	*modA12*	3	OFF	ND		
NG 3/88	12	*modA12*	14	OFF	*modB1* [g]	4	OFF

[a] Genome strains
[b] Number and expression state of repeats within the *ModA11* or *ModB1* gene; in-frame (ON) or out-of-frame (OFF)
[c] Repeats can be either CCCAA, GCCAA or TCCAA
[d] A strain was defined as having the *modA11* allele if the DNA recognition region was ≥ 95% identical at the nucleotide level to *modA11* gene of *N. meningitidis* strain MC58 (NMB1375; see Figure 1). A strain was defined as having the *modB2* allele if the DNA recognition region was ≥ 95% identical at the nucleotide level to *modB2* gene of *N. meningitidis* strain Z2491 (NMA1467; see Figure 1)
[e] Strain defined as having *modA4* or *modA15* allele as defined in Fox *et al*. 2007 [17]
[f] Strain has a new allele henceforth defined as *modA18* in this paper. Shares similarity to *H. influenzae* strain 2019
[g] *modB1* strains that contain a premature stop codon
[h] Frame shift mutation in *res* at nucleotide 2093
ND: not determined
Refer to Figure 1 and to the text.
