# Supplementary material for: Phasevarions Mediate Random Switching of Gene Expression in Pathogenic Neisseria
Source: PLoS Pathog. 2009 Apr 24;5(4):e1000400. doi: 10.1371/journal.ppat.1000400 (PMC2667262; doi:10.1371/journal.ppat.1000400)
Supplement: Table S2 — Mod alleles and repeat numbers for N. gonorrhoeae clinical isolate strains. aDGI, disseminated gonococcal infection clinical isolates; MI, asymptomatic carriage or mucosal gonorrhoeae infection clinical isolates; UG, uncomplicated gonorrhoeae. bNumber and expression state of repeats within the mod gene; in-frame (ON) or out-of-frame (OFF). cA strain was defined as having the modA13 allele if the DNA recognition region was ≥95% identical at the nucleotide level to the modA13 gene of N. gonorrhoeae strain FA1090 (NGO0641), and as modA12 allele if the DNA recognition region was ≥95% identical at the nucleotide level to the modA12 gene of N. meningitidis strain Z2491 (NMA1589/90). A strain was defined as having the modB1 allele if the DNA recognition region was ≥95% identical at the nucleotide level to the modB1 gene of N. gonorrhoeae strain FA1090 (NGO0545), see Figure 1. NA- modB gene not present. d 750 bp in-frame deletion in res. ND, not determined. Refer to Figure 1 and to the text. (0.07 MB PDF) [file ppat.1000400.s010.pdf]

Table S2. ***Mod*** **alleles and repeat numbers for** ***N. gonorrhoeae*** **clinical isolate strains**

**Strain**	**Pathology**[a]	***modA allele***[c]	**Repeats AGCC**[b]	***modB allele***[c]	**Repeats CCCAA**[b]
FA1090	DGI	*modA13*	19 (ON)	*modB1*	12 (ON)
01G1052	MI	*modA13*	15 (OFF)	*modB1*	16 (OFF)
01G1370[d]	MI	*modA13*	13 (ON)	NA	NA
01G0957	DGI	*modA12*	13 (ON)	*modB1*	16 (OFF)
90/G747	DGI	*modA13*	17 (OFF)	*modB1*	8 (OFF)
94G163	MI	*modA13*	17 (OFF)	*modB1*	31 (OFF)
98G1131	DGI	*modA12*	20 (OFF)	*modB1*	20 (OFF)
00G0794 [d]	MI	*modA12*	33 (OFF)	*modB1*	8 (OFF)
01D064	MI	*modA12*	17 (OFF)	*modB1*	21 (ON)
02D004	DGI	*modA12*	26 (OFF)	*modB1*	17(OFF)
02D156	DGI	*modA12*	15 (OFF)	*modB1*	2 (OFF)
02W001	DGI	*modA13*	10 (ON)	*modB1*	19 (OFF)
98D159	DGI	*modA12*	27 (OFF)	*modB1*	16 (OFF)
97D040	DGI	*modA12*	20 (OFF)	*modB1*	12 (ON)
97D059	MI	*modA12*	24 (OFF)	*modB1*	2 (OFF)
96D551	MI	*modA12*	25 (ON)	*modB1*	16 (OFF)
1291	UG	*modA13*	15 (OFF)	*modB1*	7 (OFF)
MS11	UG	*modA13*	26 (OFF)	*modB1*	8 (OFF)

[a] DGI: disseminated gonococcal infection clinical isolates, MI: asymptomatic carriage or mucosal gonorrhoeae infection clinical isolates, UG: uncomplicated gonorrhoeae
[b] Number and expression state of repeats within the *mod* gene; in-frame (ON) or out-of-frame (OFF)
[c] A strain was defined as having the *modA13* allele if the DNA recognition region was ≥ 95% identical at the nucleotide level to the *modA13* gene of *N. gonorrhoeae* strain FA1090 (NGO0641), and as *modA12* allele if the DNA recognition region was ≥ 95% identical at the nucleotide level to the *modA12* gene of *N. meningitidis* strain Z2491 (NMA1589/90). A strain was defined as having the *modB1* allele if the DNA recognition region was ≥ 95% identical at the nucleotide level to the *modB1* gene of *N. gonorrhoeae* strain FA1090 (NGO0545), see Figure 1.
NA- *modB* gene not present,
[d] 750 bp in-frame deletion in *res*
ND: not determined
Refer to Figure 1 and to the text
