# Supplementary material for: Phasevarions Mediate Random Switching of Gene Expression in Pathogenic Neisseria
Source: PLoS Pathog. 2009 Apr 24;5(4):e1000400. doi: 10.1371/journal.ppat.1000400 (PMC2667262; doi:10.1371/journal.ppat.1000400)
Supplement: Table S3 — Differentially expressed genes in N. meningitidis wild-type MC58 modA11 ON versus the MC58 modA11::kan mutant. The genes listed are either downregulated or upregulated in the N. meningitidis MC58 modA11::kan mutant strain. The identity of the gene is indicated with the gene ID in the annotation of the N. meningitidis strain MC58 genome (TIGR). The average ratio presented is the mean of MC58 modA11::kan mutant: wild-type MC58 modA11 ON from six replicate spots on three independent microarrays, incorporating a dye swap. Only those genes with an expression value above 1.5-fold were included in this study except for NMB0205 and NMB2091, which are shown in italics. *Genes have been shown to be Fur regulated [55],[56]. (0.16 MB PDF) [file ppat.1000400.s011.pdf]

Table S3. Differentially expressed genes in *N. meningitidis* wild-type MC58 *modA11* ON versus the MC58 *modA11::kan* mutant

Gene ID	Gene	Ratio	Qrt-pcr	B-stat
	**Reduced expression in *N. meningitidis modA11* mutant**			
NMB0010	phosphoglycerate kinase	0.41		3.12
NMB0011	UDP-N-acetylglucosamine 1-carboxyvinyltransferase	0.66		1.28
NMB0059	dnaJ protein	0.55		3.82
NMB0085	sodium/glutamate symporter	0.57		3.19
NMB0126	transcription antitermination protein NusG	0.52		2.61
NMB0129	hypothetical protein	0.53		4.34
NMB0133	DNA-directed RNA polymerase, beta subunit	0.41		4.72
NMB0143	50S ribosomal protein L4	0.49		2.82
NMB0144	50S ribosomal protein L23	0.38	0.58± 0.16	4.27
NMB0146	30S ribosomal protein S19	0.51		2.45
NMB0147	50S ribosomal protein L22	0.46		3.82
NMB0148	30S ribosomal protein S3	0.40	0.40± 0.06	5.44
NMB0152	50S ribosomal protein L14	0.49		4.12
NMB0155	30S ribosomal protein S14	0.43	0.57± 0.08	2.30
NMB0156	30S ribosomal protein S8	0.51		1.47
NMB0159	30s ribosomal protein S5	0.54		2.66
NMB0166	30S ribosomal protein S11	0.49		2.70
NMB0186	undecaprenyl pyrophosphate synthetase	0.67		1.56
NMB0249	NADH dehydrogenase I, G subunit	0.57		2.50
NMB0250	NADH dehydrogenase I, H subunit	0.57		2.79
NMB0251	NADH dehydrogenase I, I subunit*	0.50		4.27
NMB0252	hypothetical protein	0.46		4.51
NMB0253	NADH dehydrogenase I, J subunit	0.61		2.95
NMB0254	NADH dehydrogenase I, K subunit	0.66		1.77
NMB0257	NADH dehydrogenase I, L subunit	0.56		3.43
NMB0259	NADH dehydrogenase I, N subunit	0.55		3.26
NMB0260	hypothetical protein	0.59		1.64
NMB0266	conserved hypothetical protein	0.60		1.90
NMB0276	conserved hypothetical protein	0.62		2.67
NMB0277	virulence factor MviN	0.63		2.64
NMB0322	50S ribosomal protein L33	0.64		1.95
NMB0326	octaprenyl-diphosphate synthase	0.63		2.52
NMB0327	conserved hypothetical protein, authentic frameshift	0.60		1.96
NMB0340	lactoylglutathione lyase*	0.49		4.68
NMB0346	hypothetical protein	0.53		2.90
NMB0347	conserved hypothetical protein	0.60		1.94
NMB0378	phosphate permease, putative	0.43		4.96
NMB0408	bacitracin resistance protein	0.63		0.74
NMB0418	phospho-N-acetylmuramoyl-pentapeptide-transferase	0.62		1.65
NMB0419	conserved hypothetical protein	0.65		0.67
NMB0420	UDP-N-acetylmuramoylalanine-D-glutamate ligase	0.64		1.33
NMB0422	UDP-N-acetylglucosamine-N-acetylmuramyl transferase	0.61		2.81
NMB0423	UDP-N-acetylmuramate-alanine ligase	0.60		1.58
NMB0424	D-alanine--D-alanine ligase	0.51		3.52
NMB0426	cell division protein FtsA	0.66		1.85
NMB0465	conserved hypothetical protein	0.59		3.19
NMB0466	aspartyl-tRNA synthetase	0.57		3.11
NMB0472	8-amino-7-oxononanoate synthase	0.57		3.85
NMB0525	aluminum resistance protein, putative	0.64		2.20
NMB0529	conserved hypothetical protein	0.64		1.37
NMB0531	conserved hypothetical protein	0.54		3.50
NMB0535	glucose/galactose transporter	0.53		2.75
NMB0536	Na+/H+ antiporter	0.51		3.95
NMB0564	Na(+)-translocating NADH-quinone reductase, subunit F	0.50		3.87
NMB0565	Na(+)-translocating NADH-quinone reductase, subunit E	0.64		1.57
NMB0566	Na(+)-translocating NADH-quinone reductase, subunit D	0.56		2.50
NMB0567	Na(+)-translocating NADH-quinone reductase, subunit C	0.59		2.32
NMB0568	Na(+)-translocating NADH-quinone reductase, subunit B*	0.49		4.81
NMB0569	Na(+)-translocating NADH-quinone reductase, subunit A	0.61		3.12
NMB0589	50s ribosomal protein L19	0.59		3.48
NMB0596	hypothetical protein	0.53		4.61
NMB0599	conserved hypothetical protein	0.65		1.88
NMB0623	spermidine/putrescine ABC transporter, periplasmic binding protein	0.51		4.06
NMB0626	peptide chain release factor 3	0.56		2.46
NMB0663	outer membrane protein NspA	0.63		2.00

NMB0665	oxygen-independent coprophorphyrinogen III oxidase family protein	0.64	2.34
NMB0700	IgA-specific serine endopeptidase	0.57	3.97
NMB0702	competence protein ComA	0.67	1.63
NMB0710	hypothetical protein	0.64	2.15
NMB0718	ferrochelatase	0.58	3.33
NMB0720	threonyl-tRNA synthetase	0.66	2.08
NMB0729	integration host factor, alpha subunit	0.63	0.11
NMB0735	4-hydroxybenzoate octaprenyltransferase	0.66	0.86
NMB0758	polyribonucleotide nucleotidyltransferase	0.45	4.56
NMB0777	uroporphyrinogen-III synthase HemD, putative	0.65	0.41
NMB0780	hypothetical protein	0.63	1.53
NMB0788	amino acid ABC transporter, permease protein	0.60	3.14
NMB0789	amino acid ABC transporter, ATP-binding protein	0.61	2.97
NMB0800	conserved hypothetical protein	0.62	2.36
NMB0827	type II restriction enzyme-related protein, authentic frameshift	0.61	2.39
NMB0828	ADP-L-glycero-D-mannoheptose-6-epimerase	0.54	3.04
NMB0829	type I restriction enzyme EcoR124II M protein	0.62	1.40
NMB0850	hypothetical protein	0.64	0.98
NMB0875	ribose-phosphate pyrophosphokinase	0.54	3.21
NMB0878	threonine dehydratase	0.55	3.31
NMB0898	hypothetical protein	0.66	0.36
NMB0937	elongation factor P (EF-P)	0.45	4.08
NMB1036	3-isopropylmalate dehydratase, large subunit	0.62	1.94
NMB1037	glutamate-cysteine ligase	0.53	3.94
NMB1038	DNA repair protein RadC	0.64	0.69
NMB1070	2-isopropylmalate synthase	0.57	0.11
NMB1072	prolipoprotein diacylglyceryl transferase	0.65	1.84
NMB1128	conserved hypothetical protein	0.54	3.28
NMB1131	chaperone protein HscA	0.65	0.95
NMB1132	hypothetical protein	0.61	0.94
NMB1133	conserved hypothetical protein / ankyrin-related protein	0.60	3.07
NMB1134	ferredoxin, 2Fe-2S type	0.56	1.92
NMB1146	biotin synthetase	0.65	2.24
NMB1149	hypothetical protein	0.67	1.69
NMB1238	peptidyl-prolyl cis-trans isomerase-related protein	0.62	2.66
NMB1252	phosphoribosylformylglycinamidine cyclo-ligase	0.56	2.94
NMB1269	hypothetical protein	0.63	2.69
NMB1273	alginate O-acetylation protein AlgI, putative	0.61	0.68
NMB1288	ribonucleoside-diphosphate reductase, beta subunit	0.63	1.72
NMB1289	type II restriction enzyme, putative	0.61	1.38
NMB1298	ribosomal small subunit pseudouridine synthase A	0.61	2.98
NMB1301	30S ribosomal protein S1	0.58	2.86
NMB1302	integration host factor, beta subunit*	0.43	6.20
NMB1312	ATP-dependent Clp protease, proteolytic subunit	0.64	1.72
NMB1313	trigger factor	0.40	5.59
NMB1314	cell division protein FtsK	0.65	1.28
NMB1320	50S ribosomal protein L9	0.62	0.10
NMB1321	30S ribosomal protein S18	0.54	2.39
NMB1323	30S ribosomal protein S6	0.58	3.45
NMB1338	isomerase, putative	0.61	0.77
NMB1362	oxalate/formate antiporter, putative	0.62	1.64
NMB1368	ATP-dependent RNA helicase, putative	0.63	0.14
NMB1375	modification methylase, putative, authentic frameshift	0.63	0.95
NMB1394	4-hydroxy-2-oxoglutarate aldolase/2-dehydro-3-deoxyphosphogluconate aldolase	0.45	5.30
NMB1425	lysyl-tRNA synthetase, heat inducible	0.58	2.87
NMB1446	3-dehydroquinate dehydratase	0.65	0.79
NMB1457	transketolase	0.66	2.02
NMB1467	exopolyphosphatase	0.59	3.69
NMB1485	conserved hypothetical protein	0.57	2.27
NMB1487	fimbrial assembly protein	0.64	1.95
NMB1498	aspartokinase, alpha and beta subunits	0.63	2.66
NMB1506	arginyl-tRNA synthetase	0.59	2.45
NMB1555	long-chain-fatty-acid--CoA ligase	0.65	1.68
NMB1571	conserved hypothetical protein	0.61	2.74
NMB1573	ornithine carbamoyltransferase, catabolic	0.61	0.96
NMB1577	acetolactate synthase III, large subunit	0.58	1.60
NMB1621	glutathione peroxidase	0.51	3.25
NMB1642	N utilization substance protein A	0.65	1.91
NMB1643	translation initiation factor IF-2	0.60	3.37
NMB1658	DNA/pantothenate metabolism flavoprotein	0.62	0.90
NMB1664	protease, putative	0.63	2.06
NMB1669	iron-starvation protein PigA	0.51	4.88

NMB1670	PqiA family protein	0.59		2.68
NMB1671	pqiB protein	0.61		1.44
NMB1703	3-oxoacyl-(acyl-carrier-protein) synthase II	0.60		2.30
NMB1714	multidrug efflux pump channel protein MtrE	0.59		0.67
NMB1715	multiple transferable resistance system protein MtrD	0.61		2.04
NMB1729	biopolymer transport protein ExbB	0.49		5.00
NMB1804	cytochrome c-type biogenesis protein, putative	0.65		1.33
NMB1823	valine--pyruvate aminotransferase	0.59		3.20
NMB1851	hypothetical protein	0.66		1.78
NMB1861	acetyl-CoA carboxylase, biotin carboxylase	0.57		3.71
NMB1905	ribonuclease P protein component	0.66		0.95
NMB1929	lacto-N-neotetraose biosynthesis glycosyl transferase LgtA	0.56		3.97
NMB1932	glycyl-tRNA synthetase, alpha chain	0.59		2.85
NMB1937	ATP synthase F1, delta subunit	0.47		2.35
NMB1963	conserved hypothetical protein	0.62		2.30
NMB1976	diaminopimelate decarboxylase	0.64		1.78
NMB1996	phosphoribosylformylglycinamidine synthase	0.65		1.26
NMB2051	ubiquinol-cytochrome c reductase, cytochrome c1	0.58		3.03
NMB2052	ubiquinol-cytochrome c reductase, cytochrome b*	0.50		4.48
NMB2057	50S ribosomal protein L13	0.47		3.91
NMB2075	BirA protein/Bvg accessory factor	0.61		2.73
NMB2079	aspartate-semialdehyde dehydrogenase	0.56		4.21
NMB2133	sodium/dicarboxylate symporter family protein	0.44		3.55
NMB2151	phosphoribosylamine--glycine ligase	0.52		3.28
NMB2153	conserved hypothetical protein	0.53		3.29
**Increased expression in *N. meningitidis modA11* mutant**				
NMB0006	thioredoxin-related protein	2.58		3.48
NMB0014	3-deoxy-D-manno-octulosonic-acid transferase	2.97	2.46± 0.50	5.89
NMB0021	pilS cassette	1.97		3.16
NMB0029	glycerate dehydrogenase	1.74		1.56
NMB0031	glucosamine-fructose-6-phosphate aminotransferase	1.85		3.39
NMB0063	dTDP-D-glucose 4,6-dehydratase	2.16		3.48
NMB0064	UDP-glucose 4-epimerase	1.57		1.99
NMB0071	capsule polysaccharide export outer membrane protein CtrA	1.51		1.58
NMB0105	PhnO-related protein	1.60		1.33
NMB0110	polypeptide deformylase	3.12		7.09
NMB0122	conserved hypothetical protein	1.97		3.52
NMB0177	sodium/alanine symporter, putative	2.46		5.66
NMB0190	glucose inhibited division protein B	1.84		3.25
NMB0205	ferric uptake regulation protein*	1.91		2.47
NMB0228	conserved hypothetical protein	1.60		1.58
NMB0290	transcriptional regulator, putative	2.50		6.36
NMB0308	dihydrofolate reductase	1.75		1.62
NMB0334	glucose-6-phosphate isomerase	2.38		3.22
NMB0352	sugar isomerase, KpsF/GutQ family	1.54		0.14
NMB0358	shikimate 5-dehydrogenase	1.83		2.50
NMB0394	quinolinate synthetase A	1.89		1.63
NMB0398	transcriptional regulator, ArsR family	1.85		1.49
NMB0399	exodeoxyribonuclease III	1.82		2.50
NMB0427	cell division protein FtsZ	1.64		2.24
NMB0430	carboxyphosphonoenolpyruvate phosphonomutase, putative	1.69		1.26
NMB0431	methylcitrate synthase/citrate synthase 2	2.30		4.07
NMB0461	transferrin-binding protein 1	1.59		1.47
NMB0516	hypothetical protein	1.59		0.59
NMB0548	AcrA/AcrE family protein	1.50		1.68
NMB0549	ABC transporter, ATP-binding protein	1.69		3.10
NMB0556	repressor protein, putative*	2.71		3.45
NMB0586	adhesin, putative	1.94		1.49
NMB0618	phosphoenolpyruvate synthase	1.62		1.65
NMB0671	malate oxidoreductase (NAD)	1.68		3.42
NMB0681	conserved hypothetical protein	1.53		1.73
NMB0711	conserved hypothetical protein	1.81		2.69
NMB0740	DNA repair protein RecN*	3.51		4.16
NMB0756	dTDP-L-rhamnose synthase, putative	2.31		4.23
NMB0765	signal peptidase I	1.60		1.87
NMB0808	hypothetical protein	1.96		1.59
NMB0821	hypothetical protein	1.74		1.43
NMB0845	PhoH-related protein	1.89		2.07
NMB0865	hypothetical protein	1.64		2.07
NMB0872	conserved hypothetical protein	1.58		0.75
NMB0882	hypothetical protein	1.66		2.77

NMB0896	integrase, authentic frameshift	1.94		2.25
NMB0916	hypothetical protein	1.77		3.92
NMB0917	death-on-curing protein	1.50		0.20
NMB0921	hypothetical protein	2.57		3.11
NMB0929	dihydrodipicolinate synthase	1.81		3.32
NMB0942	50S ribosomal protein L31, putative	1.84		1.66
NMB0953	hypothetical protein	2.31		4.63
NMB0954	citrate synthase	2.41		1.10
NMB0997	D-lactate dehydrogenase	1.81		2.01
NMB1024	conserved hypothetical protein	1.56		2.19
NMB1026	conserved hypothetical protein	1.57		0.82
NMB1048	hypothetical protein	1.85		2.45
NMB1055	serine hydroxymethyltransferase	1.53		0.47
NMB1076	DnaA-related protein	1.60		0.52
NMB1088	conserved hypothetical protein	1.79		3.39
NMB1110	tail protein, 43 kDa	1.57		0.88
NMB1117	hypothetical protein	2.45		1.55
NMB1127	oxidoreductase, short chain dehydrogenase/reductase family	1.62		2.12
NMB1158	nickel-dependent hydrogenase, b-type cytochrome subunit	2.41		4.12
NMB1159	conserved hypothetical protein	1.54		1.55
NMB1209	hypothetical protein	1.57		1.28
NMB1218	conserved hypothetical protein	1.64		2.33
NMB1220	stomatin/Mec-2 family protein	1.77		3.51
NMB1221	hypothetical protein	1.67		1.63
NMB1226	ABC transporter, ATP-binding protein	1.56		0.73
NMB1295	formamidopyrimidine-DNA glycosylase	1.67		2.61
NMB1306	conserved hypothetical protein	1.63		2.91
NMB1360	pyridoxamine 5-phosphate oxidase	1.85		2.41
NMB1370	hypothetical protein	1.50		0.85
NMB1387	hypothetical protein	1.67		2.69
NMB1395	alcohol dehydrogenase, zinc-containing*	2.23		4.89
NMB1405	FrpA/C-related protein	1.58		0.76
NMB1417	conserved hypothetical protein	1.85		3.54
NMB1428	aminopeptidase, putative	1.89		3.24
NMB1458	fumarate hydratase, class II, aerobic	2.87		4.90
NMB1466	conserved hypothetical protein	1.72		2.67
NMB1475	conserved hypothetical protein	1.55		1.91
NMB1482	acyl CoA thioester hydrolase family protein	1.83		2.02
NMB1491	hypothetical protein	1.62		0.95
NMB1500	conserved hypothetical protein	3.25		7.04
NMB1529	hypothetical protein	1.66		3.21
NMB1530	succinyl-diaminopimelate desuccinylase	1.61		2.06
NMB1531	conserved hypothetical protein	1.54		0.46
NMB1540	lactoferrin-binding protein A*	2.33	3.39± 0.47	5.51
NMB1541	lactoferrin-binding protein B*	2.22	2.26± 0.63	4.10
NMB1572	aconitate hydratase 2	1.69		1.82
NMB1590	conserved hypothetical protein	3.89		5.22
NMB1609	trans-sulfuration enzyme family protein	1.64		1.03
NMB1620	conserved hypothetical protein	1.54		2.24
NMB1622	nitric oxide reductase	1.87		4.08
NMB1665	conserved hypothetical protein	1.54		1.22
NMB1684	seryl-tRNA synthetase	1.93		4.40
NMB1692	chorismate mutase-related protein	1.92		4.78
NMB1727	conserved hypothetical protein	1.75		2.41
NMB1735	GTP pyrophosphokinase	1.50		0.91
NMB1839	formate--tetrahydrofolate ligase	2.22		5.05
NMB1840	conserved hypothetical protein	2.58		6.21
NMB1868	integrase/recombinase XerC	1.71		3.06
NMB1871	conserved hypothetical protein	1.63		2.38
NMB1872	ribosomal-protein-alanine acetyltransferase, putative	1.52		1.96
NMB1879	hypothetical protein	1.74		2.79
NMB1890	conserved hypothetical protein	2.19		3.89
NMB1891	helix-turn-helix family protein	2.43		0.82
NMB1892	hypothetical protein	1.65		1.96
NMB1898	lipoprotein*	2.48	2.24± 0.58	4.32
NMB1909	Maf/YceF/YhdE family protein	1.87		2.73
NMB1957	hypothetical protein	1.64		1.99
NMB1969	serotype-1-specific antigen, putative	1.58		2.64
NMB1989	iron(III) ABC transporter, periplasmic binding protein	1.51		1.98
NMB1995	nitrogen regulatory protein P-II	1.66		1.38
NMB2009	ATP-dependent RNA helicase HrpA, degenerate	1.54		1.49
NMB2019	lipopolysaccharide core biosynthesis protein KdtB	1.75		0.03

NMB2037	hypothetical protein	1.71	2.25
NMB2043	IS1106 transposase, putative, degenerate	2.46	3.85
NMB2050	conserved hypothetical protein	1.78	3.44
NMB2078	conserved hypothetical protein	1.65	2.34
NMB2090	phosphoheptose isomerase	2.30	5.15
NMB2091	hemolysin, putative	1.90	4.67
NMB2142	conserved hypothetical protein	1.71	3.33

The genes listed are either down- or up- regulated in the *N. meningitidis* MC58 *modA11::kan* mutant strain. The identity of the gene is indicated with the gene ID in the annotation of the *N. meningitidis* strain MC58 genome (TIGR). The average ratio presented is the mean of MC58 *modA11::kan* mutant: wild-type MC58 *modA11* ON from 6 replicate spots on 3 independent microarrays, incorporating a dye swap. Only those genes with an expression value above 1.5-fold were included in this study except for NMB0205 and NMB2091, which are shown in italics. *Genes have been shown to be Fur regulated [55, 56].
