# Supplementary material for: Phasevarions Mediate Random Switching of Gene Expression in Pathogenic Neisseria
Source: PLoS Pathog. 2009 Apr 24;5(4):e1000400. doi: 10.1371/journal.ppat.1000400 (PMC2667262; doi:10.1371/journal.ppat.1000400)
Supplement: Table S4 — Differentially expressed genes in N. meningitidis wild-type B6116/77 modA12 ON versus the mutant strain B6616/77 modA12::kan. The genes listed are either downregulated or upregulated in the N. meningitidis B6116/77 modA12::kan mutant strain. The identity of the gene is indicated with the gene ID in the annotation of the N. meningitidis strain MC58 and Z2491 genome (TIGR). The average ratio presented is the mean of B6116/77 modA12::kan mutant:wild-type B6116/77 modA12 ON from six replicate spots on seven independent microarrays, incorporating a dye swap. Only those genes with an expression value above 1.5-fold were included in this study except for NMA1581and NMB1206, which are shown in italics. (0.11 MB PDF) [file ppat.1000400.s012.pdf]

Table S4. Differentially expressed genes in *N. meningitidis* wild-type B6116/77 *modA12* ON versus the mutant strain B6616/77 *modA12::kan*

Gene ID	Gene	Ratio	Qrt-pcr	B-stat
	**Reduced expression in *N. meningitidis modA12* mutant**			
NMA1203	hypothetical protein	0.58		6.39
NMA1581	*membrane lipoprotein*	*0.72*	*0.65± 0.14*	*4.35*
NMA1590	type III restriction/modification system modification methylase	0.13	0.07± 0.002	2.06
NMA1591	type III restriction/modification system enzyme	0.67		2.45
NMA1670	fumarate hydratase	0.64		5.28
NMB0292	conserved hypothetical protein	0.61		2.41
NMB0995	macrophage infectivity potentiator-related protein	0.65		2.99
NMB1017	sulfate ABC transporter, periplasmic sulfate-binding protein	0.59		4.40
NMB1070	2-isopropylmalate synthase	0.64		6.51
NMB1191	sulfate adenylyltransferase, subunit 1	0.67		1.92
NMB1426	hypothetical protein	0.44		7.78
NMB1458	fumarate hydratase, class II, aerobic	0.60		3.59
	**Increased expression in *N. meningitidis modA12* mutant**			
NMA1623	hypothetical protein	1.72		6.09
NMA2088	nicotinate-nucleotide pyrophosphorylase	2.05		7.19
NMB0245	NADH dehydrogenase I, E subunit	1.57		4.45
NMB0246	NADH dehydrogenase I, F subunit	1.55		7.28
NMB0396	nicotinate-nucleotide pyrophosphorylase	1.62		3.36
NMB0950	succinate dehydrogenase, flavoprotein subunit	1.67		5.21
NMB0951	succinate dehydrogenase, iron-sulfur protein	1.83	2.61± 0.46	2.90
NMB0952	conserved hypothetical protein	1.68		2.46
NMB0953	hypothetical protein	1.76		6.87
NMB0954	citrate synthase	1.53		3.96
NMB1206	*bacterioferritin B*	*1.42*	*2.14± 0.35*	*2.48*
NMB1403	FrpA-C-related protein	1.65		5.40
NMB1405	FrpA-C-related protein	1.73	2.21± 0.49	4.25
NMB1424	hypothetical protein	1.54		3.57

The genes listed are either down- or up- regulated in the *N. meningitidis* B6116/77 *modA12::kan* mutant strain. The identity of the gene is indicated with the gene ID in the annotation of the *N. meningitidis* strain MC58 and Z2491 genome (TIGR). The average ratio presented is the mean of B6116/77 *modA12::kan* mutant:wild-type B6116/77 *modA12* ON from six replicate spots on seven independent microarrays, incorporating a dye swap. Only those genes with an expression value above 1.5-fold were included in this study except for NMA1581and NMB1206, which are shown in italics.
