# Supplementary material for: Phasevarions Mediate Random Switching of Gene Expression in Pathogenic Neisseria
Source: PLoS Pathog. 2009 Apr 24;5(4):e1000400. doi: 10.1371/journal.ppat.1000400 (PMC2667262; doi:10.1371/journal.ppat.1000400)
Supplement: Table S5 — Differentially expressed genes in N. gonorrhoeae wild-type FA1090 modA13 ON versus the mutant strain FA1090 modA13::kan. The genes listed are either downregulated or upregulated in the N. gonorrhoeae FA1090 modA13::kan mutant strain. The identity of the gene is indicated with the gene ID in the annotation of the N. gonorrhoeae genome (TIGR). The average ratio presented is the mean of FA1090 modA13::kan mutant:wild-type FA1090 modA13 ON from six replicate spots on three independent microarrays, incorporating a dye swap. Only those genes with an expression value above 1.5-fold were included in this study. *Genes have been shown to be Fur regulated [56]. (0.12 MB PDF) [file ppat.1000400.s013.pdf]

**Table S5. Differentially expressed genes in *N. gonorrhoeae* wild-type FA1090 *modA13* ON versus the mutant strain FA1090 *modA13::kan*.**

| Gene ID | Gene | Ratio | Qrt-pcr | B-stat |
|---|---|---|---|---|
| | **Reduced expression in *N. gonorrhoeae modA13* mutant** | | | |
| NGO0055 | PilC2 | 0.62 | | 0.25 |
| NGO0554 | hypothetical protein | 0.49 | | 2.56 |
| NGO0574 | carbonic anhydrase | 0.58 | | 0.15 |
| NGO0641 | type III mod1 Modification methylas | 0.55 | | 3.02 |
| NGO0714 | 6-phosphogluconate dehydratase | 0.67 | | 1.28 |
| NGO0754 | molybdopterin-guanine dinucleotide biosynthesis protein | 0.55 | | 3.91 |
| NGO0794 | bacterioferritin | 0.59 | | 1.59 |
| NGO0867 | Helix-turn-helix domain protein possible transcriptional regulator | 0.57 | | 1.26 |
| NGO1247 | CDP-diacylglycerol-glycerol-3-phosphate 3-phosphatidyltransferase | 0.63 | | 1.22 |
| NGO1258 | phosphoglycerate mutase | 0.64 | | 1.28 |
| NGO1318 | haem utilisation protein | 0.61 | | 2.58 |
| NGO1365 | membrane fusion protein mtrc precursor | 0.62 | | 1.33 |
| NGO1442 | alcohol dehydrogenase, propanol-preferring (zn dependent) | 0.52 | | 3.21 |
| NGO1581 | phosphate permease, putative | 0.29 | | 8.53 |
| NGO1587 | adhesin MafB2 | 0.61 | | 2.57 |
| NGO1701 | Tat (twin-arginine translocation) pathway signal sequence domain protein | 0.6 | | 1.40 |
| NGO1931 | glyceraldehyde-3-phosphate dehydrogenase, type I | 0.37 | | 5.10 |
| NGO1947 | putative periplasmic protein | 0.67 | | 1.12 |
| NGO2062 | truncated silent pilin gene | 0.42 | | 6.27 |
| NGO2066 | pilin silent gene cassette | 0.41 | | 5.11 |
| | **Increased expression in *N. gonorrhoeae modA13* mutant** | | | |
| NGO0057 | thioredoxin | 1.71 | | 3.34 |
| NGO0198 | ammonium transporter | 3.18 | | 6.45 |
| NGO0318 | DNA repair protein RecN | 1.98 | | 2.02 |
| NGO0340 | cysteine synthase | 2.23 | | 4.20 |
| NGO0372 | amino acid ABC transporter, periplasmic amino acid-binding protein | 2.27 | | 3.28 |
| NGO0373 | amino acid ABC transporter, permease protein | 3.01 | | 5.47 |
| NGO0374 | amino acid ABC transporter, ATP-binding protein | 2.81 | | 7.14 |
| NGO0376 | peptidyl-prolyl cis-trans isomerase | 1.77 | | 2.00 |
| NGO0584 | ribosomal protein L9* | 1.64 | | 2.07 |
| NGO0624 | putative oxidoreductase | 1.58 | | 1.81 |
| NGO0650 | ATP-dependent RNA helicase, putative | 3.14 | | 6.62 |
| NGO0655 | exodeoxyribonuclease VII, large subunit | 3.16 | | 5.91 |
| NGO0656 | oxalate/formate antiporter | 2.39 | | 6.04 |
| NGO0659 | CDP-6-deoxy-delta-3,4-glucoseen reductase, putative | 1.53 | | 1.65 |
| NGO0768 | hypothetical protein | 1.61 | | 2.49 |
| NGO0775 | ATP-dependent protease La | 1.87 | | 1.37 |
| NGO0878 | sulfate ABC transporter, periplasmic sulfate-binding protein | 1.62 | | 0.54 |
| NGO0927 | Neisseria specific protein conserved hypothetical protein | 2.93 | | 2.46 |
| NGO0928 | 5-methyltetrahydropteroyltriglutamate | 3.01 | | 6.59 |
| NGO0929 | 5,10-methylenetetrahydrofolate reductase | 4.92 | | 8.32 |
| NGO1046 | ClpB protein endopeptidase | 1.9 | | 2.82 |
| NGO1220 | Membrane protein of unknown function (DUF340) family | 1.84 | | 1.41 |
| NGO1355 | sodium- and chloride-dependent transporter | 2.17 | | 2.21 |
| NGO1368 | efflux pump component MtrF | 2.2 | | 5.51 |
| NGO1511 | bicyclomycin resistance protein, putative | 1.75 | | 0.05 |
| NGO1769 | cytochrome c551 peroxidase | 1.62 | | 2.54 |
| NGO1822 | preprotein translocase SECY subunit | 1.76 | | 1.38 |
| NGO1827 | 50S ribosomal protein L5 | 1.68 | | 2.65 |
| NGO1836 | ribosomal protein L23* | 1.52 | | 0.74 |
| NGO1843 | translation elongation factor G | 1.79 | | 1.86 |
| NGO1851 | DNA-directed RNA polymerase, beta subunit* | 1.58 | | 1.69 |
| NGO1901 | chaperone protein dnaj | 1.71 | | 3.20 |
| NGO2011 | amino acid ABC transporter, permease protein glnM | 1.51 | | 1.45 |
| NGO2012 | amino acid ABC transporter, permease protein glnP | 1.75 | | 3.19 |

The genes listed are either down- or up- regulated in the *N. gonorrhoeae* FA1090 *modA13::kan* mutant strain. The identity of the gene is indicated with the gene ID in the annotation of the *N. gonorrhoeae* genome (TIGR). The average ratio presented is the mean of FA1090 *modA13::kan* mutant:wild-type FA1090 *modA13* ON from six replicate spots

on three independent microarrays, incorporating a dye swap. Only those genes with an expression value above 1.5-fold were included in this study. *Genes have been shown to be Fur regulated [56].
