# Supplementary material for: Phasevarions Mediate Random Switching of Gene Expression in Pathogenic Neisseria
Source: PLoS Pathog. 2009 Apr 24;5(4):e1000400. doi: 10.1371/journal.ppat.1000400 (PMC2667262; doi:10.1371/journal.ppat.1000400)
Supplement: Table S6 — Differentially expressed genes in N. gonorrhoeae wild-type 96D551 modA12 ON versus the mutant strain 96D551 modA12::kan. The genes listed are either downregulated or upregulated in the N. gonorrhoeae 96D551 modA12::kan mutant strain. The identity of the gene is indicated with the gene ID in the annotation of the N. gonorrhoeae genome (TIGR). The average ratio presented is the mean of 96D551 modA12::kan mutant:wild-type 96D551 modA12 ON from six replicate spots on three independent microarrays, incorporating a dye swap. Only those genes with an expression value above 1.5-fold were included in this study. (0.08 MB PDF) [file ppat.1000400.s014.pdf]

Table S6. Differentially expressed genes in *N. gonorrhoeae* wild-type 96D551 *modA12* ON versus the mutant strain 96D551 *modA12::kan*

Gene ID	Description	Ratio	Qrt-pcr	B-stat
	**Reduced expression in *N. gonorrhoeae modA12* mutant**			
NGO0640	RmsR	0.47		1.69
NGO0797	putative transcriptional regulator	0.48		1.07
NGO1236	acetolactate synthase	0.66		0.16
NGO2090	putative ABC transporter, permease protein, enterobactin	0.44		1.72
NGO2092	ferric enterobactin periplasmic binding protein	0.62		0.61
NGO2093	FetA	0.24	2.5± 0.056	2.51
	**Increased expression in *N. gonorrhoeae modA12* mutant**			
NGO0365	site-specific DNA-methyltransferase M.NgoVII	1.78		0.74
NGO0364	restriction endonuclease R.NgoVII	1.60		0.33
NGO0861	hypothetical protein	2.43		2.01
NGO0860	hypothetical protein	2.03		1.64
NGO1046	putative ClpB protein	1.64		0.33
NGO1516	quinolinate synthetase A	1.70		0.90

The genes listed are either down- or up- regulated in the *N. gonorrhoeae* 96D551 *modA12::kan* mutant strain. The identity of the gene is indicated with the gene ID in the annotation of the *N. gonorrhoeae* genome (TIGR). The average ratio presented is the mean of 96D551 *modA12::kan* mutant:wild-type 96D551 *modA12* ON from six replicate spots on three independent microarrays, incorporating a dye swap. Only those genes with an expression value above 1.5-fold were included in this study.
