# Supplementary material for: Phasevarions Mediate Random Switching of Gene Expression in Pathogenic Neisseria
Source: PLoS Pathog. 2009 Apr 24;5(4):e1000400. doi: 10.1371/journal.ppat.1000400 (PMC2667262; doi:10.1371/journal.ppat.1000400)
Supplement: Table S7 — Primers used to synthesize probes for Southern analysis, mod allele study, and sequencing plasmid pCmGFP. aQRT-PCR primers used for N. meningitidismodA11 study. bQRT-PCR Primers used for N. meningitidismodA12 and N. gonorrhoeaemodA12 study. cQRT-PCR primers used for N. gonorrhoeaemodA13 study. QRT-PCR primers are named after their TIGR gene ID. (0.06 MB PDF) [file ppat.1000400.s015.pdf]

Table S7. Primers used to synthesize probes for Southern Analysis, *mod* allele study and sequencing plasmid pCmGFP

Primer	Sequence 5'-3'
ApoI LHS1for	AAGGTTTGAAGATACCGCACACC
ApoI LHS1rev	CTGCCGTAAGGTTTCCGTTATTC
HindIII-1 for	CGTGCTGATGGTGATGATAGTCG
HindIII-1 rev	TGGTTTTACTGAGGGGAATGTGG
AluI –T for	TCACAGTACCGACATTACGTCG
AluI – T rev	ATGAAAACACTACAAGACTGGCTCTCG
ModAF	ATGGCGGACAAAGCACCGAAGA
ModAF6Fam	mATGGCGGACAAAGCACCGAAGA
ModAR	TTCGCCATCTTTTTTCTCCGCTTG
ModAREPEATR	CAAAAAGCCGGTCAATTTCATCAAA
ModADRDF	GACGCAAAAGCATTCTCAAAAT
ModADRDR	GCGTTCAACGGCAAGCGTTTC
ModAF2	ATGAAGACAGACATTCAAACCG
ModBREPEATF	CACGCAGACAGACGTTCAGACG
ModB REPEATR	TTTCCGTCTGCGTCAAAACAGGCG
ModBDRDF	CTTCGCAACCTGCCGCCCG
ModBDRDR	CGGGCGAAAAATCCTTGTCC
ResF	CTATCAATCAGTAAAAAATCAAGCG
ResR	CAAACGCAAATGGAAAATAACGC
ResEDF2:	GCGTTATTTTCCATTTGCGTTTG
ResEDR2:	CTACTTCATTTTTTCAAGCGATGCC
KanF	AAGCCACGTTGTGTCTC
KanR	CTGCCTCGTGAAGAAGG
16SF	ACGGAGGGTGCGAGCGTTAATC
16SR	CTGCCTTCGCCTTCGGTATTCCT
NMB0014F [a]	AACCGAAATCTGGCCCAAC
NMB0014R [a]	CAACGATTTTTCCGACAGCC
NMB0144F [a]	CTGCTGTTCGGCGTTCAAG
NMB0144R [a]	TCGCTGCGACGACCTAAAGT
NMB0148F [a]	ATGCTTCGGTTGGTCGAGTG
NMB0148R [a]	TTACCGATAACCACACCCGG
NMB0155F [a]	GAGGAAGAGCGTTTTGAGGCT
NMB0155R [a]	CTGTCAAAGCACAACGACGAC
NMB1540F [a]	GCTTCGGTTACGATGCTTCC
NMB1540R [b]	TCATCGAATCCCGTGGATTC
NMB1541F [a]	TTCTTATGAGGCTAGGGATGCC
NMB1541R [a]	CAGCTTGCCATTCAGGGTTT
NMB1898F [a]	CCCTATTCCGTAACGCTCGA
NMB1898R [a]	TGTCGTCTTTGGTCAGGCC
LbpBF	CGGAGCGGTTTCTGCTTTTC
LbpBR	TGTATCTGCTGCGTTTTCTGTCTC
LacZ*Sty*I+1	GTATACCCTTGGTGATCCCGTCGTTTTACAA
Kan*Sty*I	GGTCCCTTGGCAATTCTGATTAGAAAAACTC
NMB1405F [b]	AAACTGGCAGACGGTTCTTTTG
NMB1405R [b]	ACACGCAGGGATTGGAATGC
NMB0951F [b]	CGCCTGAAAGCACAAGACGATAC

NMB0951R [b]	CAAGCCGTTTTTGCCGTTG
NMB1206F [b]	AGCTTCTGATTGGCGAGTCCA
NMB1206R [b]	TGCTGTGGCGATGGCGGCA
NMB1581F [b]	GAACAGCAGCAGAAACAGATTG
NMB1581R [b]	CGGCAGGAATGGTGTCTTTGAC
NMB1590F [b]	CGTTGCCCACAAAATGAACC
NMB1590R [b]	CCTTGCTCGCCATCAATCAC
NGO2093F [b]	GAGATTGACGGCTTTACCCTG
NGO2093R [b]	TGAGGCTGCTGCTTGAAACG
NGO0198F [c]	GCTGGCTTTATTCTACGGCG
NGO0198R [c]	GATGCCCACCAATGTCGC
NGO0372F [c]	ACCGGTTACGACGTGGAAGTA
NGO0372R [c]	CCATCATCGAATCCCATTGC
NGO0373F [c]	CTGCCGTTTATGACGGAAACA
NGO0373R [c]	GCCAAAGGCAAAGACACTGTAA
NGO0374F [c]	GCGATTCAGCCTGAATTGATG
NGO0374R [c]	ATTCCTTCATGGCGTCCAAC
NGO0554F [c]	GCTGCTTTCGGCTCTGCTTAT
NGO0554R [c]	GGTAGGAAATCGGATTCGGC
NGO0656F [c]	GGCACAGGCATACAGCGTAA
NGO0656R [c]	TTCGTGTACGGATTTGACGG
NGO0927F [c]	TCCTGAGTTGCGGCGC
NGO0927R [c]	CCTGTTCCCCACATATAAAGCC
NGO0928F [c]	TCTTCGGTGACGTAAGCCG
NGO0928R [c]	AACATACCTTTCATCGGGCG
NGO0929F [c]	ACTTACGGCGCAAACTCCG
NGO0929R [c]	AGGTGCGAAGCTGCTTCC
NGO1368F [c]	GTGCGTATTTCGGACTATCCG
NGO1368R [c]	AGCAGGCTGACAACGTGAATC
NGO1931F [c]	CTTCCTGTACCACCAACTGCC
NGO1931R [c]	GCGTGGATGGTGGTCATCA
NGO1581F [c]	CACCGTCATCATCTGTCCGA
NGO1581R [c]	TGCAGCGCCTTGTAGGAATC
GfpUpOut	TTCCGTATGTTGCATCACCTTCACCCTCTCC
GfpDownOut	TGTAACAGCTGCTGGGATTACACATGGCATGG
GfpA	ATCCCACAAGTTTGTGAAGTCTTAACTGC
GfpB	TTCTTGTTGAATTAGATGG
GfpC	TAGCTTGCATGCCTGCAGGAGG
GfpD	AACCGTTCAGCTGGATATTACG
GfpE	TTGCTGTATTTCGCTCTGCCGCTCTAACTCG
GfpF	TTGAAGATGGAAGCGTTCAACTAGCAGACC
GfpG	TAGCTTGCCTGATAGGGCTAGG
GfpH	TTTCCATGAGCAAACTGAAACG
GfpI	GCGTAATCGCTGCTGTTTATCAGG
GfpJ	TGCTCAATGCGGAACGCCCGAATATCG
GfpK	AAGATGGAAACATTCTTGGACACAAATTGG
GfpL	TTGTTGCTCTATGCTGGCGGCTTCGGTGC
GfpM	TTGGAATTTGACGGTTTTTTTGTCGGATGC
GfpN	TTCAGCTGGATATTACGGCCTTTTTAAAGACC
GfpO	TTGAACGATGTGGGACAGTTTGTATTGC
GfpW	TATTGCGTGTGTTCGCCGCTTTCC
GfpX	AAGTCGCAAACTCGGTCGGGCTGACTCG

GfpY	AAGTGGCGCGGGAAATGCCGAAGTGTTCC
GfpZ	AAGCCATACTGCAAAGATTTCAAAGAAGC
mod16.for	CATGCCATGGGCCATCATCATCATCATCATCATCATCATCACATGAAGAC AGACATTCAAACCGAATTA
mod16.rev	CGCGGATCCGCGTCATTCGCCATCTTTTTCTCCGCTTGATA

[a] QRT-PCR Primers used for *N. meningitidis modA11* study.
[b] QRT-PCR Primers used for *N. meningitidis modA12* and *N. gonorrhoeae modA12* study.
[c] QRT-PCR Primers used for *N. gonorrhoeae modA13* study.
QRT-PCR Primers are named after their TIGR gene ID.
