# Supplementary material for: Phasevarions Mediate Random Switching of Gene Expression in Pathogenic Neisseria
Source: PLoS Pathog. 2009 Apr 24;5(4):e1000400. doi: 10.1371/journal.ppat.1000400 (PMC2667262; doi:10.1371/journal.ppat.1000400)
Supplement: Table S8 — Fragment analysis on FA1090 modA13 ON/OFF original inoculum and 40, 50, and 60 ug/ml Triton X-100 concentrations (A) and FA1090 modA13 ON/OFF ratio Student's t-test results (B). Data represents genescan analysis results where the size of the repeat tract was determined using fluorescent primers (see Materials and Methods) and contains values determined from three independent samples[17]. (0.05 MB PDF) [file ppat.1000400.s016.pdf]

Table S8a. Fragment analysis on FA1090 *modA13* ON/OFF Original Inoculum and 40, 50 and 60 ug/ml Triton X-100 concentrations

Sample	% ON in FA1090 ON Inoculum	Average % ON or OFF	Std dev
FA1090 Inoculum ON_1	89.65		
FA1090 Inoculum ON_2	88.86		
FA1090 Inoculum ON_2	87.87		
	% ON in FA1090 ON 40 ug/ml	88.79	0.892
FA1090 *modA13* ON 40_1	62.54		
FA1090 *modA13* ON 40_2	40.89		
FA1090 *modA13* ON 40_3	55.58	53.01	11.05
	% ON in FA1090 ON 50 ug/ml		
FA1090 *modA13* ON 50_1	10.84		
FA1090 *modA13* ON 50_2	35.09		
FA1090 *modA13* ON 50_3	13.19	19.71	13.37
	% ON in FA1090 ON 60 ug/ml		
FA1090 *modA13* ON 60_1	24.37		
FA1090 *modA13* ON 60_2	10.05		
FA1090 *modA13* ON 60_3	25.12	19.85	8.49
	% OFF in FA1090 ON Inoculum		
FA1090Inoculum ON_1	10.35		
FA1090Inoculum ON_2	11.14		
FA1090Inoculum ON_3	12.13	11.21	0.892
	% OFF in FA1090 ON 40 ug/ml		
FA1090 *modA13* ON 40_1	37.46		
FA1090 *modA13* ON 40_2	59.11		
FA1090 *modA13* ON 40_3	44.42	46.99	11.05
	% OFF in FA1090 ON 50 ug/ml		
FA1090 *modA13* ON 50_1	89.16		
FA1090 *modA13* ON 50_2	64.91		
FA1090 *modA13* ON 50_3	86.81	80.29	13.37
	% OFF in FA1090 ON 60 ug/ml		
FA1090 *modA13* ON 60_1	75.63		
FA1090 *modA13* ON 60_2	89.95		
FA1090 *modA13* ON 60_3	74.88	80.15	8.49

Table S8b. FA1090 *ModA13* ON/OFF Ratio Student's *t*-test Results

	P-value
% ON in FA1090ON Inoculum vs % ON in FA1090 ON 40 ug/ml	0.02967
% OFF in FA1090ON Inoculum vs % OFF in FA1090ON 40 ug/ml	0.02967
% ON in FA1090ON Inoculum vs % ON in FA1090 ON 50 ug/ml	0.01198
% OFF in FA1090ON Inoculum vs % OFF in FA1090ON 50 ug/ml	0.01198
% ON in FA1090ON Inoculum vs % ON in FA1090 ON 60 ug/ml	0.00465
% OFF in FA1090ON Inoculum vs % OFF in FA1090ON 60 ug/ml	0.00465

Data above represents genescan analysis results where the size of the repeat tract was determined using fluorescent primers (see Materials and Methods) and contains values determined from 3 independent samples [17].
