# Supplementary material for: Phasevarions Mediate Random Switching of Gene Expression in Pathogenic Neisseria
Source: PLoS Pathog. 2009 Apr 24;5(4):e1000400. doi: 10.1371/journal.ppat.1000400 (PMC2667262; doi:10.1371/journal.ppat.1000400)
Supplement: Table S9 — Fragment analysis of O1G1370 modA13 ON/OFF original inoculum and survival +3 h (A) and O1G1370 modA13 ON/OFF ratio student's t-test results (B). Data represents genescan analysis results where the size of the repeat tract was determined using fluorescent primers (see Materials and Methods) and contains values determined from three independent samples[17]. (0.05 MB PDF) [file ppat.1000400.s017.pdf]

Table S9a. Fragment analysis of O1G1370 *modA13* ON/OFF Original Inoculum and Survival+3hrs

Sample	% ON in O1G1370 ON Inoculum	Average % ON or OFF	Std dev
O1G1370Inoculum ON_1	92.84		
O1G1370Inoculum ON_2	91.98		
O1G1370Inoculum ON_3	97.60	94.14	3.03
	% ON in O1G1370 ON_survial+3		
O1G1370ON_1+3	55.91		
O1G1370ON_2+3	67.81		
O1G1370ON_3+3	26.76	50.16	21.12
	% OFF in O1G1370 ON Inoculum		
O1G1370Inoculum ON_1	7.16		
O1G1370Inoculum ON_2	8.02		
O1G1370Inoculum ON_3	2.40	5.86	3.03
	% OFF in O1G1370 ON_survial+3		
O1G1370ON_1+3	44.09		
O1G1370ON_2+3	32.20		
O1G1370ON_3+3	73.24	49.84	21.12
	% ON in O1G1370 OFF Inoculum		
O1G1370Inoculum OFF_1	7.45		
O1G1370Inoculum OFF_2	7.21		
O1G1370Inoculum OFF_3	9.10	7.92	1.03
	% ON in O1G1370 OFF_survial+3		
O1G1370OFF_1+3	1.46		
O1G1370OFF_2+3	11.91		
O1G1370OFF_3+3	9.52	7.63	5.47
	% OFF in O1G1370 OFF Inoculum		
O1G1370Inoculum OFF_1	92.54		
O1G1370Inoculum OFF_2	92.79		
O1G1370Inoculum OFF_3	90.89	92.07	1.03
	% OFF in O1G1370 OFF_survial+3		
O1G1370OFF_1+3	98.54		
O1G1370OFF_2+3	88.09		
O1G1370OFF_33	90.48	93.37	5.47

Table S9b. O1G1370 *ModA13* ON/OFF Ratio Student's *t*-test Results

	P-value
% ON in O1G1370ON Inoculum vs % ON in O1G1370 ON_survial+3	0.023357
% OFF in O1G1370ON Inoculum vs % OFF in O1G1370 ON_survial+3	0.023357
% ON in O1G1370OFF Inoculum vs % ON in O1G1370 OFF_survial+3	0.932341
% OFF in O1G1370OFF Inoculum vs % OFF in O1G1370 OFF_survial+3	0.932341

Data above represents genescan analysis results where the size of the repeat tract was determined using fluorescent primers (see Materials and Methods) and contains values determined from 3 independent samples [17].
